# Kabirimine, a New Cyclic Imine from an Okinawan Dinoflagellate

**DOI:** 10.3390/md17060353

**Published:** 2019-06-13

**Authors:** Idam Hermawan, Mikako Higa, Philipus Uli Basa Hutabarat, Takeshi Fujiwara, Kiyotaka Akiyama, Akihiko Kanamoto, Takahiro Haruyama, Nobuyuki Kobayashi, Masahiro Higashi, Shoichiro Suda, Junichi Tanaka

**Affiliations:** 1 Graduate School of Engineering and Science, University of the Ryukyus, Senbaru 1, Nishihara, Okinawa 903-0213, Japan; hermawan_idam@yahoo.com (I.H.); k198365@eve.u-ryukyu.ac.jp (M.H.); philipus.hutabarat@gmail.com (P.U.B.H.); 2 OP Bio Factory Co., Ltd., Okinawa Life Science Center 107, 5-8 Suzaki, Uruma, Okinawa 904-02234, Japan; takeshi.fujiwara@opbio.com (T.F.); kiyotaka.akiyama@opbio.com (K.A.); akihiko.kanamoto@opbio.com (A.K.); 3 Central Research Center, AVSS Corporation, Nagasaki 852-8137, Japan; haruyama@avss.jp (T.H.); kobayashi@avss.jp (N.K.); 4 Department of Molecular Engineering, Kyoto University, Kyoto 615-8510, Japan; higashi@moleng.kyoto-u.ac.jp; 5 Department of Chemistry, Biology and Marine Science, University of the Ryukyus, Senbaru 1, Nishihara, Okinawa 903-0213, Japan; sudas@sci.u-ryukyu.ac.jp

**Keywords:** cyclic imine, dinoflagellate, respiratory syncytial virus (RSV), portimine, kabirimine

## Abstract

On our quest for new bioactive molecules from marine sources, two cyclic imines (**1**, **2**) were isolated from a dinoflagellate extract, inhibiting the growth of the respiratory syncytial virus (RSV). Compound **1** was identified as a known molecule portimine, while **2** was elucidated to be a new cyclic imine, named kabirimine. The absolute stereochemistry of **1** was determined by crystallographic work and chiral derivatization, whereas the structure of **2** was elucidated by means of spectroscopic analysis and computational study on all the possible isomers. Compound **1** showed potent cytotoxicity (CC_50_ < 0.097 µM) against HEp2 cells, while **2** exhibited moderate antiviral activity against RSV with IC_50_ = 4.20 µM (95% CI 3.31–5.33).

## 1. Introduction

Infectious diseases, such as influenza and hepatitis B caused by viruses, are still a major concern for human beings. The respiratory syncytial virus (RSV) causes infection at the lower respiratory tract, resulting in cold-like symptoms, while the infection may develop into severe illness for infants [1]. Although a number of supportive measures are prescribed for the patients, Palivizumab, a humanized monoclonal antibody, is the only medication against the disease with limited use. Therefore, searching antiviral molecules against RSV is beneficial for our society, and RSV was chosen as one of the targets on our drug discovery project [2]. 

After preparing a library of extracts both from sessile marine organisms, such as sponges and tunicates, as well as from marine microorganisms consisting of bacteria, fungi, and dinoflagellates, the extracts were screened against several viruses. As a result, we reported spongian class diterpenoids as moderate inhibitors against the adenovirus [3]. In the screening against RSV, moderate inhibition was observed for an extract coded OPMS30976 prepared from a cultured dinoflagellate *Vulcanodinium rugosum*, which was originally collected with a plankton net at Kabira Bay, Ishigaki, Okinawa. 

Dinoflagellates have been known for producing toxic molecules with complex structures, and some of them are known to cause seafood poisoning, such as ciguatera and diarrhetic shellfish poisonings [4]. The phycotoxins can be divided into several structural classes: tetrahydropurines, secondary amines, linear and macrocyclic or ladder-frame polyethers, and cyclic imines [5]. After the first discovery of prorocentrolide from *Prorocentrum lima* [6], a number of cyclic imines have been reported. Their structures are characterized by the presence of spirocyclic imine in a macrocyclic ring, and the molecules are grouped into several classes, such as pinnatoxins, pteriatoxins, gymnodimines, and spirolides [7]. In addition to their interesting structures, cyclic imines show a variety of biological activities. Pinnatoxins, gymnodimines, and spirolides were reported to block nicotinic acetylcholine receptors with subtype specificity caused by chemical variation around spirocyclic imine [8,9]. Due to their interesting structures and bioactivities, the spirocyclic imines also attract synthesis researchers [10,11].

In this article, we would like to report the isolation, structure elucidation, and bioactivity of a new cyclic imine named kabirimine (**2**) and the absolute stereochemistry of a known metabolite portimine (**1**), reported previously as an apoptosis-causing cytotoxin from a New Zealand specimen of *V*. *rugosum* [12].

## 2. Results and Discussion

**Figure 1:**
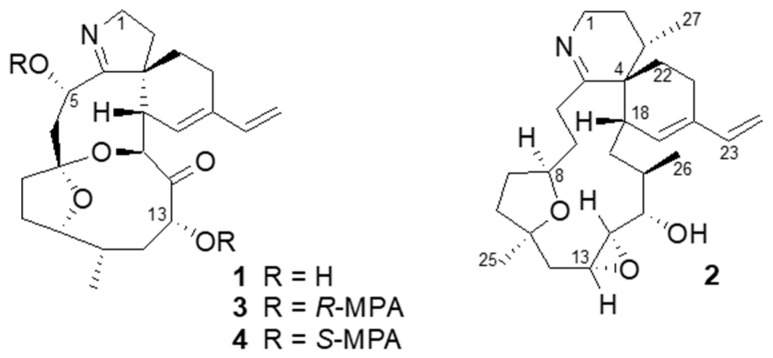
Structures of portimine (**1**) and kabirimine (**2**).

During the bioprospective study for antiviral molecules from marine dinoflagellates, known for the presence of structurally unique bioactive metabolites [13], an extract of a preliminary culture of the dinoflagellate *V*. *rugosum* was found to inhibit the growth of RSV. Since one out of twenty fractions after gradient HPLC separation of the extract was confirmed to show the activity, the strain was cultured in a larger quantity of IMK medium for three weeks. The extract was subjected to bioassay-guided, reversed-phase chromatographic separation to give portimine (**1**) and kabirimine (**2**) (Figure 1) in 1.4% and 0.52% yields, respectively. 

As cyclic imine toxins constitute a group of dinoflagellate metabolites [7], portimine (**1**) was readily identified by observing its molecular formula C_23_H_31_NO_5_ with high resolution electrospray ionization mass spectra (HRESIMS), and also by comparing the nuclear magnetic resonance (NMR) data (Figures S17 and S18) with those reported by Selwood and co-workers [12]. These authors characterized portimine as a cytotoxic metabolite of a New Zealand specimen of *V*. *rugosum* and elucidated its relative stereochemistry by analyzing the nuclear Overhauser effect (NOE) results. Since the absolute stereochemistry of portimine (**1**) has not been reported, we applied chiral derivatization and X-ray crystallography methods to determine the absolute configurations of its stereogenic carbons. 

**Figure 2:**
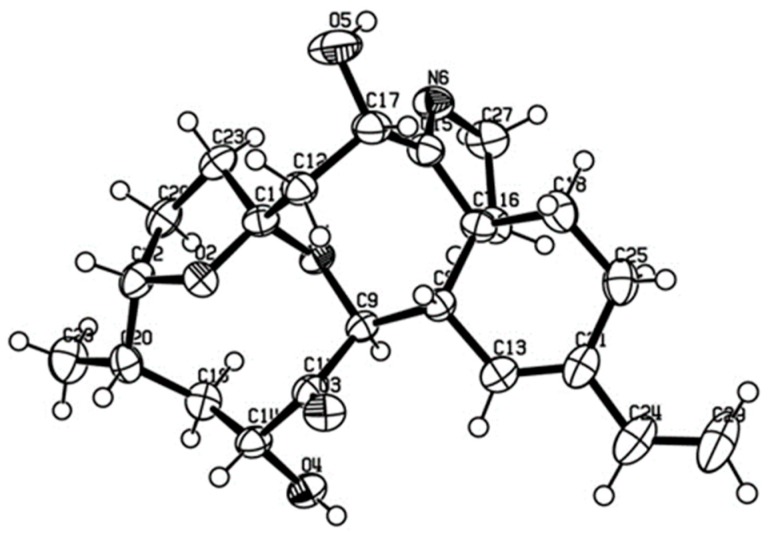
Oak Ridge Thermal-Ellipsoid Plot (ORTEP) diagram of portimine (**1**).

By treating portimine (**1**) with either *R*- or *S*-methoxyphenyl acetic acid (MPA) and a condensation reagent, esters **3** (*R*-) and **4** (*S*-) having two MPA moieties were obtained. By observing chemical shift differences (Δδ = δ**_3_** − δ**_4_**) for the portion around C-5 (Δδ +0.18 and +0.07 for H-1; −0.16 and −0.14 for H-6) and also for C-13 (Δδ +0.05 for H-12b, +0.02 for H-11; −0.05 for H-15, −0.08 for H-16, −0.15 for H-21), these chiral centers were determined as 5*S* and 13*R* [14]. In addition, our material gave suitable crystals for X-ray crystallography from an aqueous MeOH solution. As a result, the whole structure can be determined as shown in Figure 2, confirming the relative stereochemistry proposed by Selwood et al. [12]. In addition, the absolute stereochemistry is as shown in Figure 2, with the Flack parameter 0.11 ± 0.16. 

The molecular formula of **2** was established as C_27_H_41_NO_3_ from the HRESIMS measurement. The presence of a nitrogen atom and co-isolation with **1** suggested that **2** is an analog of **1** or a related cyclic imine [7]. The ^1^H and ^13^C NMR spectra of **2** (Figures S22–S29) showed signals for a terminal diene (δ_H_ 5.02, 5.13, 6.42, 5.70; δ_C_ 112.1, 138.3, 134.8, 129.4) and an epoxide (δ_H_ 2.66, 2.89; δ_C_ 53.7, 61.5) (Table 1). Therefore, there are five rings in the molecule out of eight degrees of unsaturation. The remaining signals include two secondary methyls (δ_H_ 0.96, 1.05), a tertiary methyl (δ_H_ 1.19), two oxymethines (δ_H_ 2.96, 3.93; δ_C_ 76.0, 72.5), two quaternary sp^3^ carbons (δ_C_ 48.1, 80.6), three sp^3^ methines (δ_C_ 25.3, 36.0, 38.7), and ten methylenes.

**Figure 3:**
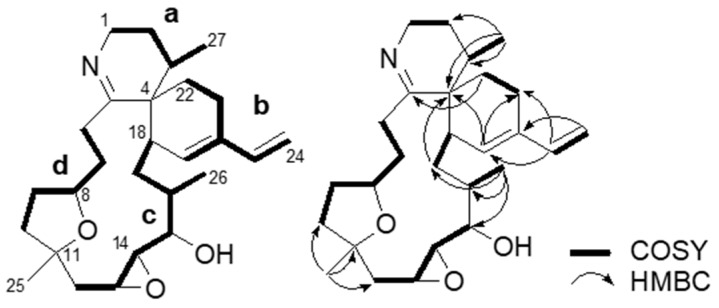
Partial structures **a**–**d** and NMR connectivity of kabirimine (**2**).

A downfield-shifted methylene (δ_H_ 3.77; δ_C_ 42.1, C-1), likely located next to the imine nitrogen, showed correlation spectroscopy (COSY) correlations to another methylene proton (δ_H_ 2.14 and 1.62, H-2), which connected to a methyl-bearing methine (δ_H_ 2.08, H-3). The methyl doublet (δ_H_ 0.96, *J* = 6.6 Hz, H-27) showed heteronuclear multiple bond correlation (HMBC) correlation cross peaks to the quaternary carbon at δ_C_ 48.1 (C-4), forming a partial structure, **a** (Figure 3). 

A cyclohexene ring with a conjugated vinyl group (partial structure **b**) was inferred by observing HMBC correlations (H-19/C-21, H-23/C-19,21, and H-24/C-20) and COSY correlations (H-21/H-22 and H-23/H-24). Additional HMBC correlations from H-22 to the imine carbon (C-5) and from the vinyl proton at δ_H_ 5.70 (H-19) to the quaternary carbon at δ_C_ 48.1 (C-4) support the presence of a spirocyclic imine moiety and a diene, as found for other cyclic imine toxins [7].

The connection from C-12 through C-18 corresponding to the partial structure **c** was supported by the following COSY (H-12/H-13, H-13/H-14, H-14/H-15, H-16/H-26, and H-17/H-18) and HMBC correlations (H-26/C-15,16,17). 

The partial structure **d** extending from allylic protons at δ_H_ 3.13 and 2.92 (H-6) to methylene protons at δ_H_ 1.75 and 1.63 (H-10) through an oxymethine proton at δ_H_ 3.93 (H-8) was inferred by COSY correlations (H-6/H-7, H-7/H-8, H-8/H-9, and H-9/H-10). As the methyl singlet at δ_H_ 1.19 (H-25) showed HMBC correlations to C-10,11,12, the partial structure **d** was connected to the partial structure **c**. Thus, the whole planar structure of **2** can be elucidated as shown in Figure 3, having a 15-membered carbocyclic ring. 

**Figure 4:**
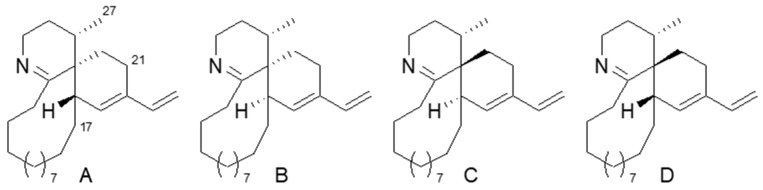
Structures of model molecules **A**–**D**.

A relative configurational analysis of **2** started from the spirocyclic portion. As there are three chiral centers in this portion, there are four possible isomers with 3S*,4S*,18S*, 3S*,4S*,18R*, 3S*,4R*,18R*, and 3S*,4R*,18S*. To find the most probable isomer, model molecules A–D, lacking functionalities on the 15-membered ring (Figure 4), were examined with molecular mechanics/molecular dynamics (MM/MD) calculation to give a relatively rigid conformation for the spirocyclic portion of A–D (Figure S34). In the nuclear Overhauser effect spectroscopy NOESY of **2**, a positive NOE was observed for H-17/H-27, while no NOE was found for H-21/H-27. The results are consistent with the estimated proton distances of D having a 3S*,4R*,18S* configuration, while A and C are not likely to have a positive NOE for H-17/H-27, and an NOE is expected for H-21/H-27 in A–C.

For the substituted tetrahydrofuran portion, a NOESY correlation between H-8 and H-25 indicates a *cis* relationship. The geometry of the epoxide was elucidated as *trans* due to the small coupling constant between H-13 and H-14. For the portion from C-14 to C-16, we tried to apply the *J*-based configurational analysis (JBCA) method [15]. Although a few coupling constants (^3^*J*_H15-H16_ = small, ^3^*J*_H15-C17_ = small and ^3^*J*_H15-C26_ = 7.3 Hz) were observed, it was not conclusive. Additional coupling constants (^3^*J*_H14-H15_ = 8.3 Hz, ^3^*J*_H15-C13_ = medium and ^2^*J*_H15-C14_ = 7.2 Hz) did not support a specific conformer either. As no useful NOE was observed between transannular protons in the 15-membered ring, a computational study was applied on all the possible isomers to compare their optical rotation values with that of a natural compound as a last resort. 

**Figure 5:**
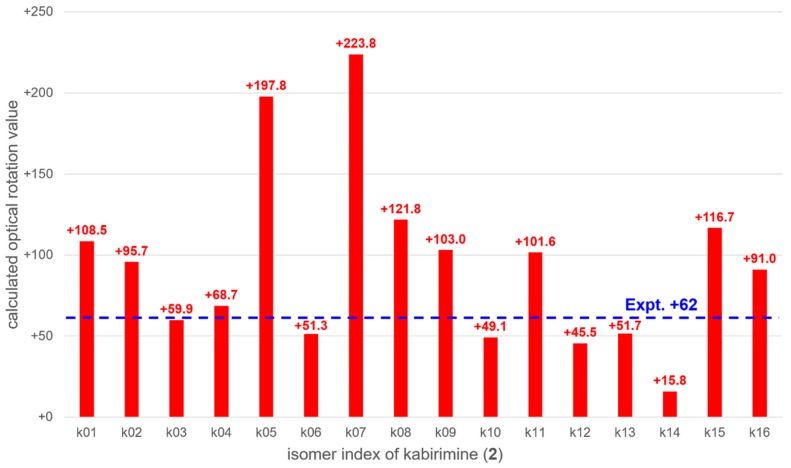
Comparison between calculated and experimental optical rotation values of kabirimine (**2**).

The optical rotation values of 16 possible isomers k01 to k16 (Tables S1–S16, Figures S1–S16) were calculated with the time-dependent density functional theory (TDDFT). Stable conformations of each isomer were obtained with the global reaction route mapping (GRRM) strategy [16,17]. The thermally averaged optical rotation values were summarized in Figure 5. It was found that the calculated optical rotation value of k03, +59.9, was the closest to the experimental value of **2**, +62, though several other isomers have similar optical rotation values. Therefore, it is likely that **2** has the k03 configuration, i.e., 3*S*, 4*R*, 8*R*, 11*R*, 13*R*, 14*R*, 15*S*, 16*R*, and 18*S*. Since **2** has not been previously described, it was named kabirimine after the collection site. 

The anti-RSV activity of portimine (**1**) and kabirimine (**2**) were examined. Although a fraction containing the imines displayed moderate inhibition during the isolation process, purified portimine (**1**) showed potent cytotoxicity, precluding antiviral evaluation, even at the lowest concentration tested (0.097 µM), as reported in [12]. Kabirimine (**2**) showed moderate antiviral activity with an IC_50_ = 4.20 µM (95% confidence interval, 3.31–5.31 µM) against RSV (Figure S33). 

## 3. Experimental Section 

### 3.1. General Experimental Procedures 

Both 1D and 2D NMR spectra were measured on a 500 MHz Bruker Avance III spectrometer (Massachusetts, USA). Chemical shifts were referenced to tetramethylsilane (TMS) signals. HRESIMS spectra were recorded on a Jeol JMR-T100LP mass spectrometer (Tokyo, Japan). FTIR and UV spectra were recorded on a Jasco FTIR-6100 instrument and a Jasco V-660 spectrophotometer (Tokyo, Japan). Optical rotation was measured on a Jasco P-1010 polarimeter. The X-ray diffraction study was conducted on a Rigaku R-AXIS RAPID II diffractometer (Tokyo, Japan) with graphite monochromated Cu Kα radiation. 

Gradient HPLC was performed on a unit with a Shimadzu Prominence LC-20AB pump (Kyoto, Japan), an SPD-M20 diode array detector, and a Chromolith Performance RP-18e (100–4.6 mm) HPLC column. Cosmosil 75C_18_-OPN was used for column chromatography. Reagent grade solvents were used for all experimental processes. 

### 3.2. Biological Material

The dinoflagellate *Vulcanodinium rugosum* was collected with a plankton net at Kabira Bay, Ishigaki, Okinawa in June 2012. The strain was coded as OPMS30976, brought back to the lab, and kept alive in a medium. The dinoflagellate was identified by one of us (SS). The specimen was deposited at an an OP Bio Factory. The dinoflagellate had a benthic nature, and almost no swimming cells appeared in the culture. DNA sequencing (LSU) and phylogenetic analysis were carried out as described by Prabowo et al. [18]. Morphological characteristics and phylogenetic analysis using a LSU rDNA sequence (D1 region, accession number: LC228963) confirmed that the dinoflagellate was *Vulcanodinium rugosum*.

### 3.3. Extraction and Isolation

An isolate of the microalga was cultured in 500 mL of IMK medium (Wako, 398-01333, Osaka, Japan). The collected alga was extracted with MeOH to give 25.3 mg of the crude MeOH extract. A portion of this extract was separated on a gradient HPLC using a Chromolith column with 95% aq MeOH to MeOH with 0.05% trifluoroacetic acid (TFA) to give twenty fractions. Among the fractions, the eighth fraction was found to show antiviral activity. 

To obtain the antiviral component, the alga was cultured again in 200 L of IMK medium for three weeks. The harvested alga was freeze-dried (32.84 g), and the whole was extracted four times with MeOH. The MeOH solution was concentrated to give a crude extract (1.5 g). A portion (554.8 mg) of the extract was applied to a C18 column to give twelve fractions. A precipitate was formed when a mixture of MeOH–water (1:1) was added to Fraction 3 (64.1 mg). After centrifugation, the supernatant (32.1 mg) was separated on a gradient HPLC in the same condition as above to give 7.6 mg (1.4%) of portimine (**1**) and 2.9 mg (0.52%) of kabirimine (**2**).

Portimine (**1**): colorless plates, mp 244.5–246.3 °C (from MeOH-H_2_O); [α]_D_ −26 (*c* 0.6, MeOH); UV 231 nm (Δε 3.42, MeOH); FTIR (neat) 3388, 2954, 1714, 1636, 1456, 1360, 1141, 1062, 1048 cm^−1^. HRESIMS *m/z* 402.22760 [M + H]^+^ (calcd. for C_23_H_32_NO_5_ 402.22803).

Kabirimine (**2**): white powder, [α]_D_ +62 (*c* 0.08, MeOH); UV 230 nm (Δε 3.63, MeOH); FTIR (neat) 3419, 2969, 1671, 1540, 1456, 1201, 1132, 835, 800 cm^−1^; ^1^H and ^13^C NMR (DMSO-*d*_6_)—see Table 1. HRESIMS *m/z* 428.31763 [M + H]^+^ (calcd. for C_27_H_42_NO_3_ 428.31640).

### 3.4. Preparation of MPA Esters ***3*** and ***4*** of Portimine 

(*R*)-α-methoxyphenylacetic acid (MPA, 12.4 mg), DMAP (1.5 mg), and DCC (10.1 mg) were added to a solution of portimine (0.5 mg) in dry CH_2_Cl_2_ (500 µL). The reaction mixture was stirred at room temperature for three hours. After completion of the reaction, the solvent was removed with N_2_ flow, and the residue was purified by normal phase thin layer chromatography (TLC) developed with *n*-hexane-EtOAc (5:3) to afford 0.4 mg of *R*-MPA ester (**3**). Similarly, 0.4 mg of *S*-MPA ester (**4**) was prepared. 

*R*-MPA ester (**3**): ^1^H NMR (CD_3_OD) δ 5.98 (1H, dd, *J* = 17.4, 10.8 Hz, H-21), 5.62 (1H, brs, H-17), 5.13 (1H, brd, *J* = 11.0 Hz, H-15), 5.08 (1H, d, *J* = 17.4 Hz, H-22b), 4.96 (1H, d, *J* = 10.8 Hz, H-22a), 4.60 (1H, brs, H-5), 4.11 (1H, brd, *J* = 12.0 Hz, H-13), 3.99 (2H, m, H-10, H-1b), 3.71 (1H, dd, *J* = 15.5, 8.9 Hz, H-1a), 3.56 (1H, m, H-16), 3.37 (3H, s, OMe), 3.33 (3H, s, OMe), 2.78 (1H, brd, *J* = 16.0 Hz, H-6b), 2.33 (1H, m, H-19), 2.30 (1H, m, H-11), 2.25 (1H, m, H-12b), 1.79 (1H, m, H-6a), 0.81 (3H, d, *J* = 6.9 Hz, H-23).

*S*-MPA ester (**4**): ^1^H NMR (CD_3_OD) δ 6.13 (1H, dd, *J* = 17.4, 10.8 Hz, H-21), 5.50 (1H, brs, H-17), 5.18 (1H, brd, *J* = 11.0 Hz, H-15), 5.12 (1H, d, *J* = 17.4 Hz, H-22b), 4.98 (1H, d, *J* = 10.8 Hz, H-22a), 4.71 (1H, brs, H-5), 4.16 (1H, m, H-10), 4.13 (1H, brd, *J* = 12.0 Hz, H-13), 3.81 (1H, dd, *J* = 15.5, 8.9 Hz, H-1b), 3.64 (2H, m, H-1a, H-16), 3.52 (3H, s, OMe), 3.42 (3H, s, OMe), 2.94 (1H, brd, *J* = 16.0 Hz, H-6b), 2.35 (1H, m, H-19), 2.28 (1H, m, H-11), 2.20 (1H, m, H-12b), 1.93 (1H, m, H-6a), 0.81 (3H, d, *J* = 6.9 Hz, H-23); HRESIMS *m/z* 698.33061 [M+H]^+^ (calcd for C_41_H_47_NO_9_ 698.33291). 

### 3.5. Computational Study on the Isomers of Kabirimine (***2***)

The stable conformations of 16 candidate isomers were searched with the GRRM strategy at the HF/CEP-4G level. To reduce the computational cost, four methyl groups were replaced with iodine atoms. LADD = 4 [19], NoBondRearrage, and EQOnly options were also employed. More than 100 conformers for each isomer were obtained. After the methyl groups were restored to their original positions, the obtained geometries at the B3LYP/6-31G(d) level were refined, and the free energies at 300 K were calculated. Next, the optical rotation values were calculated with the TDDFT method at the B3LYP/aug-cc-pVDZ level [20]. The geometries and optical rotation values of the ten lowest free-energy conformations for each isomer are summarized in the Supplementary Materials. The optical rotation value of each isomer was obtained by thermally averaging those of all the obtained conformers. The GRRM and electronic structure calculations were performed using the GRRM14 [21] and Gaussian 09 [22] program packages at the Research Center for Computational Science, Okazaki, Japan.

### 3.6. Anti-RSV Assay 

The anti-RSV activities were evaluated by a cytopathic effects (CPE) assay based on previous reports [23,24,25]. In brief, HEp-2 (human epidermoid carcinoma of larynx) cells were seeded in 96-well plates at a density of 2.5 × 10^4^ cells/well in 100 µl of Eagle’s minimum essential medium (E-MEM) supplemented with 10% fetal bovine serum (FBS), and the cells were then incubated at 37 °C in an atmosphere of 5% CO_2_ overnight. After removing the medium, 100 µL of two-fold serially diluted samples from 50 µM to 0.097 µM were added. For the preparation of serial dilution series, samples were dissolved in DMSO (Wako pure chemical Corp.) and then diluted with serum-free E-MEM. Cells were subsequently infected with 100 µL of RSV (strain A2) in E-MEM supplemented with 4% FBS. The infectious dose was adjusted to 100 TCID_50_/well. At the same time, to validate the assay, DMSO and ribavirin (Sigma-Aldrich Co. LLC.) were evaluated as negative and positive control, respectively. After incubation at 37 °C in an atmosphere of 5% CO_2_ for three days, cells were fixed with 70% ethanol and then stained with 0.5% crystal violet. After washing with water and air drying, absorbance was measured at 560 nm using a microplate reader. The averaged absorbance of the uninfected groups was taken as 100%, and each measured value was converted to a relative value. Finally, a dose-response curve was represented for each tested compound (Figure S33). The 50% virus inhibitory concentration (IC_50_) of samples was calculated by statistical analysis (GraphPad Prism 5.0) based on the curve. All tests were performed in duplicate. 

### 3.7. X-ray Diffraction Analysis of Portimine (***1***) 

Platelet crystals of portimine (**1**) were obtained from a MeOH-H_2_O solution at room temperature. The X-ray diffraction study was conducted on a Rigaku R-AIXS RAPID II diffractometer with graphite monochromated Cu Kα radiationλ = 1.54187 Å. The structure was solved by direct methods and expanded using Fourier techniques. The non-hydrogen atoms were refined anisotropically. Hydrogen atoms were refined using the riding model. The final Flack parameter was 0.11(16), indicating that the absolute structure in Figure 2 is correct. Crystallographic data of compound **1** are provided in the Supplementary Materials.

## 4. Conclusions

From the extract of the cultured dinoflagellate *V*. *rugosum*, two cyclic imines, known as portimine (**1**) and new kabirimine (**2**), were isolated. The absolute configuration of the stereogenic carbons of **1** was determined in this study, and the structure of **2** was elucidated by a combination of spectroscopic analysis and calculation studies. Portimine (**1**) showed potent cytotoxicity, while kabirimine (**2**) showed moderate anti-RSV activity.

## Figures and Tables

**Table 1:** ^1^H and ^13^C NMR data for kabirimine (**2**) in dimethyl sulfoxide (DMSO)-d_6_.

Position	δ_C_	δ_H_ (*J* in Hz)	COSY	HMBC
1	42.1, CH_2_	3.77 brs (2H)	2	
2	24.7, CH_2_	2.14 m; 1.62 m	1; -	
3	25.3, CH	2.08 m	27	
4	48.1, C	-		
5	172.5, C	-		
6	29.0, CH_2_	3.13 brd (11.2); 2.92 m	7; -	
7	27.3, CH_2_	2.34 brt (11.2); 1.65 m	6; 8	
8	72.5, CH	3.93 m		
9	31.2, CH_2_	2.00 m; 1.43 brt (9.6)	8, 10; 8	
10	39.0, CH_2_	1.75 m; 1.63 m	9; -	25; 25
11	80.6, C	-		
12	43.9, CH_2_	2.10 brd (13.3); 1.26 brdd (13.3, 9.7)	13; -	13, 14; 13, 14
13	53.7, CH	2.89 brd (9.7)	12, 14	12
14	61.5, CH	2.66 brd (8.1)	13, 15	15
15	76.0, CH	2.96 brd (8.1)	14	14, 16, 26
16	36.0, CH	1.99 m	26	
17	33.3, CH_2_	1.98 m; 1.37 brt (12.5)	18; -	4; -
18	38.7, CH	3.06 brd (12.6)	17	
19	129.4, CH	5.70 brs		4, 18, 21, 23
20	134.8, C	-		
21	20.7, CH_2_	2.24 m (2H)	22	
22	35.6, CH_2_	2.07 m; 1.90 m	21; -	5; -
23	138.3, CH	6.42 dd (17.4, 10.9)	24	19, 21
24	112.1, CH_2_	5.13 d (17.4); 5.02 d (10.9)	23; 23	20; 20
25	23.7, CH_3_	1.19 s		10, 11, 12
26	10.5, CH_3_	1.05 brd (5.3)	16	15, 16, 17
27	17.7, CH_3_	0.96 d (6.6)	3	2, 3, 4

## Supplementary Materials

The following are available online at https://www.mdpi.com/1660-3397/17/6/353/s1. ^1^H and ^13^C NMR and other spectral data, crystallographic data, calculated geometries, and optical rotation values of kabirimine (**2**) are available.
