# Supplementary material for: Kabirimine, a New Cyclic Imine from an Okinawan Dinoflagellate
Source: Mar Drugs. 2019 Jun 13;17(6):353. doi: 10.3390/md17060353 (PMC6627061; doi:10.3390/md17060353)
Supplement: Supplementary file 1 [file marinedrugs-17-00353-s001.pdf]

Kabirimine, an antiviral cyclic imine from an Okinawan dinoflagellate

Idam Hermawan[1], Mikako Higa[1], Philipus Uli Basa Hutabarat[1], Takeshi Fujiwara[2], Kiyotaka Akiyama[2], Akihiko Kanamoto,[2] Takahiro Haruyama[3], Nobuyuki Kobayashi[3], Masahiro Higashi[4], Shoichiro Suda[5], and Junichi Tanaka[5,*]

[1] *Graduate School of Engineering and Science, University of the Ryukyus, Nishihara, Okinawa 903-0213, Japan*

[2] *OP Bio Factory Co., Ltd., Okinawa Life Science Center 107, 5-8 Suzaki, Uruma, Okinawa 904-2234, Japan*

[3] *Central Research Center, AVSS Corporation, Nagasaki 852-8137, Japan*

[4] *Department of Molecular Engineering, Kyoto University, Kyoto 615-8510, Japan*

[5] *Department of Chemistry, Biology and Marine Science, University of the Ryukyus, Nishihara, Okinawa 903-0213, Japan*

Email: jtanaka@sci.u-ryukyu.ac.jp

Table of Contents

Title	Page
Table S1. Figure S1. Calculated data and geometries for k01 isomer	3
Table S2. Figure S2. Calculated data and geometries for k02 isomer	4
Table S3. Figure S3. Calculated data and geometries for k03 isomer	5
Table S4. Figure S4. Calculated data and geometries for k04 isomer	6
Table S5. Figure S5. Calculated data and geometries for k05 isomer	7
Table S6. Figure S6. Calculated data and geometries for k06 isomer	8
Table S7. Figure S7. Calculated data and geometries for k07 isomer	9
Table S8. Figure S8. Calculated data and geometries for k08 isomer	10
Table S9. Figure S9. Calculated data and geometries for k09 isomer	11
Table S10. Figure S10. Calculated data and geometries for k10 isomer	12
Table S11. Figure S11. Calculated data and geometries for k11 isomer	13
Table S12. Figure S12. Calculated data and geometries for k12 isomer	14
Table S13. Figure S13. Calculated data and geometries for k13 isomer	15
Table S14. Figure S14. Calculated data and geometries for k14 isomer	16
Table S15. Figure S15. Calculated data and geometries for k15 isomer	17
Table S16. Figure S16. Calculated data for and geometries k16 isomer	18
Table S17. NMR data of portimine (**1**)	19
Figure S17. ^{1}H NMR spectrum of portimine (**1**)	20
Figure S18. ^{13}C NMR spectrum of portimine (**1**)	21
Figure S19. HRESIMS of portimine (**1**)	22
Figure S20. UV spectrum of portimine (**1**)	23
Figure S21. FTIR spectrum of portimine (**1**)	24
Figure S22. ^{1}H NMR spectrum of kabirimine (**2**)	25
Figure S23. ^{13}C NMR spectrum of kabirimine (**2**)	26
Figure S24. DEPT spectra of kabirimine (**2**)	27
Figure S25. COSY spectrum of kabirimine (**2**)	28
Figure S26. HSQC spectrum of kabirimine (**2**)	29
Figure S27. HMBC spectrum of kabirimine (**2**)	30
Figure S28. NOESY spectrum of kabirimine (**2**)	31
Figure S29. TOCSY spectrum of kabirimine (**2**)	32
Figure S30. HRESIMS spectrum of kabirimine (**2**)	33
Figure S31. UV spectrum of kabirimine (**2**)	34
Figure S32. FTIR spectrum of kabirimine (**2**)	35
Figure S33. Evaluation of anti-RSV activities and cytotoxicities of **1** and **2**	36
Figure S34. A stable conformation for each model structure A-D	37
X-ray structure report for portimine	38-67
Table S18. Atomic coordinates and B_{iso}/B_{eq}	47
Table S19. Atomic coordinates and B_{iso} involving hydrogen atoms	48
Table S20. Anisotropic displacement parameters	49
Table S21. Bond lengths	50
Table S22. Bond lengths involving hydrogens	51
Table S23. Bond angles	52
Table S24. Bond angles involving hydrogens	53
Table S25. Torsion angles	54-55
Table S26. Possible hydrogen bonds	56
Table S27. Intramolecular contacts less than 3.60 Å	57
Table S28. Intramolecular contacts less than 3.60 Å involving hydrogens	58-61
Table S29. Intermolecular contacts less than 3.60 Å	62
Table S30. Intermolecular contacts less than 3.60 Å involving hydrogens	63-67

Table S1. *Calculated dihedral angles,[1] free energies,[2] equilibrium populations, and optical rotation values of ten lowest free energy conformations of k01 isomer, (3S, 4R, 8R, 11R, 13R, 14R, 15R, 16R, 18S).*

Conf.	D1	D2	D3	D4	D5	D6	D7	D8	ΔG	P(%)	[α]D
**a**	179.5	38.9	4.4	−14.8	68.3	−32.6	−167.6	174.6	0.00	77.2	+129.3
**b**	177.0	41.5	15.7	24.8	162.9	−14.4	−70.1	−79.3	1.38	7.6	+5.7
**c**	177.1	39.5	13.0	25.7	66.3	−29.7	−168.9	167.7	1.62	5.1	−32.4
**d**	179.5	43.0	4.2	−14.6	67.8	21.0	−170.1	−178.1	1.69	4.6	+116.6
**e**	179.6	39.2	4.1	−23.4	168.8	−19.3	−73.0	−71.4	2.03	2.6	+106.2
**f**	−34.2	38.9	4.9	−15.2	68.2	−32.6	−167.6	175.0	2.76	0.8	+171.3
**g**	33.7	38.9	3.6	−14.5	68.5	−32.6	−167.6	174.2	2.86	0.6	−53.9
**h**	179.3	36.2	5.6	−12.3	149.8	0.9	−45.9	173.9	2.97	0.5	+42.3
**i**	179.5	38.1	3.5	−14.2	160.8	33.9	−163.8	156.4	3.26	0.3	+85.7
**j**	179.6	171.5	4.0	−14.9	67.9	−27.1	−169.5	−179.8	4.02	0.1	+101.4
									Average		+108.5

[1] D1: C19C20C23C24, D2: C14C15OH, D3: C19C20C21C22, D4: C4C5NC1, D5: C5C6C7C8, D6: C8C9C10C11, D7: C10C11C12C13, D8: C15C16C17C18. Units in degrees. [2]Units in kcal/mol.

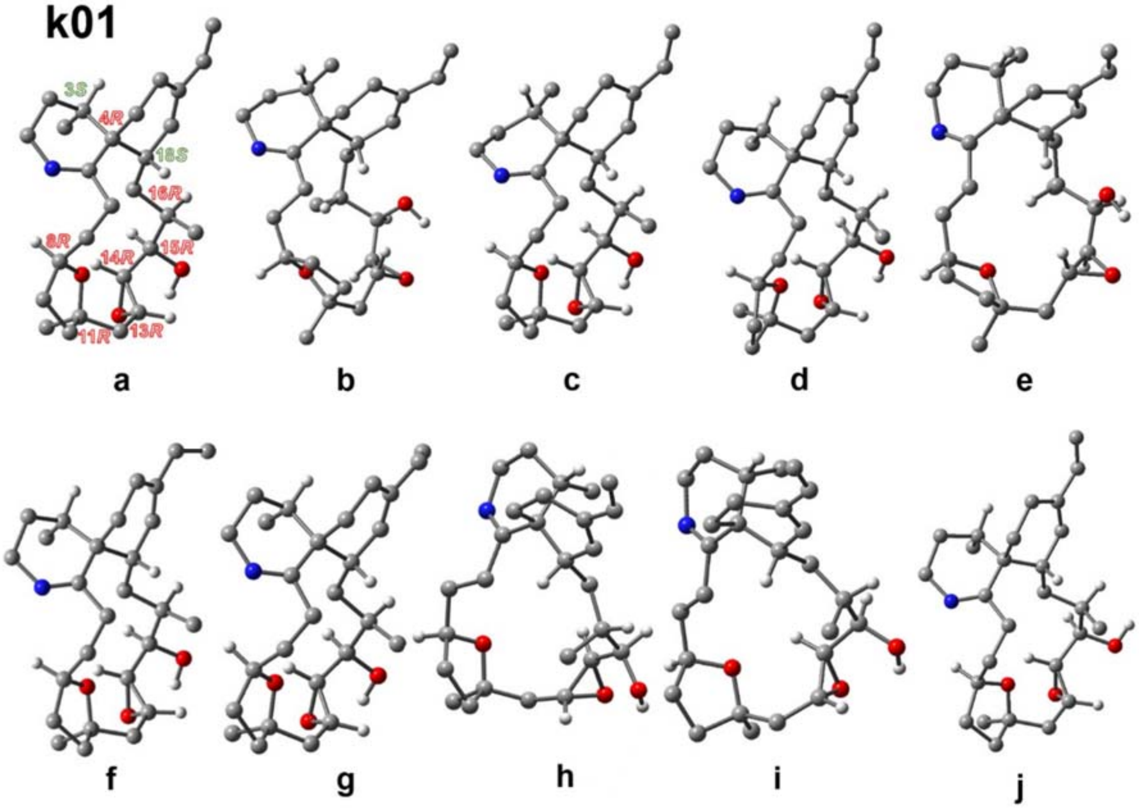

Figure S1. Calculated geometries of ten lowest free energy conformations of k01 isomer, (3*S*, 4*R*, 8*R*, 11*R*, 13*R*, 14*R*, 15*R*, 16*R*, 18*S*).

Table S2. Calculated dihedral angles,[1] free energies,[2] equilibrium populations, and optical rotation values of ten lowest free energy conformations of k02 isomer, (3*S*, 4*R*, 8*R*, 11*R*, 13*R*, 14*R*, 15*R*, 16*S*, 18*S*).

Conf.	D1	D2	D3	D4	D5	D6	D7	D8	ΔG	P(%)	[α]D
**a**	179.3	38.7	4.4	−12.6	70.0	−33.4	−167.1	168	0.00	73.7	+105.3
**b**	179.3	44.1	4.9	−11.9	67.6	31.2	−168.8	173.1	1.34	7.8	+78.0
**c**	179.3	40.1	4.5	−11.6	144.6	21.6	−45.8	174.7	1.75	3.9	−2.5
**d**	179.1	37.4	6.4	−8.5	59.4	−21.9	−79.0	−179.6	1.98	2.7	+100.7
**e**	179.4	35.5	5.8	−17.4	170.5	34.6	−166.5	134.0	2.11	2.2	+35.1
**f**	−178	38.4	−45.0	−18.2	70.5	−33.8	−167.2	168.4	2.56	1.0	+84.1
**g**	179.2	33.6	5.4	−10.4	61.2	27.1	−147.4	−175.7	2.59	1.0	+99.4
**h**	−34.7	38.6	5.0	−13.0	70.0	-33.5	−167.1	168.5	2.66	0.9	+141.1
**i**	177.1	37.3	13.9	19.5	−55.4	14.9	−71.1	−177.7	2.70	0.8	−88.6
**j**	−177.3	38.0	−14.7	−0.5	58.8	−35.1	−178.7	83.6	2.71	0.8	+202.8
									Average		+95.8

[1] D1: C19C20C23C24, D2: C14C15OH, D3: C19C20C21C22, D4: C4C5NC1, D5: C5C6C7C8, D6: C8C9C10C11, D7: C10C11C12C13, D8: C15C16C17C18. Units in degrees. [2]Units in kcal/mol.

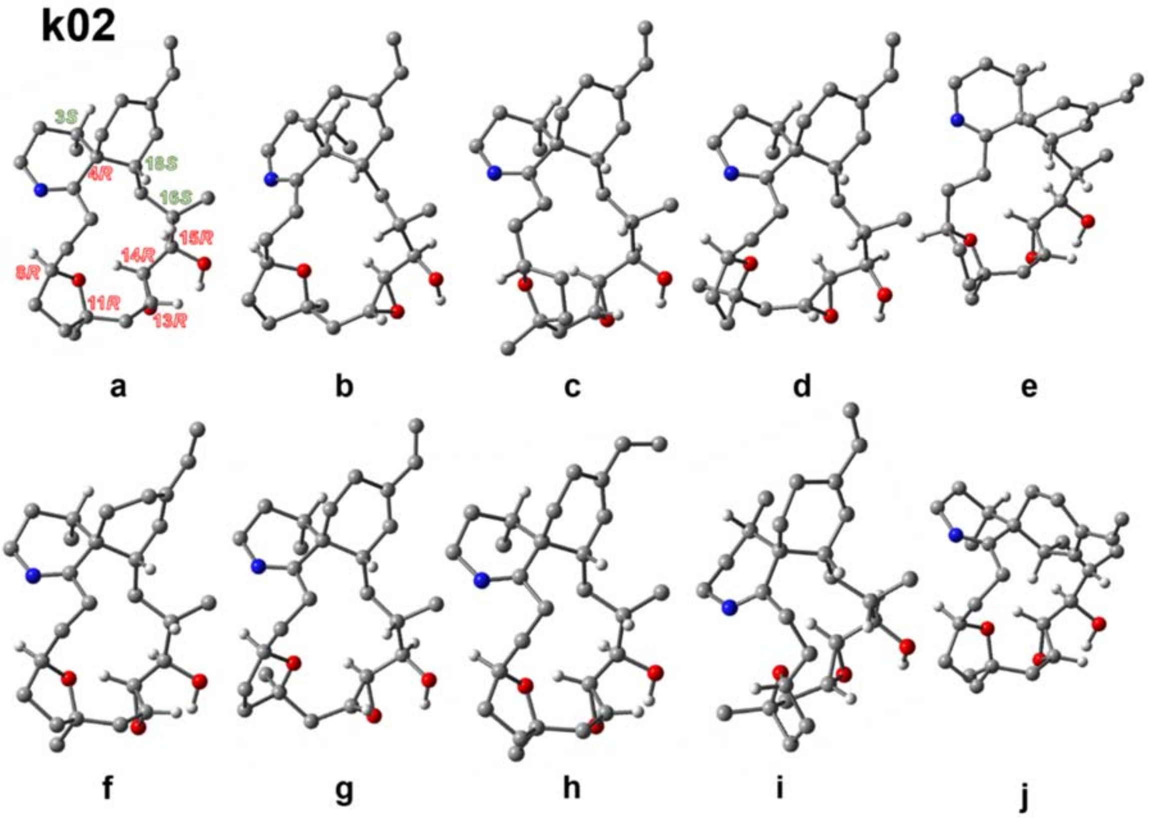

Figure S2. Calculated geometries of ten lowest free energy conformations of k02 isomer, (3*S*, 4*R*, 8*R*, 11*R*, 13*R*, 14*R*, 15*R*, 16*S*, 18*S*).

Table S3. Calculated dihedral angles,[1] free energies,[2] equilibrium populations, and optical rotation values of ten lowest free energy conformations of k03 isomer, (3*S*, 4*R*, 8*R*, 11*R*, 13*R*, 14*R*, 15*S*, 16*R*, 18*S*).

Conf.	D1	D2	D3	D4	D5	D6	D7	D8	ΔG	P(%)	[α]D
**a**	179.7	−51.6	3.9	−14.9	67.7	−28.8	−168.0	177.0	0.00	53.7	+137.6
**b**	177.4	−45	14.9	22.4	157.4	21.1	−65.1	−73.4	0.69	17.0	−140.2
**c**	179.7	−54.9	4.3	−21.9	171.4	−19	−73.2	−55.4	0.91	11.8	+92.1
**d**	179.4	37.2	3.2	−15.4	158.7	27.5	−48.2	69.0	1.40	5.2	+40.9
**e**	177.5	24.4	14.3	20.9	156	−15.4	−49.7	−68.1	1.58	3.8	−151.2
**f**	178.9	−53.5	8.5	−7.3	176.2	−26.0	−77.9	−49.1	2.31	1.1	+101.8
**g**	179.6	−176.4	3.8	−15.0	67.0	−31.7	−169.4	178.4	2.44	0.9	+173.0
**h**	−34.1	−51.7	4.5	−15.3	67.6	−28.8	−168	177.3	2.61	0.7	+168.8
**i**	33.5	−51.7	3.1	−14.7	67.9	−29.0	−168.0	176.4	2.68	0.6	−57.8
**j**	176.9	56.7	15.4	25.1	164.3	−15.4	−70.8	−65.9	2.83	0.5	+6.3
									Average		+59.9

[1] D1: C19C20C23C24, D2: C14C15OH, D3: C19C20C21C22, D4: C4C5NC1, D5: C5C6C7C8, D6: C8C9C10C11, D7: C10C11C12C13, D8: C15C16C17C18. Units in degrees. [2]Units in kcal/mol.

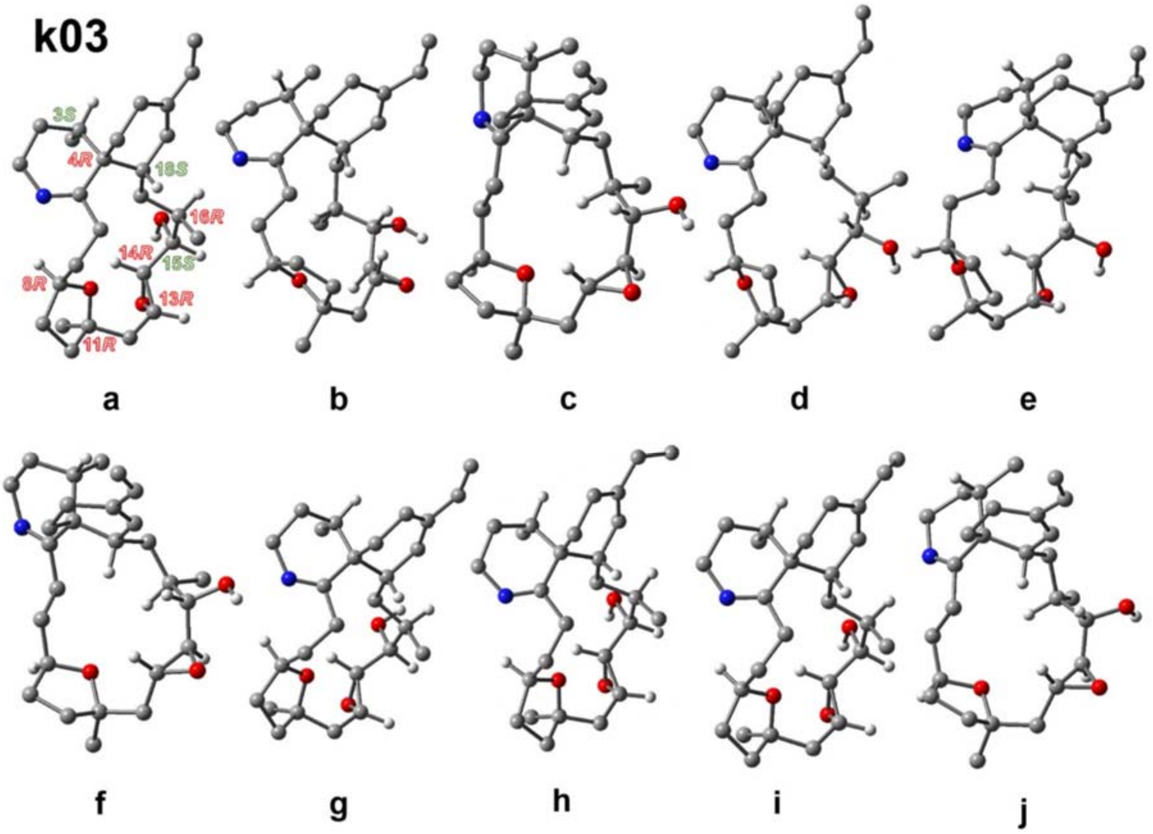

Figure S3. Calculated geometries of ten lowest free energy conformations of k03 isomer, (3*S*, 4*R*, 8*R*, 11*R*, 13*R*, 14*R*, 15*S*, 16*R*, 18*S*).

Table S4. Calculated dihedral angles,[1] free energies,[2] equilibrium populations, and optical rotation values of ten lowest free energy conformations of k04 isomer, (3*S*, 4*R*, 8*R*, 11*R*, 13*R*, 14*R*, 15*S*, 16*S*, 18*S*).

Conf.	D1	D2	D3	D4	D5	D6	D7	D8	ΔG	P(%)	[α]D
**a**	179.1	31.7	5.9	−13.2	163.6	25.5	−50.0	72.0	0.00	72.9	+80.7
**b**	179.4	−52.9	4.4	−12.6	70.0	−32.7	−165.8	167.6	1.29	8.3	+107.4
**c**	177.1	31.1	13.0	24.4	149.0	22.8	−49.2	65.9	1.56	5.3	−128.0
**d**	177.5	28.6	13.0	19.4	68.2	−32.1	−163.6	55.3	1.88	3.1	+15.1
**e**	179.4	−49.6	4.9	−12.7	68.7	29.3	−168.2	170.4	2.21	1.8	+80.0
**f**	179.0	27.1	7.0	−13.3	70.3	28.9	−164.4	72.8	2.63	0.9	+173.1
**g**	−178.1	30.7	−46.8	17.1	68.3	−30.4	−163.7	53.5	2.68	0.8	−61.0
**h**	−34.6	31.7	6.5	−13.9	163.6	25.3	−50.0	72.2	2.71	0.8	+162.1
**i**	179.1	-38.5	6.0	−11.7	67.5	−34.6	−85.4	85.1	2.76	0.7	+239.0
**j**	34.3	31.9	5.0	−12.9	163.5	25.3	−50.1	71.8	2.79	0.7	−63.5
									Average		+68.7

[1] D1: C19C20C23C24, D2: C14C15OH, D3: C19C20C21C22, D4: C4C5NC1, D5: C5C6C7C8, D6: C8C9C10C11, D7: C10C11C12C13, D8: C15C16C17C18. Units in degrees. [2]Units in kcal/mol.

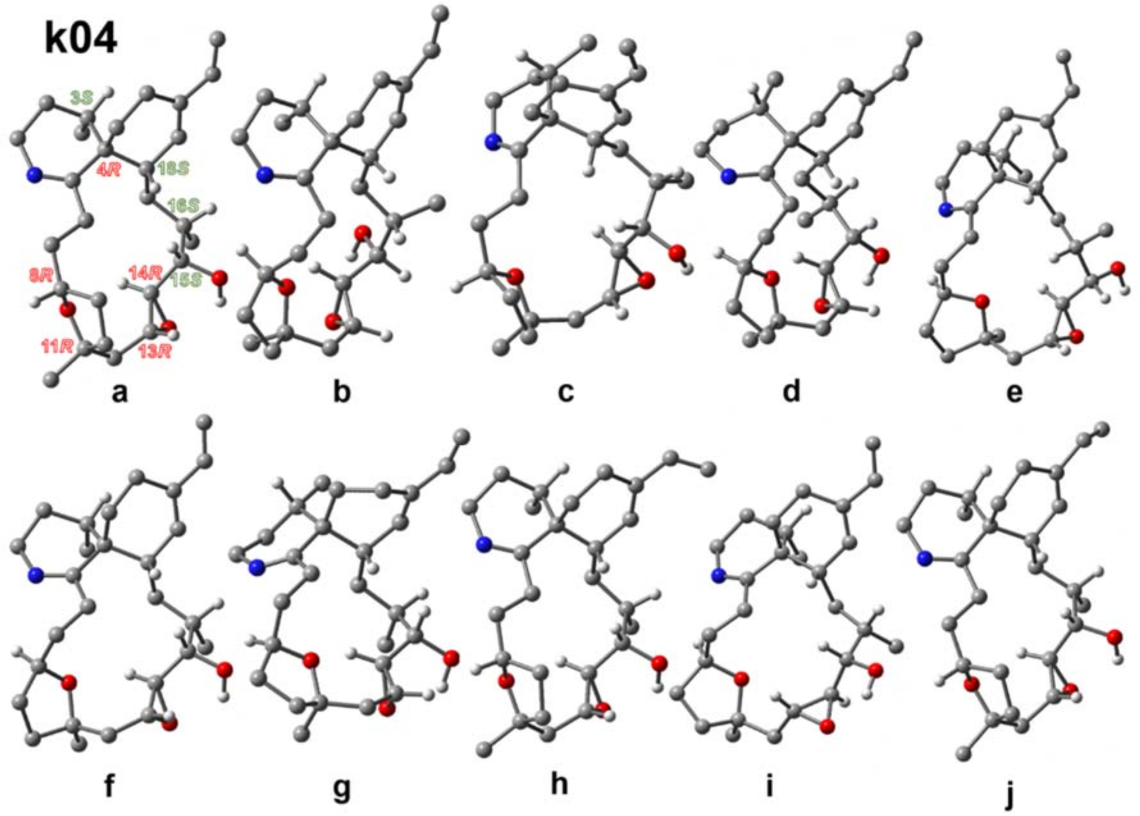

Figure S4. Calculated geometries of ten lowest free energy conformations of k04 isomer, (3*S*, 4*R*, 8*R*, 11*R*, 13*R*, 14*R*, 15*S*, 16*S*, 18*S*).

Table S5. Calculated dihedral angles,[1] free energies,[2] equilibrium populations, and optical rotation values of ten lowest free energy conformations of k05 isomer, (3*S*, 4*R*, 8*R*, 11*R*, 13*S*, 14*S*, 15*R*, 16*R*, 18*S*).

Conf.	D1	D2	D3	D4	D5	D6	D7	D8	ΔG	P(%)	$[\alpha]_D$
**a**	179.2	46.6	5.5	−11.8	59.7	17.3	−173.9	176.5	0.00	91.0	+212.9
**b**	177.4	48.9	13.2	19.3	−49.1	34.0	−159.8	175.7	2.38	1.7	−51.8
**c**	177.2	54.6	15.4	23.1	156.9	27.1	−164.1	−62.8	2.46	1.5	+7.8
**d**	177.0	46.4	13.2	27.1	59.1	13.7	−174.9	170.8	2.50	1.4	+46.1
**e**	−34.2	46.6	6.1	−12.1	59.7	17.3	−173.9	176.7	2.67	1.0	+229.8
**f**	33.8	46.5	4.7	−11.4	59.8	17.5	−173.8	176.2	2.78	0.9	+3.6
**g**	179.7	54.5	4.8	−24.3	164.9	29.9	−162.6	−57.4	3.08	0.5	+54.0
**h**	178.8	54.1	9.6	−7.1	−173.1	29.6	−170.3	−57.1	3.19	0.4	+71.6
**i**	179.3	176.6	5.3	−11.2	57.8	16.1	−171.5	−174.3	3.27	0.4	+202.3
**j**	178.9	55.4	8.2	−7.0	−164.5	−24.2	−163.5	−58.8	3.33	0.3	+112.9
									Average		+197.8

[1] D1: C19C20C23C24, D2: C14C15OH, D3: C19C20C21C22, D4: C4C5NC1, D5: C5C6C7C8, D6: C8C9C10C11, D7: C10C11C12C13, D8: C15C16C17C18. Units in degrees. [2]Units in kcal/mol.

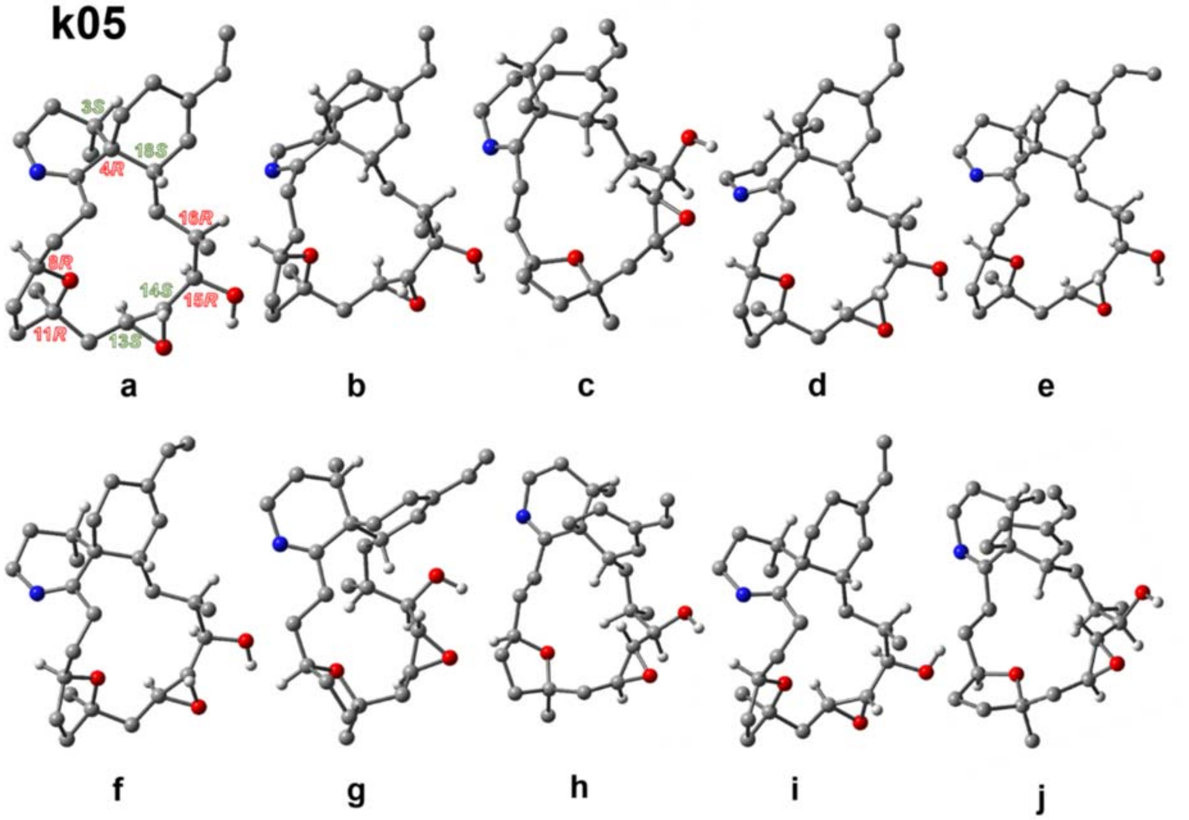

Figure S5. Calculated geometries of ten lowest free energy conformations of k05 isomer, (3*S*, 4*R*, 8*R*, 11*R*, 13*S*, 14*S*, 15*R*, 16*R*, 18*S*).

Table S6. Calculated dihedral angles,[1] free energies,[2] equilibrium populations, and optical rotation values of ten lowest free energy conformations of k06 isomer, (3*S*, 4*R*, 8*R*, 11*R*, 13*S*, 14*S*, 15*R*, 16*S*, 18*S*).

Conf.	D1	D2	D3	D4	D5	D6	D7	D8	ΔG	P(%)	[α]D
**a**	177.3	−28.3	12.9	20.8	65.5	−33.3	−175.0	121.5	0.00	28.8	+1.2
**b**	179.3	−32.4	4.9	−14.9	−179.3	34.2	−163.5	100.8	0.16	22.1	+86.3
**c**	177.4	−35.4	11.2	20.3	157.1	31.1	−164	91.8	0.52	12.1	−46.7
**d**	179.5	−22.5	5.5	−18.3	72.1	−34.1	−172.6	133.2	0.96	5.8	+144.1
**e**	176.8	51.2	12.9	23.6	62.4	−32.7	−172.9	53.5	1.20	3.9	+93.7
**f**	179.8	53.0	1.1	−19.9	68.7	−34.2	−171.2	57.6	1.33	3.1	+231.3
**g**	−177.4	42.4	−13.9	1.7	56.1	−28.2	−179.3	74.6	1.36	2.9	+198.8
**h**	−177.4	−26.3	−15.1	1.8	62.7	−27.0	178.5	64.0	1.39	2.8	+227.3
**i**	−178.0	−31.3	−47.2	17.6	65.6	−33.7	−175.3	112.4	1.44	2.6	−91.9
**j**	−177.0	48.9	−14.2	4.8	151.2	35.3	−167.3	59.5	1.46	2.5	+140.9
									Average		+51.28

[1] D1: C19C20C23C24, D2: C14C15OH, D3: C19C20C21C22, D4: C4C5NC1, D5: C5C6C7C8, D6: C8C9C10C11, D7: C10C11C12C13, D8: C15C16C17C18. Units in degrees. [2]Units in kcal/mol.

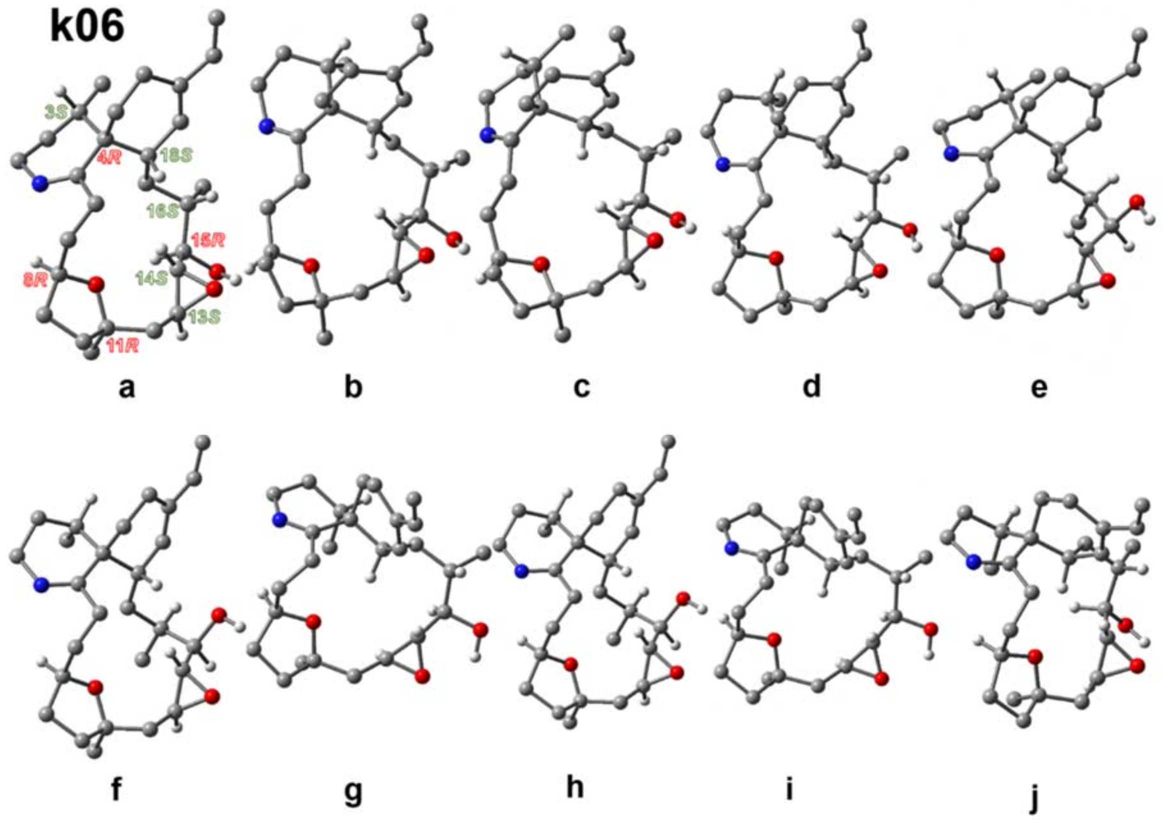

Figure S6. Calculated geometries of ten lowest free energy conformations of k06 isomer, (3*S*, 4*R*, 8*R*, 11*R*, 13*S*, 14*S*, 15*R*, 16*S*, 18*S*).

Table S7. Calculated dihedral angles,[1] free energies,[2] equilibrium populations, and optical rotation values of ten lowest free energy conformations of k07 isomer, (3*S*, 4*R*, 8*R*, 11*R*, 13*S*, 14*S*, 15*S*, 16*R*, 18*S*).

Conf.	D1	D2	D3	D4	D5	D6	D7	D8	ΔG	P(%)	[α]D
**a**	179.4	−43.1	5.2	−11.2	57.7	16.6	−170.3	−177.8	0.00	92.5	+236.7
**b**	177.5	−42.3	13.7	19.4	−48.8	33.4	−157.7	177.9	2.27	2.1	−34.5
**c**	177.1	−44.3	13.5	26.8	58.2	12.6	−171.9	174.2	2.74	0.9	+65.3
**d**	-34.1	−43.2	6.0	−11.5	57.6	16.8	−170.4	−177.5	2.85	0.8	+243.0
**e**	33.6	−43.2	4.6	−10.8	57.8	16.9	−170.3	−178.3	2.91	0.7	+13.8
**f**	177.1	−36.9	13.5	22.8	61.9	−31.0	−173.7	41.7	3.00	0.6	+70.0
**g**	−177.4	−36.5	−17.5	−1.5	59.6	−26.5	178.9	−67.0	3.38	0.3	+222.6
**h**	−179.9	−36.1	1.0	−21.2	68.3	−33.8	−171.6	46.2	3.42	0.3	+196.6
**i**	−178	−41.8	−46.6	16.1	−51.2	30.9	−158.2	175.4	3.59	0.2	−80.4
**j**	177.2	−37.9	14.1	22.0	154.3	26.1	−165.1	−58.7	3.60	0.2	+12.9
									Average		+223.8

[1] D1: C19C20C23C24, D2: C14C15OH, D3: C19C20C21C22, D4: C4C5NC1, D5: C5C6C7C8, D6: C8C9C10C11, D7: C10C11C12C13, D8: C15C16C17C18. Units in degrees. [2]Units in kcal/mol.

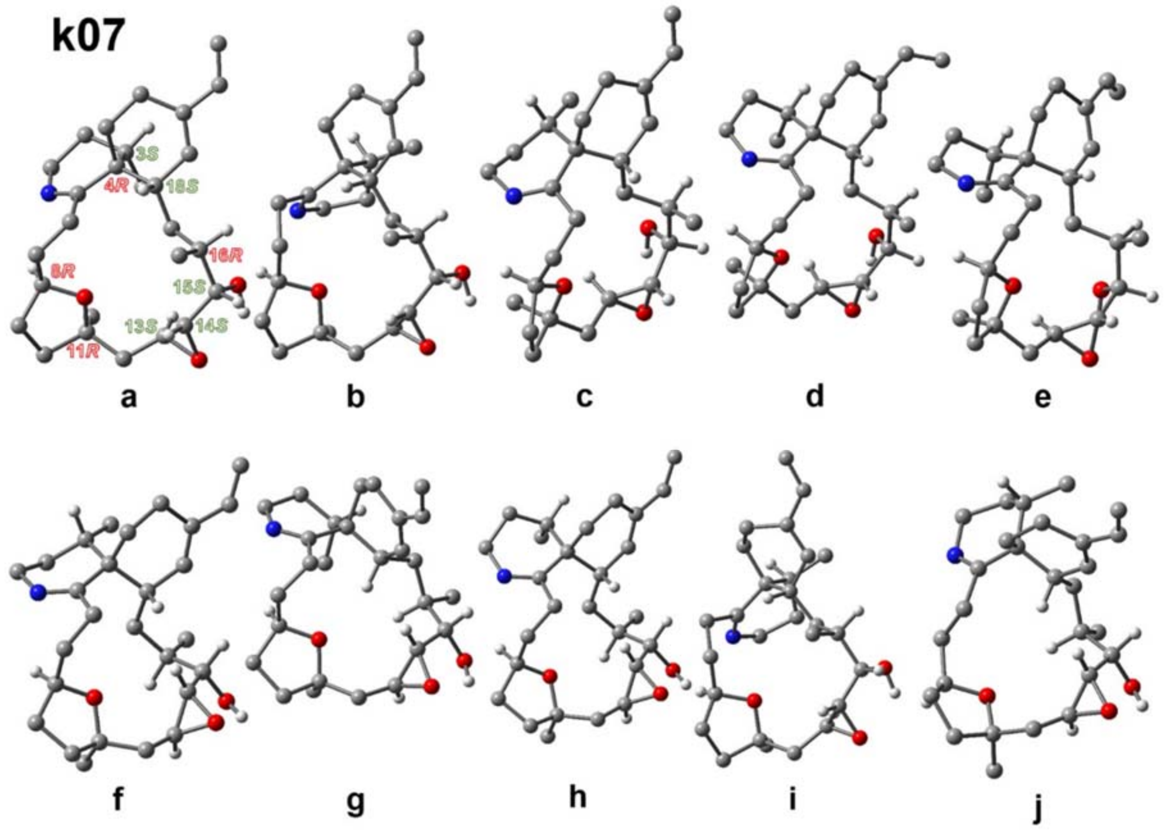

Figure S7. Calculated geometries of ten lowest free energy conformations of k07 isomer, (3*S*, 4*R*, 8*R*, 11*R*, 13*S*, 14*S*, 15*S*, 16*R*, 18*S*).

Table S8. Calculated dihedral angles,[1] free energies,[2] equilibrium populations, and optical rotation values of ten lowest free energy conformations of k08 isomer, (3*S*, 4*R*, 8*R*, 11*R*, 13*S*, 14*S*, 15*S*, 16*S*, 18*S*).

Conf.	D1	D2	D3	D4	D5	D6	D7	D8	ΔG	P(%)	$[\alpha]_D$
**a**	177.1	−35.0	12.1	22.9	62.3	−32.1	−170.8	47.4	0.00	38.4	+103.3
**b**	−179.9	−34.2	0.8	−20.3	69.3	−34.3	−169.4	52.9	0.16	29.3	+224.6
**c**	177.4	−23.8	11.9	19.8	161.6	30.6	−159.1	75.2	0.60	14.0	−39.3
**d**	179.1	−19.2	6.2	−14.0	−174.2	34.1	−160.3	85.2	0.64	13.0	+106.3
**e**	179.8	−28.4	2.2	−18.6	66.0	30.5	−172.1	67.5	2.02	1.3	+217.6
**f**	179.9	−37.9	0.1	−13.9	165.6	32.9	−164.2	37.8	2.15	1.1	+211.2
**g**	179.3	−39.4	5.0	−13.9	163.1	11.7	−55.7	48.5	2.68	0.4	+135.3
**h**	−34.1	−34.8	13.8	23.0	62.4	−32.2	−170.8	47.3	2.77	0.4	+149.5
**i**	−34.6	−34	1.3	−20.4	69.4	−34.3	−169.4	53.0	2.88	0.3	+278.4
**j**	179.1	46.6	5.9	−12.1	62.6	29.6	−157.1	83.0	3.05	0.2	+197.8
									Average		+121.8

[1] D1: C19C20C23C24, D2: C14C15OH, D3: C19C20C21C22, D4: C4C5NC1, D5: C5C6C7C8, D6: C8C9C10C11, D7: C10C11C12C13, D8: C15C16C17C18. Units in degrees. [2]Units in kcal/mol.

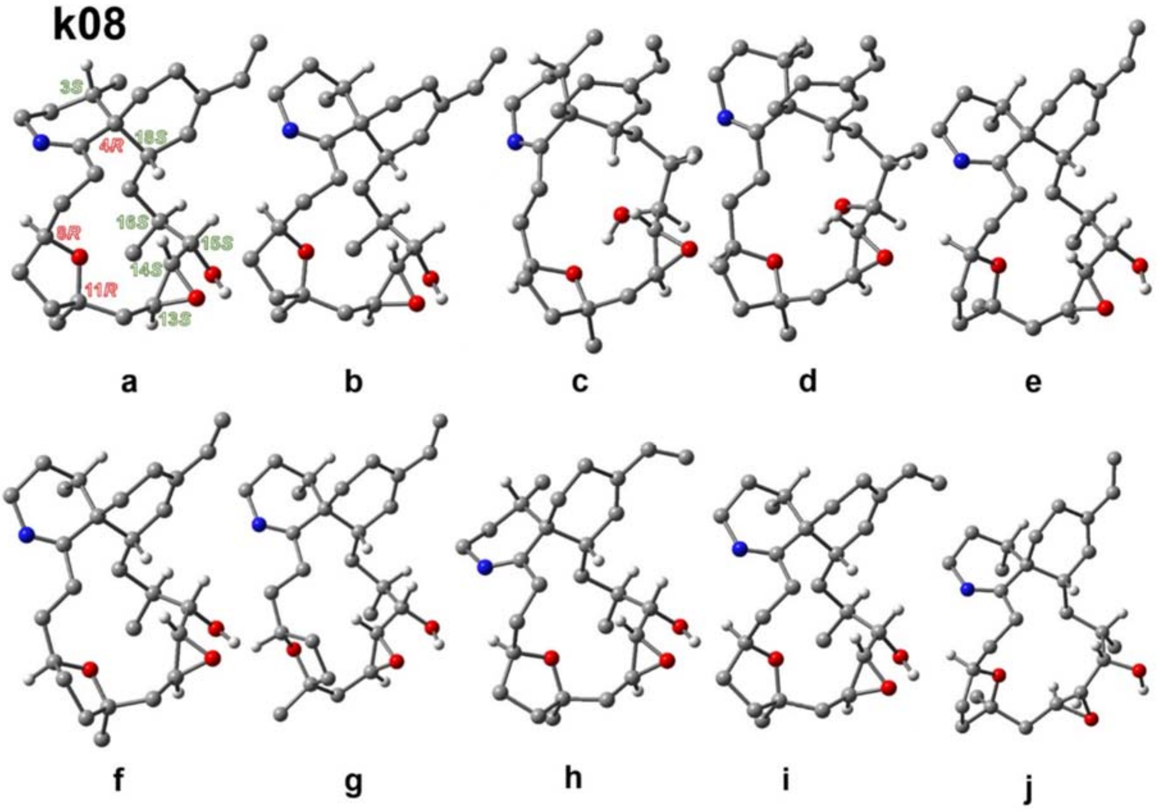

Figure S8. Calculated geometries of ten lowest free energy conformations of k08 isomer, (3*S*, 4*R*, 8*R*, 11*R*, 13*S*, 14*S*, 15*S*, 16*S*, 18*S*).

Table S9. Calculated dihedral angles,[1] free energies,[2] equilibrium populations, and optical rotation values of ten lowest free energy conformations of k09 isomer, (3*S*, 4*R*, 8*S*, 11*S*, 13*R*, 14*R*, 15*R*, 16*R*, 18*S*).

Conf.	D1	D2	D3	D4	D5	D6	D7	D8	ΔG	P(%)	[α]D
**a**	179.8	39.5	1.8	−15.6	165.9	−32.7	170.2	175.8	0.00	75.6	+121.7
**b**	179.5	38.5	3.5	−14.3	156.4	20.2	166.3	−179.4	1.01	14.0	+81.8
**c**	177.1	39.4	11.4	24.0	152.8	−33.1	172.9	164.6	1.66	4.7	−10.9
**d**	177.1	37.7	12.2	23.8	135.1	15.1	171.5	173.7	1.96	2.8	−49.3
**e**	-34.4	39.4	2.1	−15.9	165.7	−32.7	170.1	176.1	2.61	1.0	+197.5
**f**	33.3	39.5	0.9	−15.4	165.9	−32.6	170.2	175.7	2.77	0.7	−35.7
**g**	−34.2	38.5	4.0	−14.7	155.9	20.0	166.4	−179.0	3.59	0.2	+140.9
**h**	33.6	38.5	2.8	−14.0	156.6	20.7	166.2	−179.7	3.65	0.2	−87.6
**i**	177.1	30.1	13.0	21.9	161.1	33.6	161.7	71.4	3.85	0.1	−95.5
**j**	179.9	20.3	0.9	−20.0	179.9	−32.3	167.9	76.2	3.90	0.1	+93.9
									Average		+103.0

[1] D1: C19C20C23C24, D2: C14C15OH, D3: C19C20C21C22, D4: C4C5NC1, D5: C5C6C7C8, D6: C8C9C10C11, D7: C10C11C12C13, D8: C15C16C17C18. Units in degrees. [2]Units in kcal/mol.

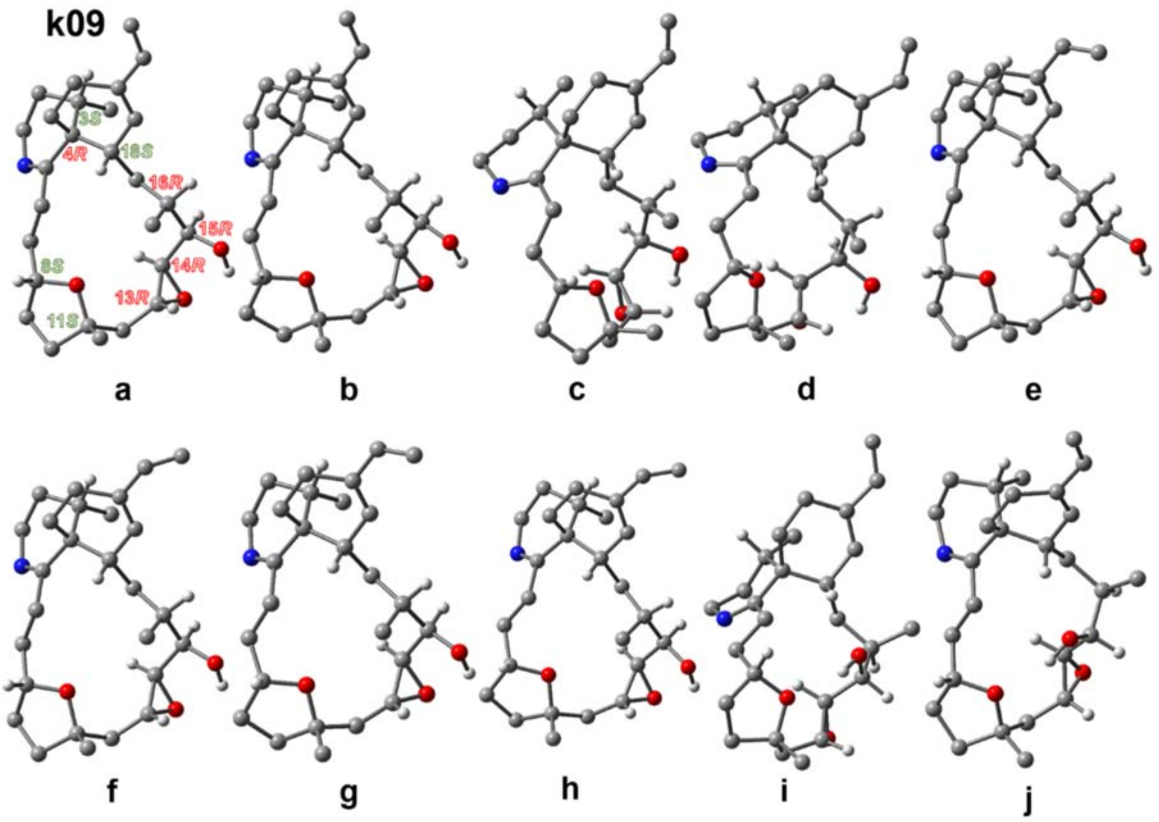

Figure S9. Calculated geometries of ten lowest free energy conformations of k09 isomer, (3*S*, 4*R*, 8*S*, 11*S*, 13*R*, 14*R*, 15*R*, 16*R*, 18*S*).

Table S10. Calculated dihedral angles,[1] free energies,[2] equilibrium populations, and optical rotation values of ten lowest free energy conformations of k10 isomer, (3*S*, 4*R*, 8*S*, 11*S*, 13*R*, 14*R*, 15*R*, 16*S*, 18*S*).

Conf.	D1	D2	D3	D4	D5	D6	D7	D8	ΔG	P(%)	$[\alpha]_D$
**a**	179.7	42.5	1.8	−15.2	171.7	−33.1	170.7	171.0	0.00	47.0	+84.6
**b**	179.5	41.1	3.8	−14.0	169.8	33.2	162.8	172.7	0.37	25.3	+16.9
**c**	179.8	13.5	1.8	−18.0	−179.7	−33.5	166.6	82.9	1.03	8.4	+109.9
**d**	−38.5	11.6	4.6	−16.9	−175.5	−147.4	-88.9	116.1	1.33	5.0	+52.8
**e**	177.2	16.7	11.5	21.9	167.3	−32.7	170.1	72.5	1.46	4.1	−53.4
**f**	177.1	26.1	12.7	22.3	162.5	33.9	160.8	76.1	1.52	3.7	−97.9
**g**	177.1	41.4	11.3	23.5	158.0	−34.1	173.8	159.7	2.09	1.4	−38.7
**h**	176.9	40.8	13.4	27.5	161.5	33.4	165.6	163.2	2.60	0.6	−118.8
**i**	−34.6	42.5	2.3	−15.6	171.7	−33.2	170.6	171.3	2.65	0.6	+158.0
**j**	177.6	41.0	13.9	20.3	150.9	−35.8	174.0	142.3	2.67	0.5	−64.4
									Average		+49.13

[1] D1: C19C20C23C24, D2: C14C15OH, D3: C19C20C21C22, D4: C4C5NC1, D5: C5C6C7C8, D6: C8C9C10C11, D7: C10C11C12C13, D8: C15C16C17C18. Units in degrees. [2]Units in kcal/mol.

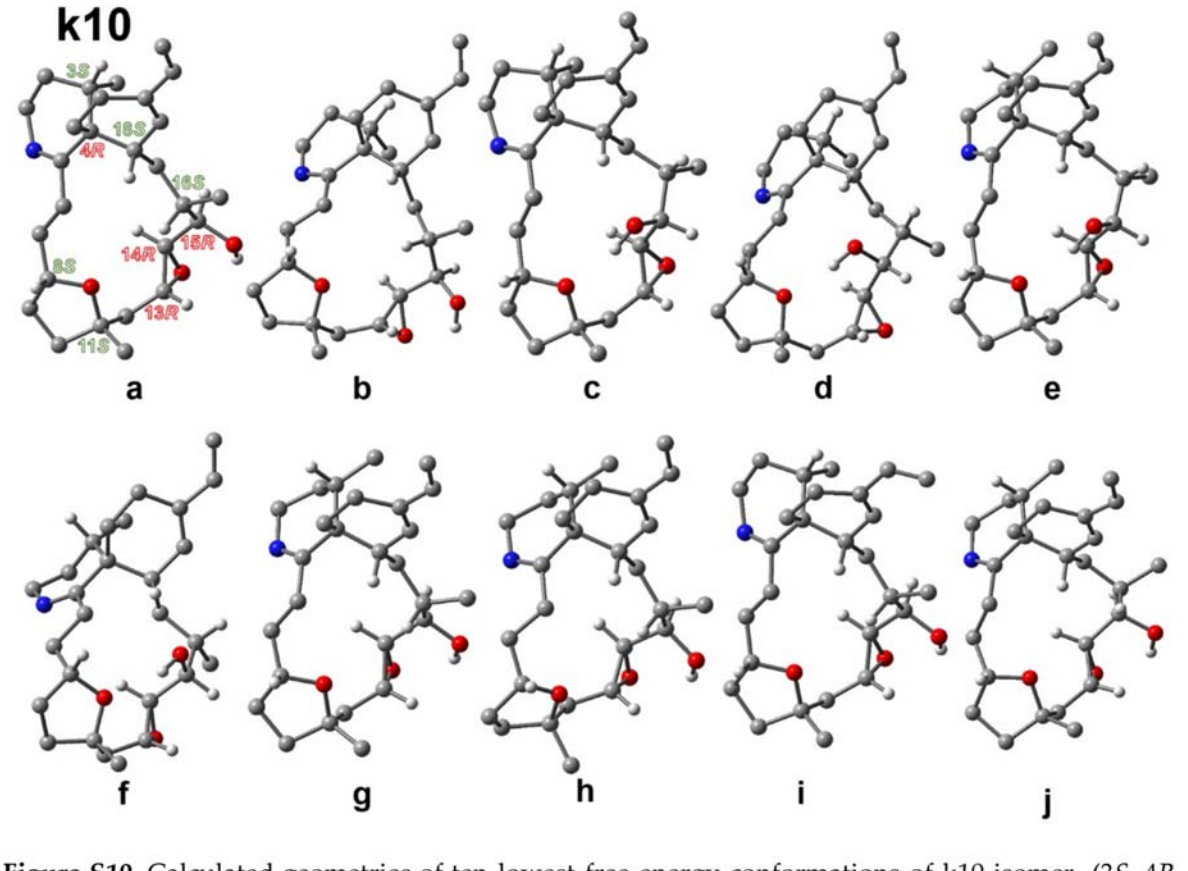

Figure S10. Calculated geometries of ten lowest free energy conformations of k10 isomer, (3*S*, 4*R*, 8*S*, 11*S*, 13*R*, 14*R*, 15*R*, 16*S*, 18*S*).

Table S11. Calculated dihedral angles,[1] free energies,[2] equilibrium populations, and optical rotation values of ten lowest free energy conformations of k11 isomer, (3*S*, 4*R*, 8*S*, 11*S*, 13*R*, 14*R*, 15*S*, 16*R*, 18*S*).

Conf.	D1	D2	D3	D4	D5	D6	D7	D8	ΔG	P(%)	[α]D
**a**	179.9	−51.4	1.8	−15.2	166.1	−33.0	171.6	174.8	0.00	63.5	+141.7
**b**	179.7	−51.8	3.5	−14.1	156.8	24.9	166.8	179.6	0.93	13.4	+95.3
**c**	177.6	28.0	13.4	18.8	168.6	−34.5	157.9	−68.8	1.17	8.9	−29.5
**d**	177.2	−51.9	11.6	24.3	153.7	−33.5	174.1	163.3	1.85	2.8	+10.6
**e**	177.5	40.8	11.4	19.5	168.5	−32.9	172.5	52.7	1.98	2.3	−22.8
**f**	177.2	−52.5	12.4	25.2	140.4	22.7	170.2	172.9	2.25	1.4	−47.7
**g**	179.9	−174.3	1.4	−15.5	165.4	−33.0	171.6	178.8	2.58	0.8	+169.6
**h**	−34.2	−51.5	2.2	−15.6	166.0	−33.1	171.5	175.0	2.61	0.8	+212.6
**i**	33.2	−51.4	0.9	−15.1	166.1	−33.0	171.6	174.4	2.71	0.7	−26.2
**j**	−179.9	40.0	−0.1	−21.0	−176.0	−32.8	167.3	64.5	2.72	0.7	+118.8
									Average		+101.6

[1] D1: C19C20C23C24, D2: C14C15OH, D3: C19C20C21C22, D4: C4C5NC1, D5: C5C6C7C8, D6: C8C9C10C11, D7: C10C11C12C13, D8: C15C16C17C18. Units in degrees. [2]Units in kcal/mol.

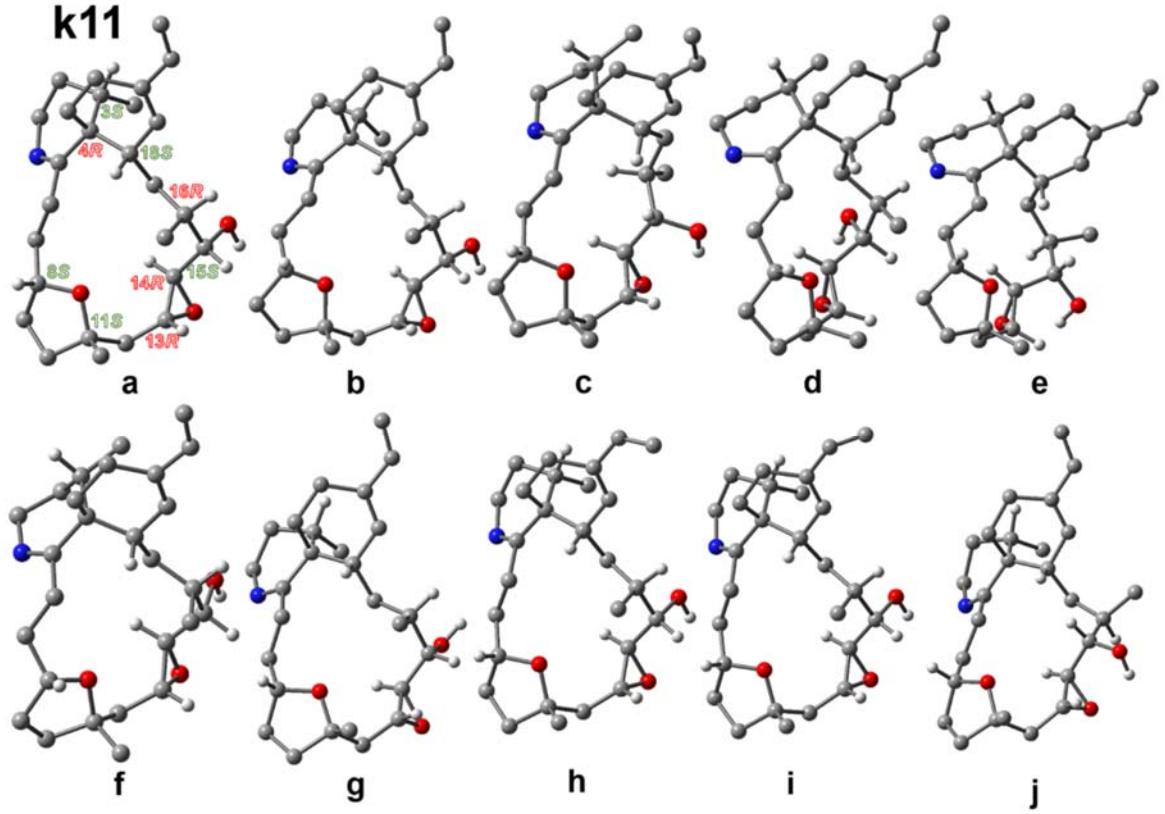

Figure S11. Calculated geometries of ten lowest free energy conformations of k11 isomer, (3*S*, 4*R*, 8*S*, 11*S*, 13*R*, 14*R*, 15*S*, 16*R*, 18*S*).

Table S12. Calculated dihedral angles,[1] free energies,[2] equilibrium populations, and optical rotation values of ten lowest free energy conformations of k12 isomer, (3*S*, 4*R*, 8*S*, 11*S*, 13*R*, 14*R*, 15*S*, 16*S*, 18*S*).

Conf.	D1	D2	D3	D4	D5	D6	D7	D8	ΔG	P(%)	[α]D
**a**	177.5	34.7	11.4	20.3	167.5	−34.0	169.6	55.4	0.00	42.1	+10.7
**b**	179.9	33.7	1.1	−19.8	−177.5	−33.6	165.0	68.5	0.54	16.9	+151.5
**c**	179.8	−50.6	1.9	−15.0	172.0	−33.2	171.9	168.0	0.57	16.2	+96.1
**d**	179.5	−51.4	4.0	−13.6	169.3	32.5	163.2	169.1	0.98	8.1	+33.4
**e**	177.3	33.1	12.2	21.7	168.0	32.0	158.0	64.0	1.18	5.8	−63.2
**f**	−178.3	35.6	−47.1	18.3	164.6	−33.8	170.9	54.2	1.44	3.8	−54.9
**g**	179.4	32.1	4.2	−17.0	−179.5	30.6	154.5	73.3	1.46	3.6	+94.1
**h**	177.0	−50.2	11.9	24.1	160.8	−33.7	175.0	153.7	2.57	0.6	−33.6
**i**	−178.1	33.9	−47.1	18.6	164.3	32.5	159.8	62.0	2.67	0.5	−115.0
**j**	33.9	34.9	10.6	20.3	167.3	−34.0	169.8	55.1	2.73	0.4	−113.3
									Average		+45.5

[1] D1: C19C20C23C24, D2: C14C15OH, D3: C19C20C21C22, D4: C4C5NC1, D5: C5C6C7C8, D6: C8C9C10C11, D7: C10C11C12C13, D8: C15C16C17C18. Units in degrees. [2]Units in kcal/mol.

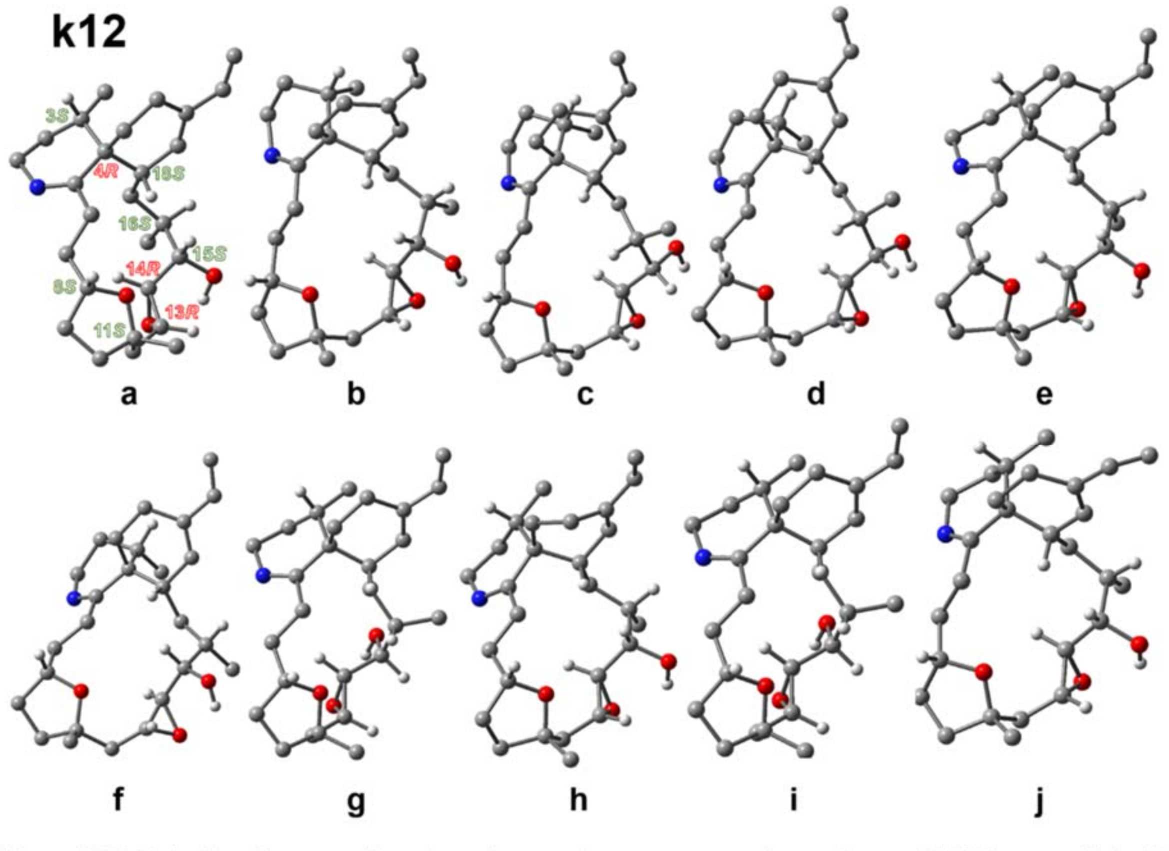

Figure S12. Calculated geometries of ten lowest free energy conformations of k12 isomer, (3*S*, 4*R*, 8*S*, 11*S*, 13*R*, 14*R*, 15*S*, 16*S*, 18*S*).

Table S13. Calculated dihedral angles,[1] free energies,[2] equilibrium populations, and optical rotation values of ten lowest free energy conformations of k13 isomer, (3*S*, 4*R*, 8*S*, 11*S*, 13*S*, 14*S*, 15*R*, 16*R*, 18*S*).

Conf.	D1	D2	D3	D4	D5	D6	D7	D8	ΔG	P(%)	[α]D
**a**	177.3	54.1	15.5	22.5	164.9	14.1	54.3	−62.7	0.00	25.6	+27.4
**b**	179.5	47.6	4.4	−14.2	158.4	32.9	171.0	165.1	0.13	20.5	+137.9
**c**	177.3	54.9	14.9	22.1	161.5	−17.2	59.9	−61.3	0.21	17.9	+41.1
**d**	177.6	−35.4	11.0	19.0	176.5	29.5	49.6	76.8	0.72	7.7	−64.0
**e**	−180.0	51.2	0.4	−15.6	170.2	28.0	76.6	170.2	1.25	3.2	+134.6
**f**	−178.3	−34.9	−47.1	16.4	173.1	28.9	49.1	76.5	1.36	2.6	−123.1
**g**	179.0	54.4	9.0	−29.7	164.5	18.2	54.0	−61.6	1.39	2.5	+38.7
**h**	177.6	46.4	13.5	19.5	173.4	29.4	50.1	−94.3	1.41	2.4	−27.9
**i**	179.8	42.6	1.7	−14.7	168.9	−30.2	171.8	164.8	1.42	2.4	+160.5
**j**	177.7	47.9	13.9	19.3	161.9	−19.9	63.8	−97.5	1.47	2.2	+23.6
									Average		+51.7

[1] D1: C19C20C23C24, D2: C14C15OH, D3: C19C20C21C22, D4: C4C5NC1, D5: C5C6C7C8, D6: C8C9C10C11, D7: C10C11C12C13, D8: C15C16C17C18. Units in degrees. [2]Units in kcal/mol.

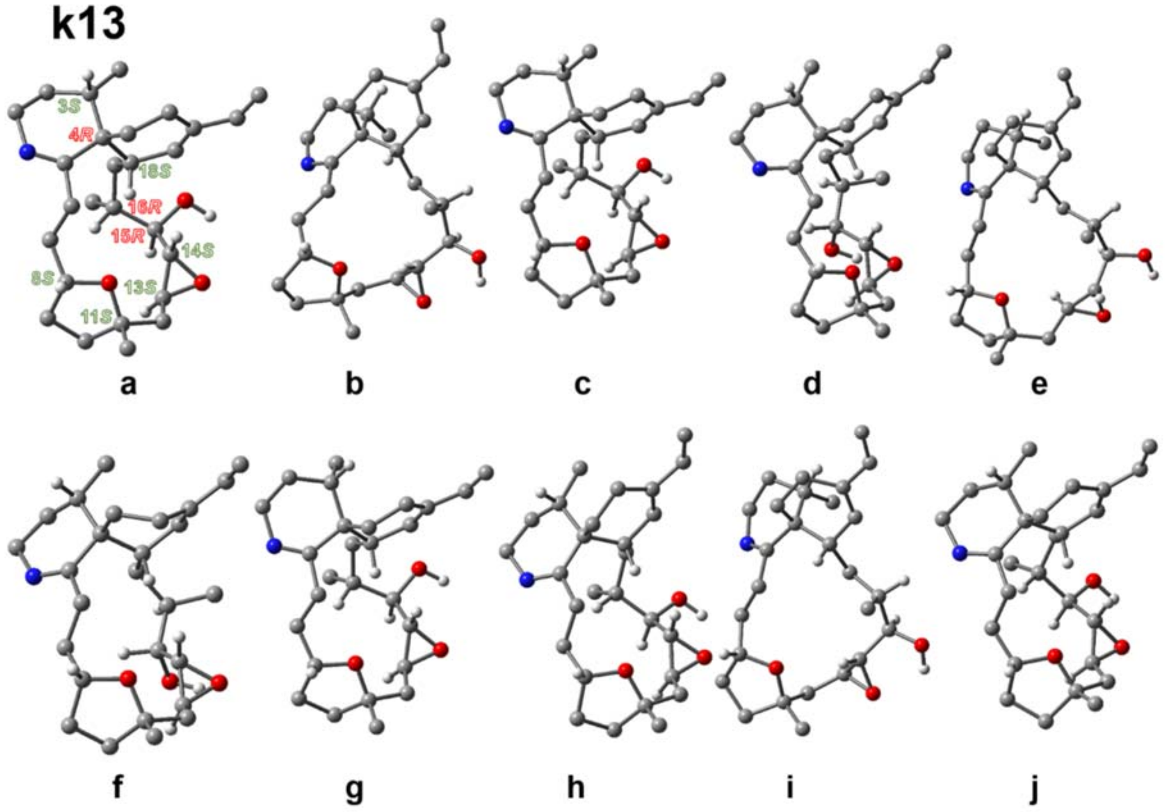

Figure S13. Calculated geometries of ten lowest free energy conformations of k13 isomer, (3*S*, 4*R*, 8*S*, 11*S*, 13*S*, 14*S*, 15*R*, 16*R*, 18*S*).

Table S14. Calculated dihedral angles,[1] free energies,[2] equilibrium populations, and optical rotation values of ten lowest free energy conformations of k14 isomer, (3*S*, 4*R*, 8*S*, 11*S*, 13*S*, 14*S*, 15*R*, 16*S*, 18*S*).

Conf.	D1	D2	D3	D4	D5	D6	D7	D8	ΔG	P(%)	[α]D
**a**	177.5	−37.0	11.5	20.0	161.4	−35.1	164.6	119.1	0.00	30.3	−37.7
**b**	179.7	51.6	2.9	−15.5	178.6	27.7	77.8	163.5	0.42	14.9	+71.3
**c**	177.7	−37.8	11.0	19.9	175.2	28.3	46.4	86.4	0.48	13.5	−23.6
**d**	177.7	−33.6	12.0	19.9	171.0	−25.7	58.6	78.9	0.60	11.2	+26.3
**e**	177.4	−40.5	14.6	20.2	174.2	28.9	67.9	81.4	0.99	5.7	−45.8
**f**	179.7	50.7	2.1	−15.2	−178.2	−27.3	88.6	166.6	1.28	3.6	+139.9
**g**	179.6	49.1	2.8	−15.0	159.7	31.9	170.5	161.2	1.28	3.6	+120.3
**h**	179.7	54.8	3.0	−16.5	170.1	−36.3	130.8	168.0	1.30	3.4	+123.4
**i**	179.9	−34.2	2.7	−21.8	175.2	−35.2	162.4	132.3	1.32	3.3	+65.4
**j**	−178.0	-31.3	−46.2	16.6	165.5	−26.5	59.2	73.1	1.44	2.7	−47.6
									Average		+15.8

[1] D1: C19C20C23C24, D2: C14C15OH, D3: C19C20C21C22, D4: C4C5NC1, D5: C5C6C7C8, D6: C8C9C10C11, D7: C10C11C12C13, D8: C15C16C17C18. Units in degrees. [2]Units in kcal/mol.

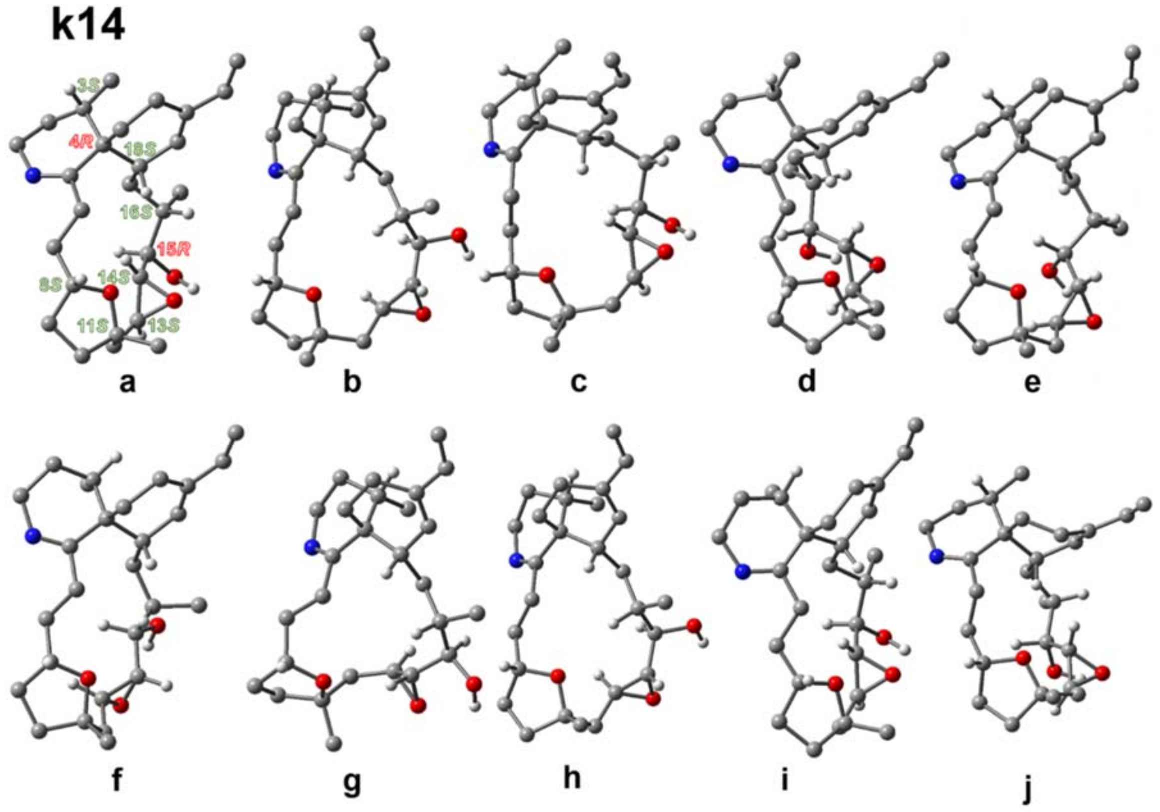

Figure S14. Calculated geometries of ten lowest free energy conformations of k14 isomer, (3*S*, 4*R*, 8*S*, 11*S*, 13*S*, 14*S*, 15*R*, 16*S*, 18*S*).

Table S15. Calculated dihedral angles,[1] free energies,[2] equilibrium populations, and optical rotation values of ten lowest free energy conformations of k15 isomer, (3*S*, 4*R*, 8*S*, 11*S*, 13*S*, 14*S*, 15*S*, 16*R*, 18*S*).

Conf.	D1	D2	D3	D4	D5	D6	D7	D8	ΔG	P(%)	[α]D
**a**	179.6	−45.9	4.1	−14.5	155.4	33.0	171.3	166.9	0.00	41.2	+161.0
**b**	−179.9	−31.2	0.4	−17.3	156.2	−35.3	146.3	179.3	0.53	16.8	+203.7
**c**	177.2	−37.0	13.9	21.6	160.1	−17.5	59.6	−60.3	0.67	13.5	+45.4
**d**	177.2	55.0	14.6	22.5	175.3	34.1	82.2	−72.5	1.29	4.7	−53.2
**e**	−178.4	−35.1	−47.6	16.9	153.7	−19.5	59.7	−57.7	1.31	4.6	−33.1
**f**	179.7	−35.9	3.5	−24.5	173.1	21.9	55.6	−59.0	1.38	4.0	+105.0
**g**	177.2	−38.4	12.4	22.5	167.9	−33.6	168.3	38.2	1.74	2.2	+40.1
**h**	−179.8	−37.4	−0.3	−21.3	175.7	−34.0	166.1	45.8	1.75	2.2	+168.5
**i**	177.1	−30.0	11.8	23.8	138.2	−33.2	150.1	171.9	1.92	1.6	+66.0
**j**	177.4	58.7	14.3	20.9	167.3	−36.6	131.7	−76.6	2.04	1.4	−3.3
									Average		+116.7

[1] D1: C19C20C23C24, D2: C14C15OH, D3: C19C20C21C22, D4: C4C5NC1, D5: C5C6C7C8, D6: C8C9C10C11, D7: C10C11C12C13, D8: C15C16C17C18. Units in degrees. [2]Units in kcal/mol.

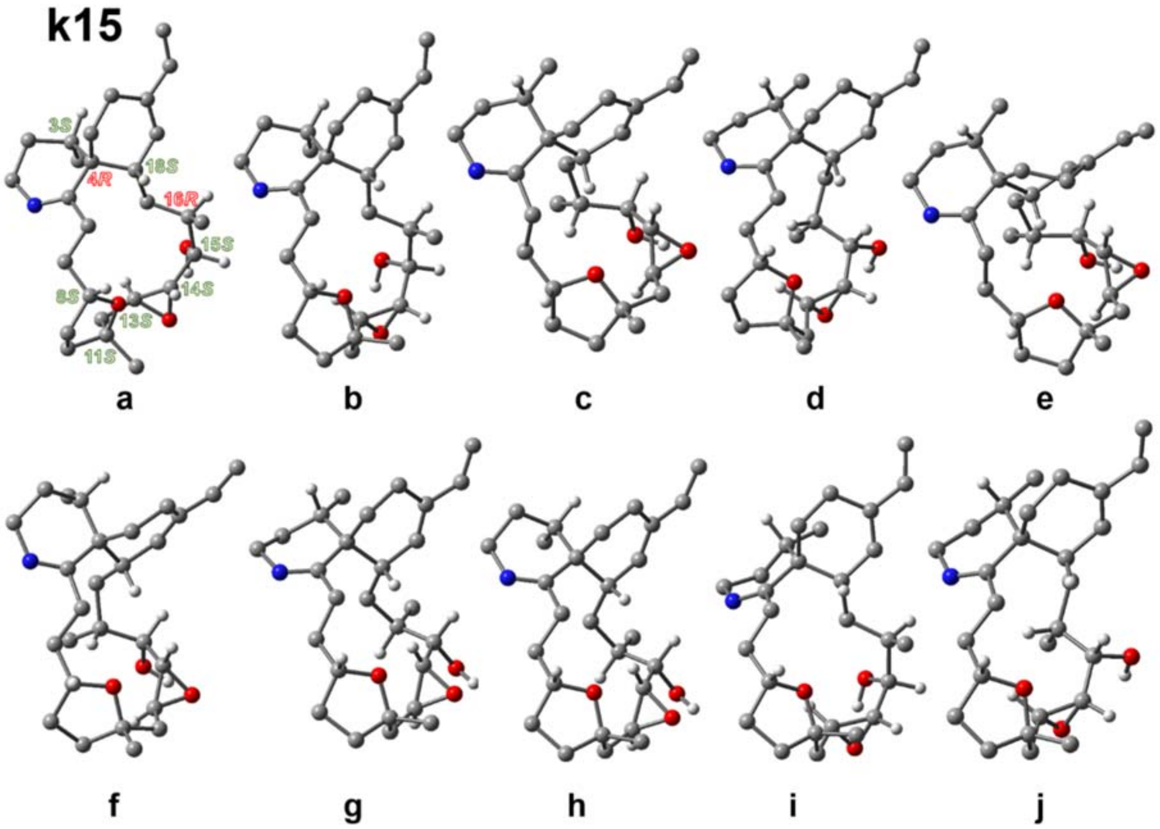

Figure S15. Calculated geometries of ten lowest free energy conformations of k15 isomer, (3*S*, 4*R*, 8*S*, 11*S*, 13*S*, 14*S*, 15*S*, 16*R*, 18*S*).

Table S16. Calculated dihedral angles,[1] free energies,[2] equilibrium populations, and optical rotation values of ten lowest free energy conformations of k16 isomer, (3*S*, 4*R*, 8*S*, 11*S*, 13*S*, 14*S*, 15*S*, 16*S*, 18*S*).

Conf.	D1	D2	D3	D4	D5	D6	D7	D8	ΔG	P(%)	[α]D
**a**	179.7	−42.2	3.2	−16.4	178.9	29.6	75.7	168.6	0.00	39.4	+65.8
**b**	179.7	−44.6	2.7	−15.0	157.0	31.8	169.9	162.3	0.26	25.4	+131.2
**c**	179.7	-37.7	3.3	−17.8	169.7	−37.2	132.5	171.6	0.69	12.5	+112.9
**d**	−179.8	−37.5	−0.5	−20.1	176.9	−34.1	165.2	50.8	1.19	5.3	+190.7
**e**	179.3	−44.1	4.8	−11.7	86.6	−35.6	168.5	165.9	1.53	3.0	+210.3
**f**	176.9	−39.1	14.1	26.1	168.1	31.3	76.1	161.2	1.54	3.0	−66.1
**g**	177.0	−34.2	15.2	24.7	154.3	−37.3	137.1	162.1	1.71	2.2	−7.1
**h**	177.2	−38.2	11.0	22.4	170.4	−33.7	166.9	41.4	1.72	2.2	+59.0
**i**	177.2	−46.1	13.8	24.9	145.5	31.6	173.6	143.7	1.94	1.5	−58.7
**j**	−34.6	−42.3	3.8	−16.7	178.9	29.6	75.6	168.9	2.60	0.5	+151.6
									Average		+91.0

[1] D1: C19C20C23C24, D2: C14C15OH, D3: C19C20C21C22, D4: C4C5NC1, D5: C5C6C7C8, D6: C8C9C10C11, D7: C10C11C12C13, D8: C15C16C17C18. Units in degrees. [2]Units in kcal/mol.

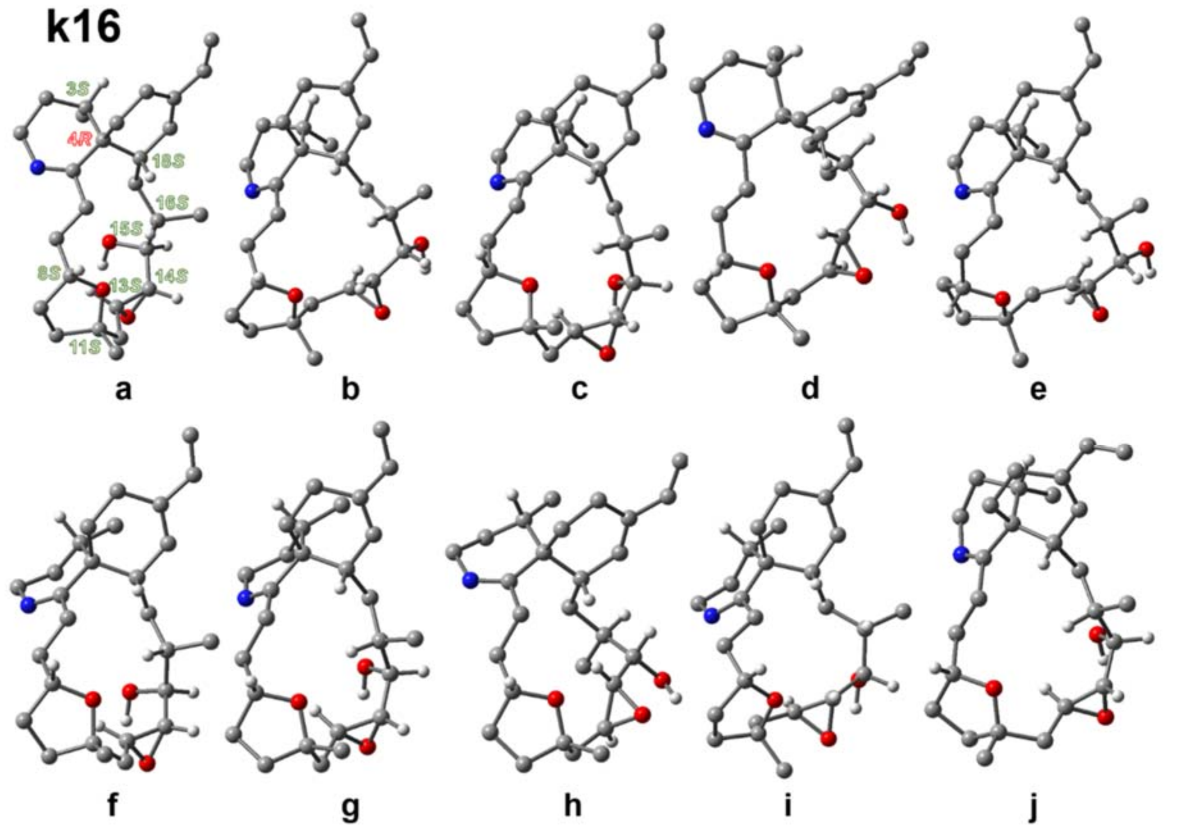

Figure S16. Calculated geometries of ten lowest free energy conformations of k16 isomer, (3*S*, 4*R*, 8*S*, 11*S*, 13*S*, 14*S*, 15*S*, 16*S*, 18*S*).

Table S17. NMR data of portimine (**1**) in free and TFA salt form

Position		Portimine (**1**) free form		Portimine (**1**) TFA salt	
		^{1}H	^{13}C	^{1}H	^{13}C
1	a	3.71 (m)	54.6	4.00 (m)	50.1
	b	3.81 (dd, 14.9, 8.5)	-	4.07 (m)	-
2	a	1.93 (m)	28.3	2.07 (m)	29.7
	b	1.96 (m)	-	2.07 (m)	-
3		-	53.1	-	54.9
4		-	185.8	-	nd
5		4.58 (brs)	65.5	5.04 (brs)	66.4
6	a	1.96 (m)	42.9	2.14 (dd, 16.3, 2.0)	46.2
	b	2.89 (dd, 16.1, 5.7)	-	3.12 (dd, 16.2, 5.5)	-
7		-	108.8	-	110.1
8	a	1.65 (m)	37.4	1.77 (m)	39.3
	b	2.00 (dd)	-	2.00 (dd, 12.3, 6.6)	-
9	a	1.75 (m)	24.0	1.78 (m)	25.2
	b	1.85 (m)	-	1.88 (m)	-
10		4.17 (m)	83.2	4.25 (m)	85.6
11		2.19 (m)	34.1	2.22 (m)	35.1
12	a	1.46 (dd, 14.1, 2.9)	33.7	1.50 (dd, 13.2, 2.9)	34.8
	b	2.29 (ddd, 14.1, 11.3, 10.8)	-	2.22 (m)	-
13		3.91 (dd, 11.5, 2.9)	78.8	3.95 (dd, 11.1, 3.0)	79.8
14		-	204.2	-	204.0
15		4.49 (d, 12.0)	71.2	4.58 (d, 12.1)	71.9
16		3.73 (brd, 10.5)	40.4	4.01 (brd, 9.6)	43.8
17		5.12 (brs)	127.6	5.14 (brs)	126.5
18		-	135.1	-	137.0
19		2.38 (m)	22.2	2.52 (m)	23.2
20		1.80 (m)	28.7	2.25 (m)	27.6
21		6.23 (dd, 17.4, 10.8)	138.6	6.27 (dd, 17.5, 10.8)	139.3
22	a	4.93 (d, 10.8)	110.5	5.02 (d, 10.8)	113.3
	b	5.10 (d, 17.4)	-	5.20 (17.5)	-
23		0.89 (d, 7.1)	16.6	0.91 (d, 6.5)	18.0

(in methanol-d_4)

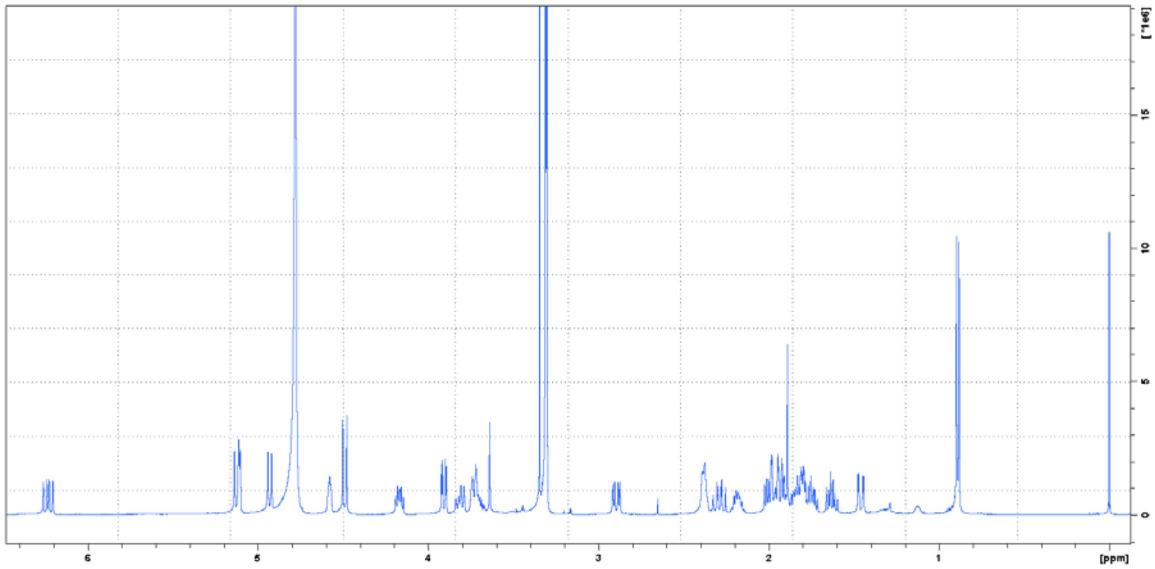

Figure S17. ^{1}H NMR spectrum of portimine (**1**) in methanol-d_4

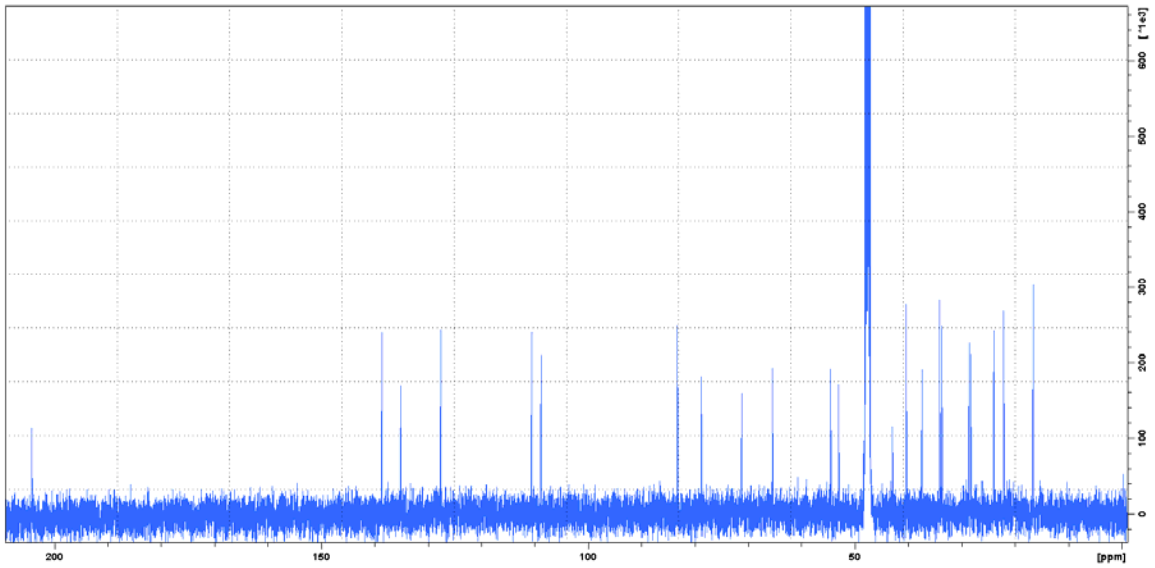

Figure S18. ^{13}C NMR spectrum of portimine (**1**) in methanol-d_4

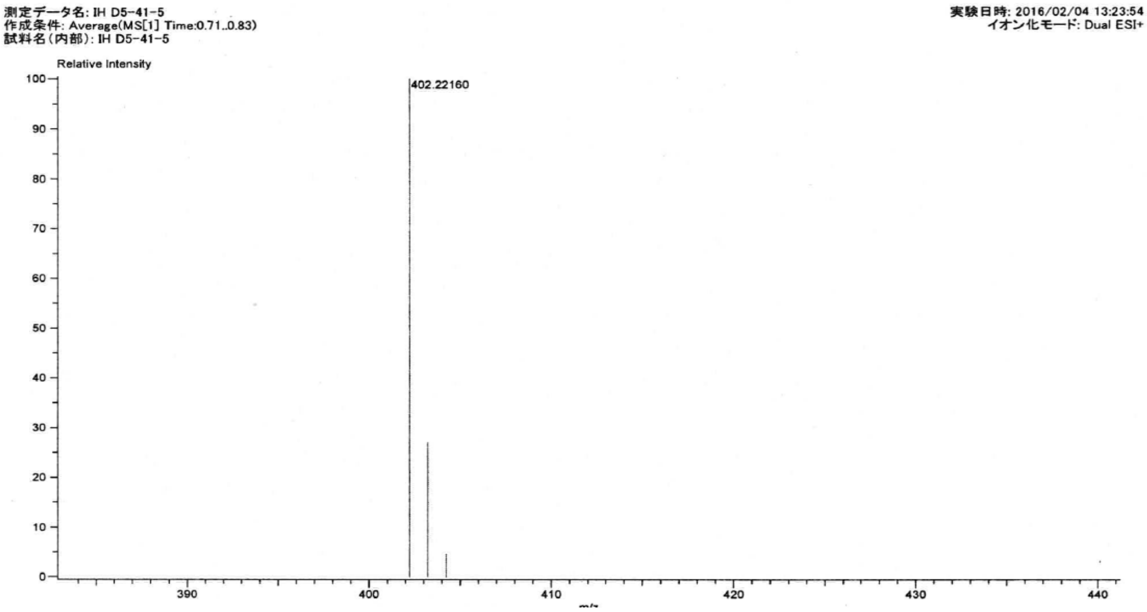

Figure S19. HRESIMS spectrum of portimine (**1**)

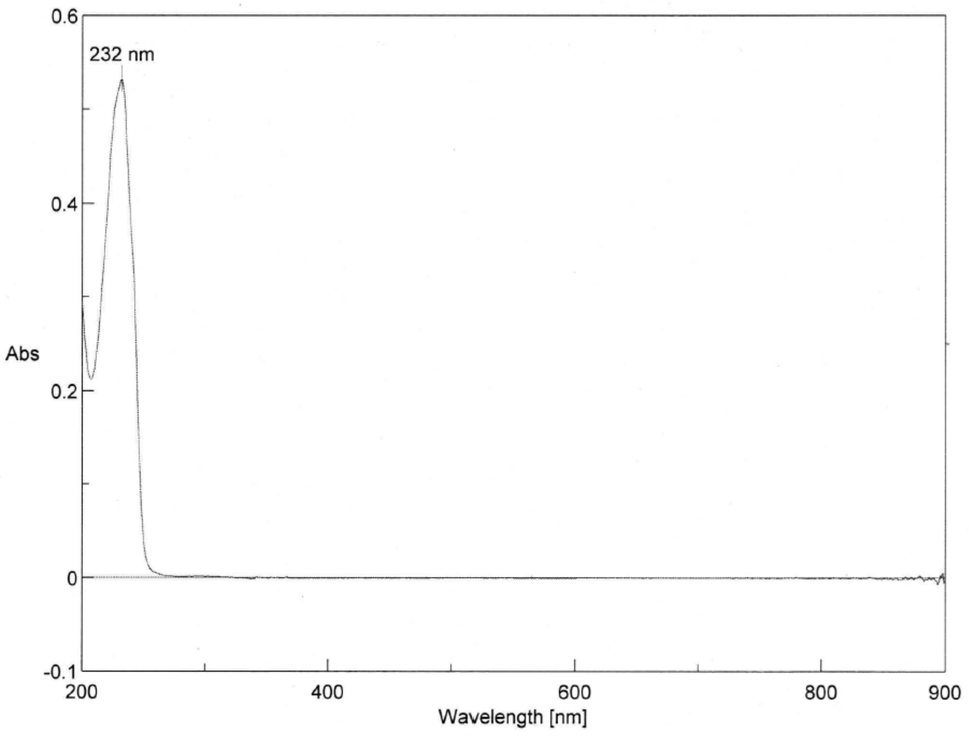

Figure S20. UV spectrum of portimine (**1**) in methanol

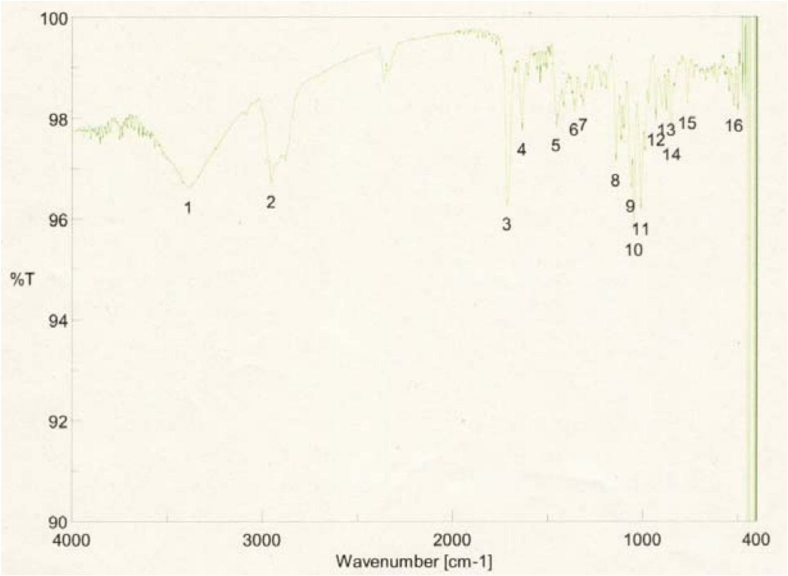

Figure S21. FTIR spectrum of portimine (**1**)

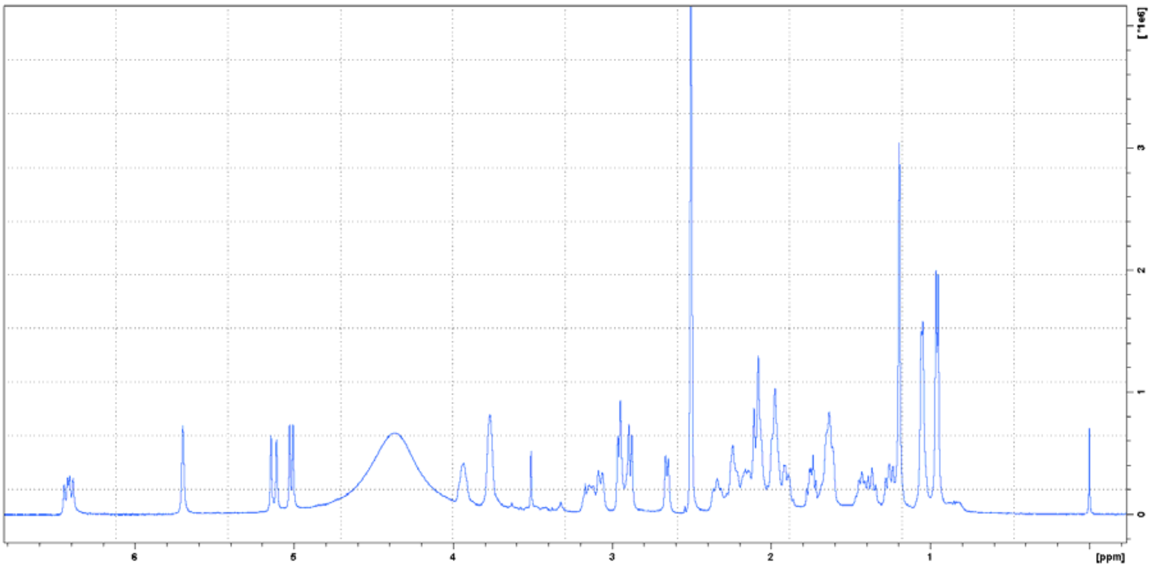

Figure S22. ^{1}H NMR spectrum of kabirimine (**2**) in DMSO-d_6

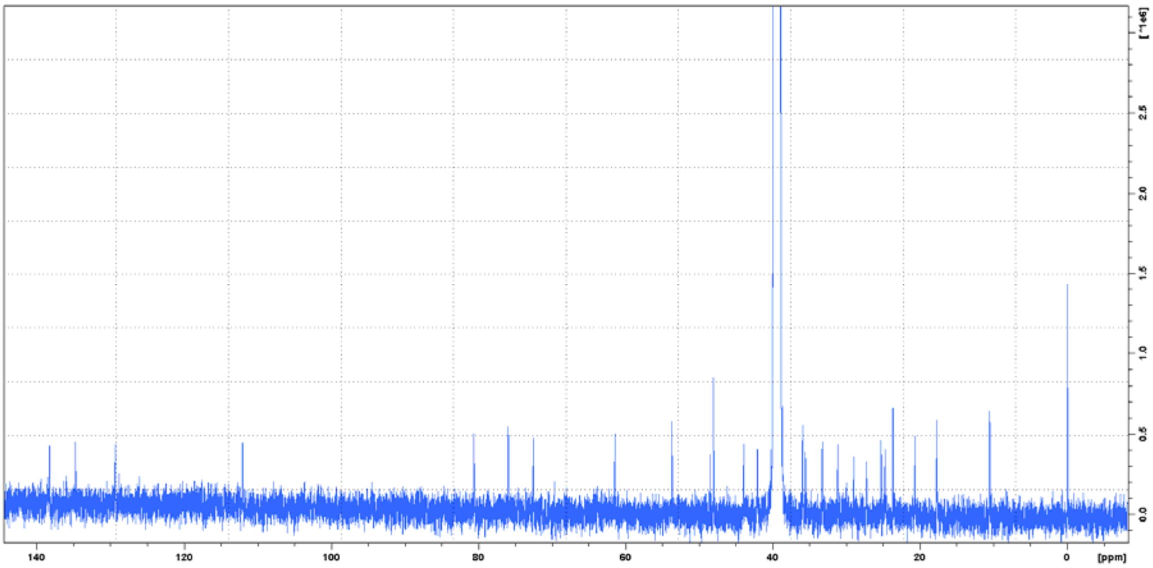

Figure S23. ^{13}C NMR spectrum of kabirimine (**2**) in DMSO-d_6

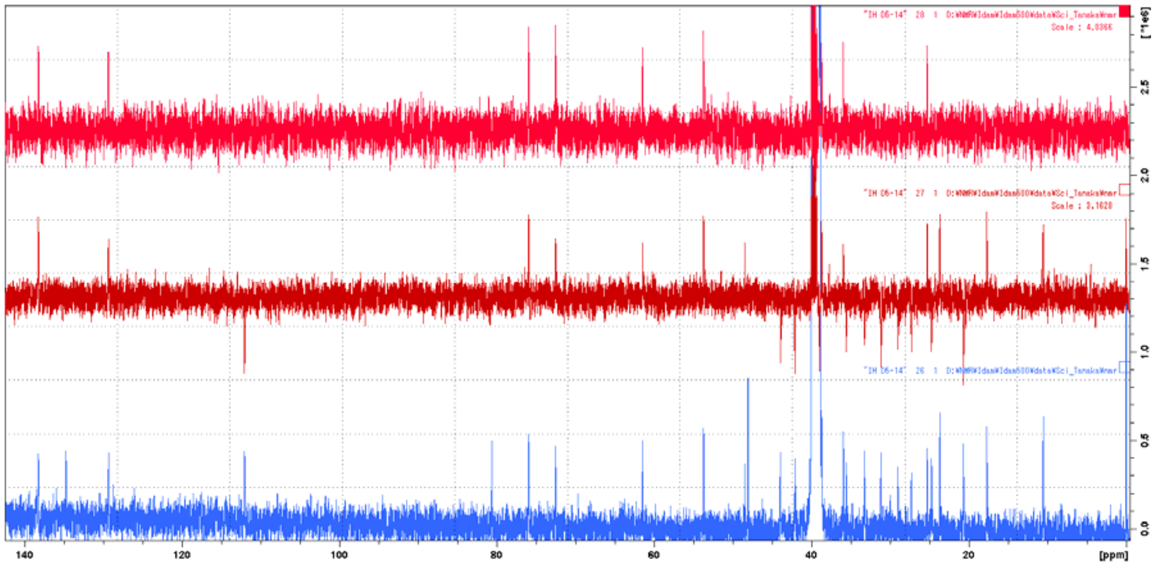

Figure S24. DEPT spectra of kabirimine (**2**) in DMSO-d_6

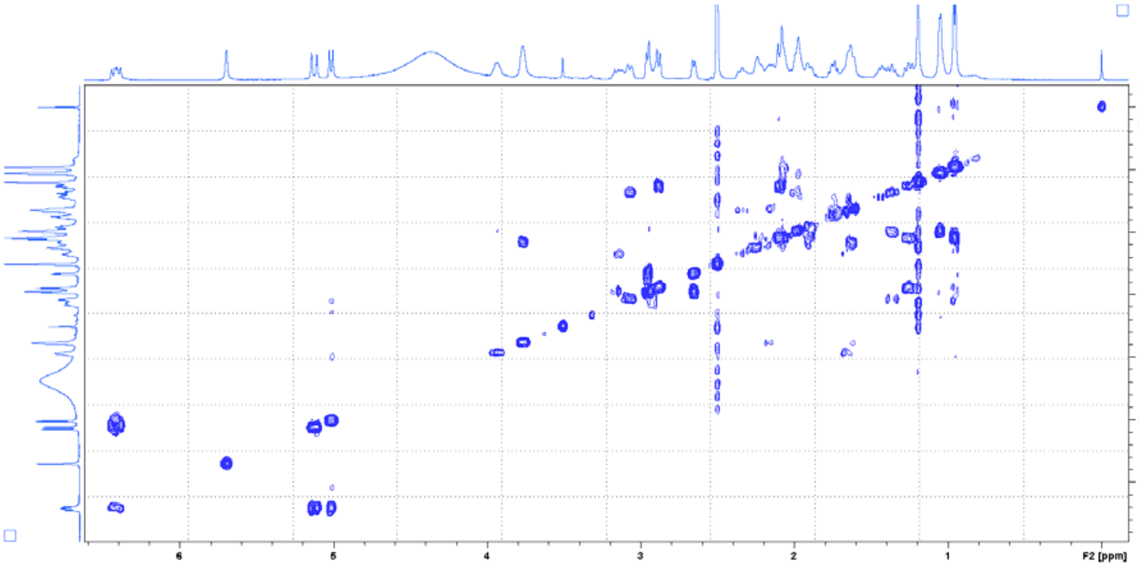

Figure S25. COSY spectrum of kabirimine (**2**) in DMSO-d_6

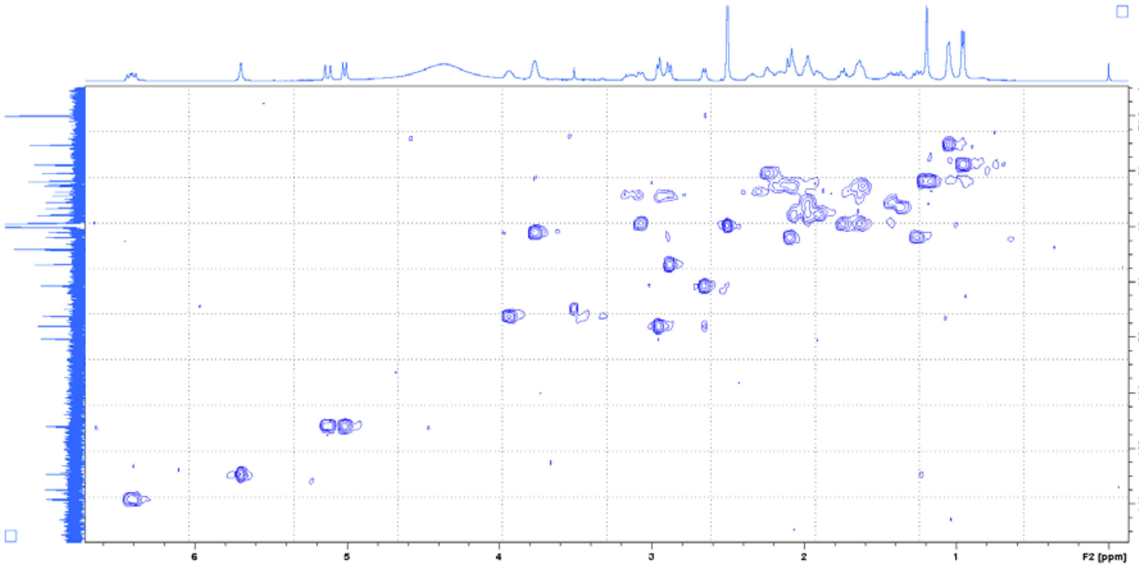

Figure S26. HSQC spectrum of kabirimine (**2**) in DMSO-d_6

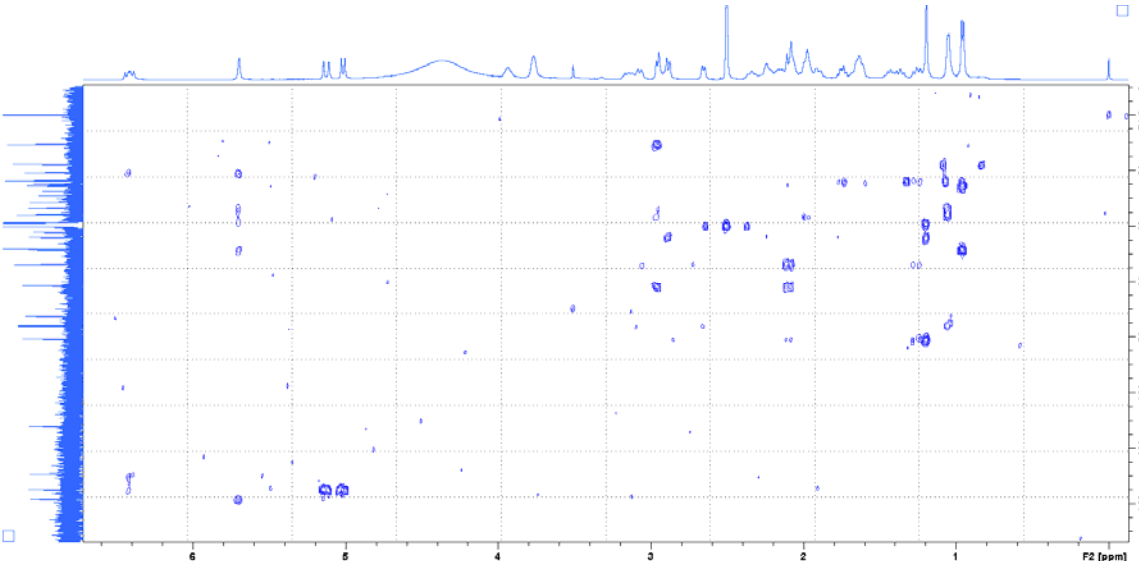

Figure S27. HMBC spectrum of kabirimine (**2**) in DMSO-d_6

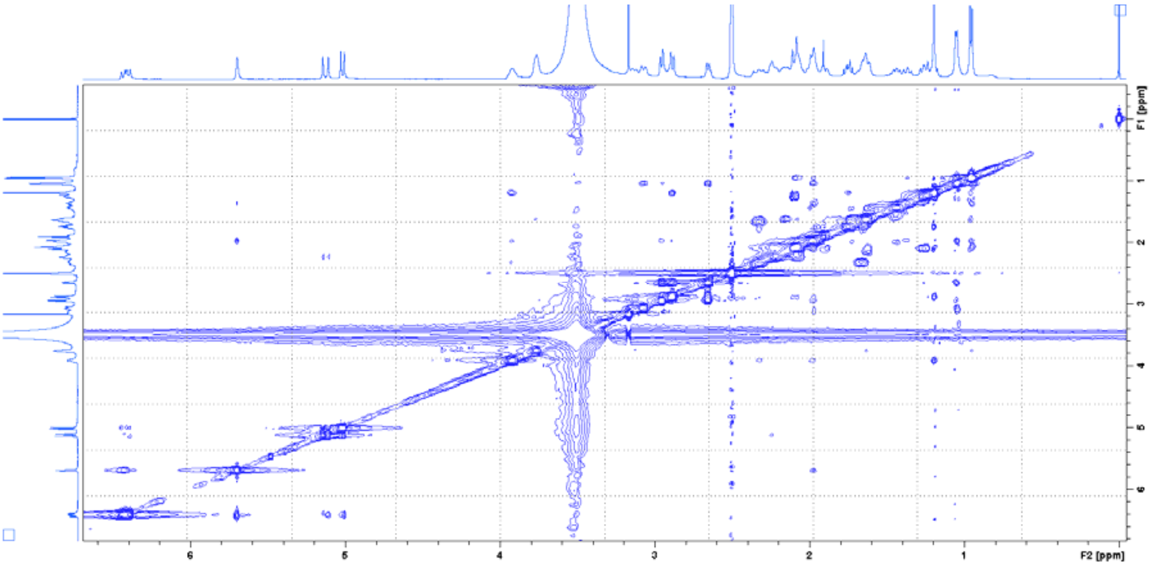

Figure S28. NOESY spectrum of kabirimine (**2**) in DMSO-d_6

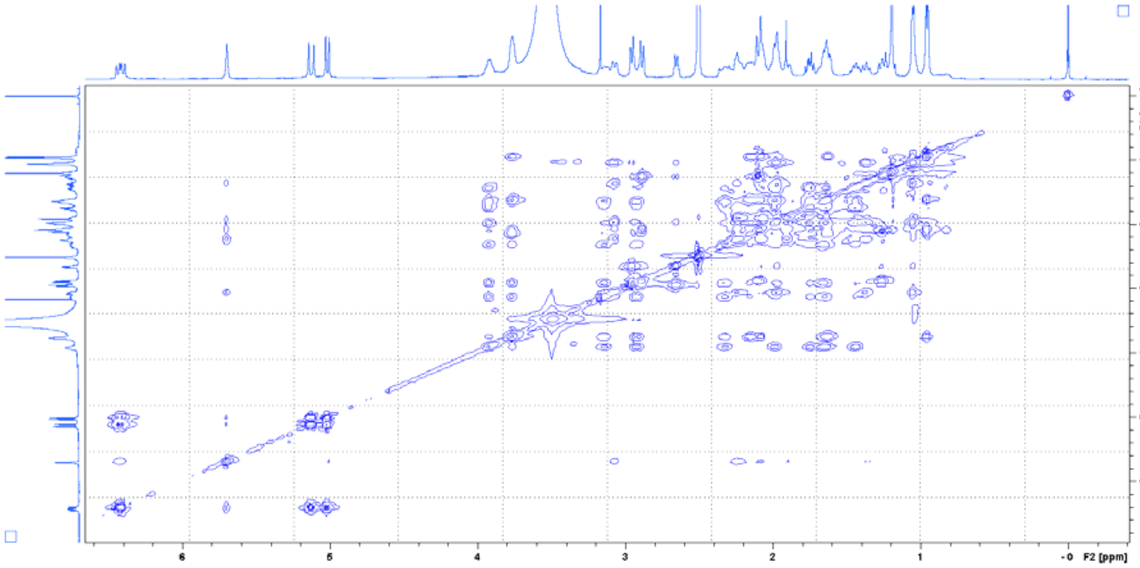

Figure S29. TOCSY spectrum of kabirimine (**2**) in DMSO-d_6

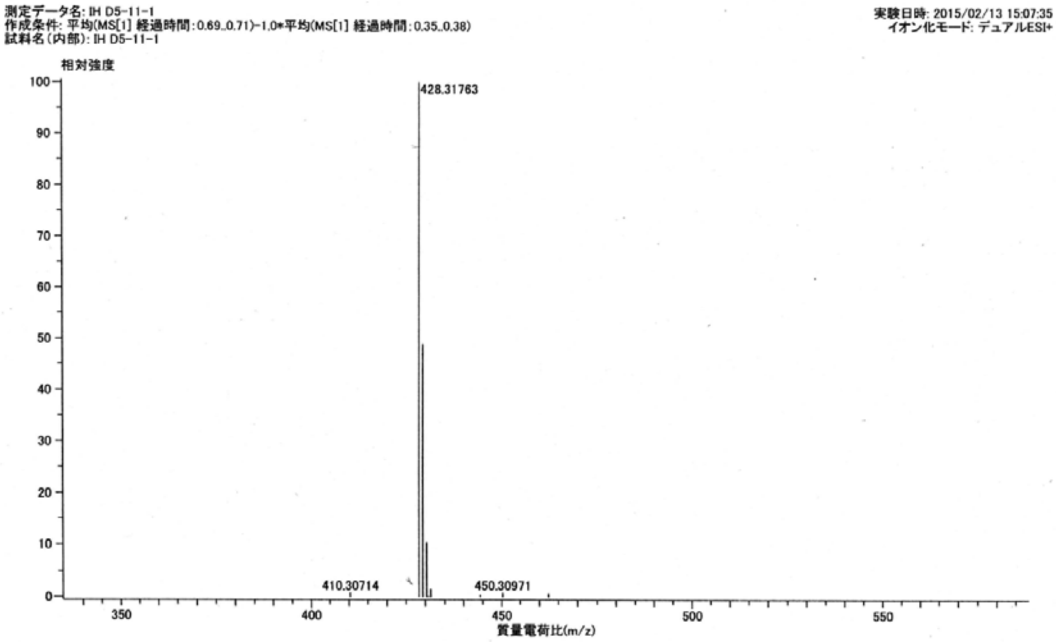

Figure S30. HRESIMS spectrum of kabirimine (**2**)

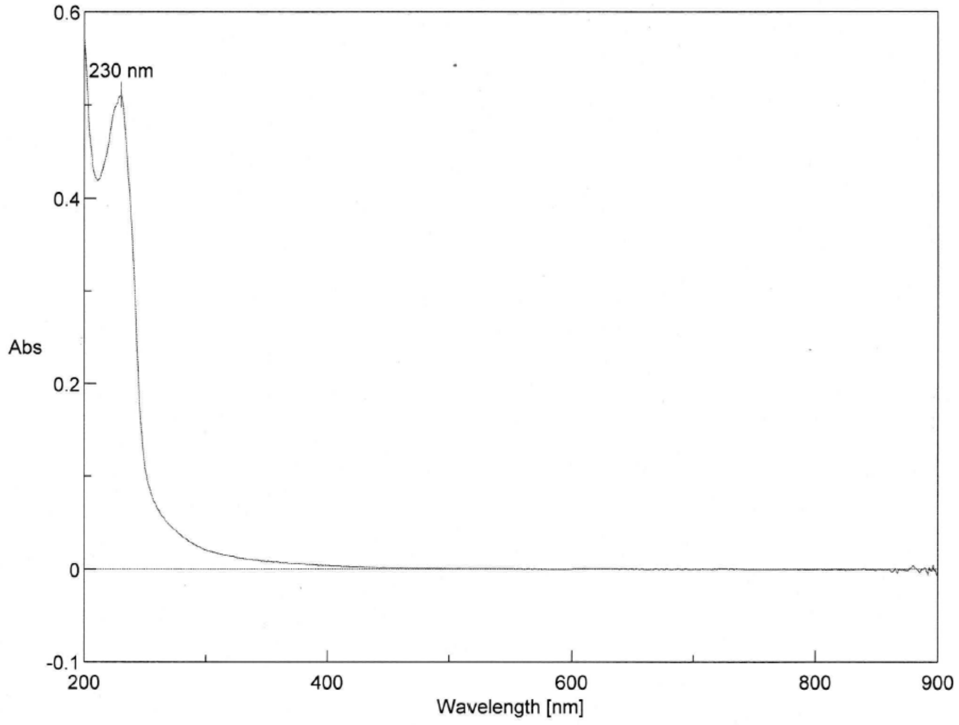

Figure S31. UV spectrum of kabirimine (**2**) in methanol

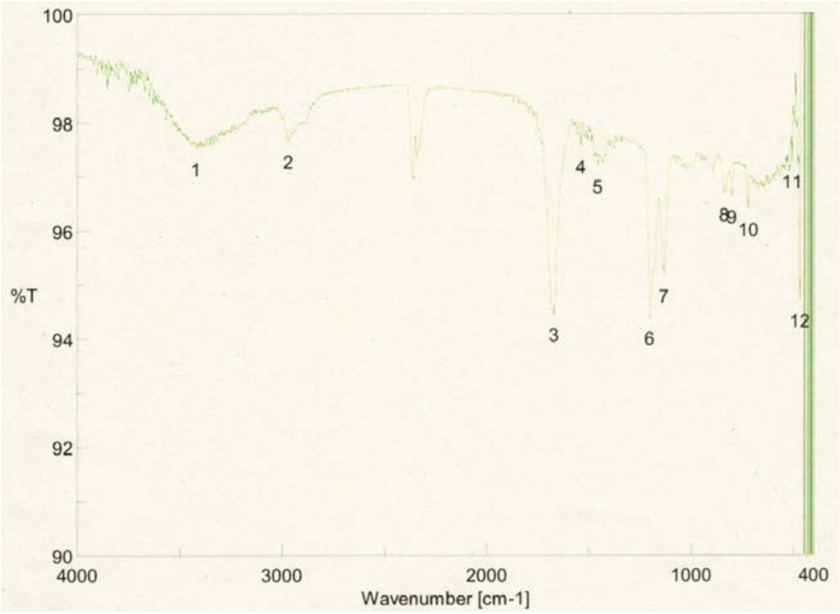

Figure S32. FTIR spectrum of kabirimine (**2**)

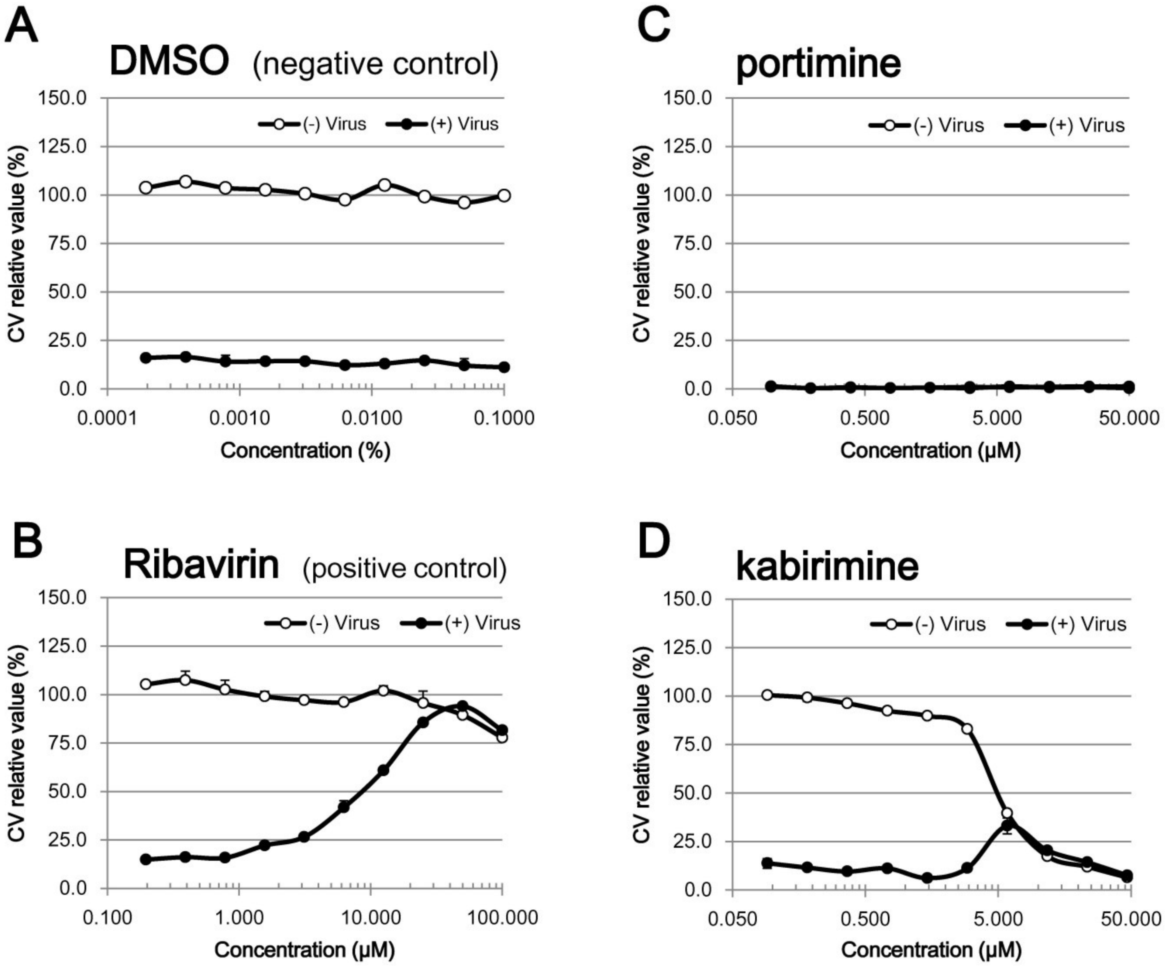

Figure S33. Evaluation of anti-RSV activities and cytotoxicities of portimine (**1**) and kabirimine (**2**).

The assay was performed as described under Experimental section. HEp-2 cells were grown in 96-well plates and infected with RSV (strain A2) in the presence of 2-fold serially diluted the tested compounds. For the evaluation of cytotoxicities, HEp-2 cells were grown in the presence of compounds without viruses. Three days after infection, the cells were fixed and stained with crystal violet (CV), and the absorbance was measured using a plate reader. Relative CV value (%) is expressed as a percentage of the uninfected cells. (A) Dose-response curve of DMSO used the solvent for the tested compounds. (B) Dose-response curve of Ribavirin which is well known to exhibit anti-RSV activities. DMSO and Ribavirin were used as negative and positive control respectively for the validation of the assay. (C) Dose-response curve of portimine (**1**). (D) Dose-response curve of kabirimine (**2**). Open circles represent uninfected groups to evaluate the cytotoxicity. Closed circles represent infected groups to evaluated anti-RSV activity.

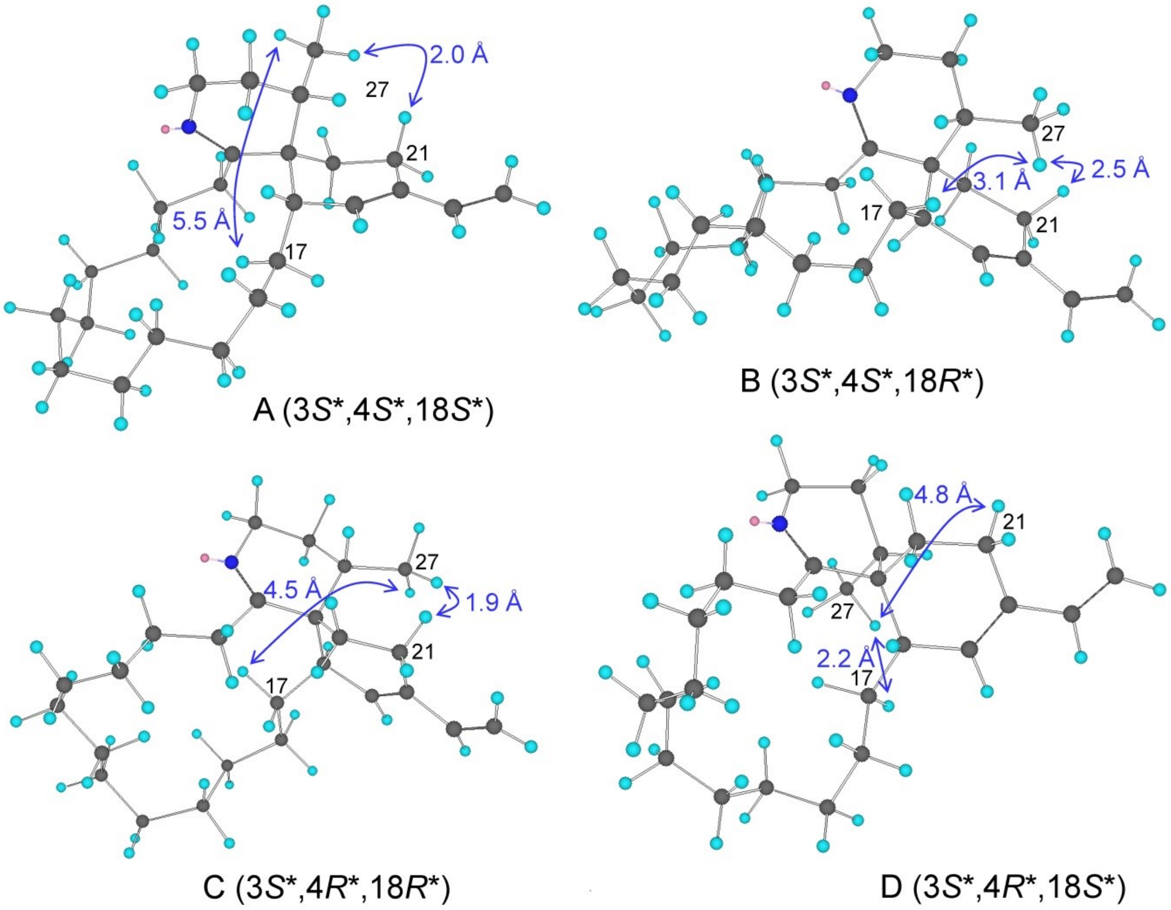

Figure S34. A stable conformation for each model structure A-D was obtained after MM/MD of MM2 in Chem3D. All the structures A-D took a relatively rigid conformation for the spirocyclic portion. The estimated distances for H-17/H-27 and H-21/H-27 were indicated for each molecule.

X-ray Structure Report

for

Portimine_3

April 29, 2016

Experimental

Data Collection

A colorless platelet crystal of $C_{23}H_{31}NO_5$ having approximate dimensions of 0.290 x 0.220 x 0.050 mm was mounted on a glass fiber. All measurements were made on a Rigaku R-AXIS RAPID diffractometer using graphite monochromated Cu-Kα radiation.

The crystal-to-detector distance was 127.40 mm.

Cell constants and an orientation matrix for data collection corresponded to a primitive monoclinic cell with dimensions:

$$
\begin{array}{lll}
a = 8.5456(2)\ \text{Å} & & \\
b = 9.8717(3)\ \text{Å} & & \beta = 104.479(7)^{o} \\
c = 12.8048(3)\ \text{Å} & & \\
V = 1045.90(6)\ \text{Å}^3 & &
\end{array}
$$

For Z = 2 and F.W. = 401.50, the calculated density is 1.275 g/cm^3. Based on the reflection conditions of:

0k0: k = 2n

packing considerations, a statistical analysis of intensity distribution, and the successful solution and refinement of the structure, the space group was determined to be:

$P2_1$ (#4)

The data were collected at a temperature of 23 $\pm$ 1°C to a maximum 2θ value of 136.4^{o}. A total of 180 oscillation images were collected. A sweep of data was done using ω scans from 80.0 to 260.0^{o} in 5.00^{o} step, at χ=54.0^{o} and ϕ = 0.0^{o}. The exposure rate was 60.0 [sec./^{o}]. A second sweep was performed using ω scans from 80.0 to 260.0^{o} in 5.00^{o} step, at χ=54.0^{o} and ϕ = 90.0^{o}. The exposure rate was 60.0

[sec./^{o}]. Another sweep was performed using ω scans from 80.0 to 260.0^{o} in 5.00^{o} step, at χ=54.0^{o} and ϕ = 180.0^{o}. The exposure rate was 60.0 [sec./^{o}]. Another sweep was performed using ω scans from 80.0 to 260.0^{o} in 5.00^{o} step, at χ=54.0^{o} and ϕ = 270.0^{o}. The exposure rate was 60.0 [sec./^{o}]. Another sweep was performed using ω scans from 80.0 to 260.0^{o} in 5.00^{o} step, at χ=0.0^{o} and ϕ = 0.0^{o}. The exposure rate was 60.0 [sec./^{o}]. The crystal-to-detector distance was 127.40 mm. Readout was performed in the 0.100 mm pixel mode.

Data Reduction

Of the 11959 reflections were collected, where 3680 were unique (R_{int} = 0.0563); equivalent reflections were merged.

The linear absorption coefficient, μ, for Cu-Kα radiation is 7.246 cm^{-1}. An empirical absorption correction was applied which resulted in transmission factors ranging from 0.679 to 0.964. The data were corrected for Lorentz and polarization effects.

Structure Solution and Refinement

The structure was solved by direct methods[1] and expanded using Fourier techniques. The non-hydrogen atoms were refined anisotropically. Hydrogen atoms were refined using the riding model. The final cycle of full-matrix least-squares refinement[2] on F^2 was based on 3680 observed reflections and 270 variable parameters and converged (largest parameter shift was 0.00 times its esd) with unweighted and weighted agreement factors of:

$$R1 = \Sigma ||Fo| - |Fc|| / \Sigma |Fo| = 0.0600$$

$$wR2 = [\Sigma (w (Fo^2 - Fc^2)^2)/ \Sigma w(Fo^2)^2]^{1/2} = 0.1543$$

The goodness of fit[3] was 1.11. Unit weights were used. The maximum and minimum peaks on the final difference Fourier map corresponded to 0.20 and -0.29 $e^-/Å^3$, respectively. The final Flack parameter [4] was 0.11(16), indicating that the present absolute structure is correct. [5]

Neutral atom scattering factors were taken from International Tables for Crystallography (IT), Vol. C, Table 6.1.1.4 [6]. Anomalous dispersion effects were included in Fcalc[7]; the values for Δf' and Δf" were those of Creagh and McAuley[8]. The values for the mass attenuation coefficients are those of Creagh and Hubbell[9]. All calculations were performed using the CrystalStructure[10] crystallographic software package except for refinement, which was performed using SHELXL Version 2014/7[11].

References

(1) SHELXT Version 2014/5: Sheldrick, G. M. (2014). Acta Cryst. A70, C1437.

(2) Least Squares function minimized: (SHELXL Version 2014/7)

$\Sigma w(F_o^2-F_c^2)^2$ where w = Least Squares weights.

(3) Goodness of fit is defined as:

$[\Sigma w(F_o^2-F_c^2)^2/(N_o-N_v)]^{1/2}$

where: N_o = number of observations
N_v = number of variables

(4) Parsons, S. and Flack, H. (2004), Acta Cryst. A60, s61.

(5) Flack, H.D. and Bernardinelli (2000), J. Appl. Cryst. 33, 114-1148.

(6) International Tables for Crystallography, Vol.C (1992). Ed. A.J.C. Wilson, Kluwer Academic Publishers, Dordrecht, Netherlands, Table 6.1.1.4, pp. 572.

(7) Ibers, J. A. & Hamilton, W. C.; Acta Crystallogr., 17, 781 (1964).

(8) Creagh, D. C. & McAuley, W.J .; "International Tables for Crystallography", Vol C, (A.J.C. Wilson, ed.), Kluwer Academic Publishers, Boston, Table 4.2.6.8, pages 219-222 (1992).

(9) Creagh, D. C. & Hubbell, J.H..; "International Tables for Crystallography", Vol C, (A.J.C. Wilson, ed.), Kluwer Academic Publishers, Boston, Table 4.2.4.3, pages 200-206 (1992).

(10) CrystalStructure 4.2: Crystal Structure Analysis Package, Rigaku Corporation (2000-2015). Tokyo 196-8666, Japan.

(11) SHELXL Version 2014/7: Sheldrick, G. M. (2008). Acta Cryst. A64, 112-122.

EXPERIMENTAL DETAILS

A. Crystal Data

Empirical Formula	$C_{23}H_{31}NO_5$
Formula Weight	401.50
Crystal Color, Habit	colorless, platelet
Crystal Dimensions	0.290 X 0.220 X 0.050 mm
Crystal System	monoclinic
Lattice Type	Primitive
Lattice Parameters	a = 8.5456(2) Å<br>b = 9.8717(3) Å<br>c = 12.8048(3) Å<br>β = 104.479(7) ^{o}<br>V = 1045.90(6) $Å^3$
Space Group	$P2_1$ (#4)
Z value	2
D_{calc}	1.275 g/cm^3
F_{000}	432.00
$\mu(CuK\alpha)$	7.246 cm^{-1}

B. Intensity Measurements

Diffractometer	R-AXIS RAPID
Radiation	CuKα (λ = 1.54187 Å)<br>graphite monochromated
Voltage, Current	50kV, 40mA
Temperature	23.0^{o}C
Detector Aperture	460.0 x 256.0 mm
Data Images	180 exposures
ω oscillation Range (χ=54.0, ϕ=0.0)	80.0 - 260.0^{o}
Exposure Rate	60.0 sec./^{o}
ω oscillation Range (χ=54.0, ϕ=90.0)	80.0 - 260.0^{o}
Exposure Rate	60.0 sec./^{o}
ω oscillation Range (χ=54.0, ϕ=180.0)	80.0 - 260.0^{o}
Exposure Rate	60.0 sec./^{o}
ω oscillation Range (χ=54.0, ϕ=270.0)	80.0 - 260.0^{o}
Exposure Rate	60.0 sec./^{o}
ω oscillation Range (χ=0.0, ϕ=0.0)	80.0 - 260.0^{o}
Exposure Rate	60.0 sec./^{o}
Detector Position	127.40 mm
Pixel Size	0.100 mm

$2\theta_{max}$	136.4^{o}
No. of Reflections Measured	Total: 11959<br>Unique: 3680 (R_{int} = 0.0563)<br>Parsons quotients (Flack x parameter):
940	
Corrections	Lorentz-polarization<br>Absorption<br>(trans. factors: 0.679 - 0.964)

C. Structure Solution and Refinement

Structure Solution	Direct Methods (SHELXT Version 2014/5)
Refinement	Full-matrix least-squares on F^2
Function Minimized	Σ w $(Fo^2 - Fc^2)^2$
Least Squares Weights	w = 1/ [$\sigma^2(Fo^2)$ + $(0.0777 \cdot P)^2$ + 0.0402 · P] where P = $(Max(Fo^2,0) + 2Fc^2)/3$
$2\theta_{max}$ cutoff	136.4^o
Anomalous Dispersion	All non-hydrogen atoms
No. Observations (All reflections)	3680
No. Variables	270
Reflection/Parameter Ratio	13.63
Residuals: R1 (I>2.00σ(I))	0.0600
Residuals: R (All reflections)	0.0755
Residuals: wR2 (All reflections)	0.1543
Goodness of Fit Indicator	1.111
Flack parameter (Parsons' quotients = 940)	0.11(16)
Max Shift/Error in Final Cycle	0.001
Maximum peak in Final Diff. Map	0.20 $e^-/Å^3$
Minimum peak in Final Diff. Map	-0.29 $e^-/Å^3$

Table S18. Atomic coordinates and B_{iso}/B_{eq}

atom	x	y	z	B_{eq}
O1	0.2895(4)	0.5676(3)	0.2531(2)	2.31(6)
O2	0.3227(4)	0.4295(3)	0.1122(3)	2.46(6)
O3	0.6297(4)	0.3762(3)	0.2944(3)	2.80(7)
O4	0.7438(4)	0.6663(4)	0.2623(3)	3.09(7)
O5	-0.0563(5)	0.3163(5)	0.2679(4)	4.67(10)
N6	0.0179(5)	0.5438(5)	0.3938(4)	3.13(9)
C7	0.2950(6)	0.4989(5)	0.4697(4)	2.46(9)
C8	0.4447(5)	0.4693(5)	0.4268(3)	2.21(8)
C9	0.4475(5)	0.5474(5)	0.3240(4)	2.25(8)
C10	0.5688(6)	0.4859(5)	0.2655(4)	2.42(9)
C11	0.2196(6)	0.4586(5)	0.1810(4)	2.39(9)
C12	0.2029(6)	0.3282(5)	0.2385(4)	2.48(9)
C13	0.5982(6)	0.4873(5)	0.5129(4)	2.67(9)
C14	0.6378(5)	0.5744(5)	0.1903(4)	2.66(9)
C15	0.1339(6)	0.4634(5)	0.3915(4)	2.66(9)
C16	0.2655(6)	0.6490(5)	0.4891(4)	2.82(10)
C17	0.1062(6)	0.3382(6)	0.3237(4)	3.00(10)
C18	0.3131(7)	0.4155(6)	0.5733(4)	3.22(11)
C19	0.5255(6)	0.6619(6)	0.1070(4)	2.96(10)
C20	0.4336(6)	0.5880(6)	0.0041(4)	2.97(10)
C21	0.6113(6)	0.4760(5)	0.6186(4)	2.96(10)
C22	0.2822(6)	0.5173(5)	0.0163(4)	2.82(10)
C23	0.0696(6)	0.5223(6)	0.1090(4)	2.93(10)
C24	0.7701(8)	0.4779(6)	0.6953(5)	3.83(12)
C25	0.4636(6)	0.4536(6)	0.6611(4)	3.41(11)
C26	0.1427(6)	0.6064(5)	0.0321(4)	3.10(10)
C27	0.0809(6)	0.6572(6)	0.4701(5)	3.53(11)
C28	0.3887(7)	0.6847(6)	-0.0916(4)	4.12(13)
C29	0.8024(9)	0.4571(7)	0.8008(5)	5.39(16)

$B_{eq} = 8/3\ \pi^2(U_{11}(aa^*)^2 + U_{22}(bb^*)^2 + U_{33}(cc^*)^2 + 2U_{12}(aa^*bb^*)\cos\gamma + 2U_{13}(aa^*cc^*)\cos\beta + 2U_{23}(bb^*cc^*)\cos\alpha)$

Table S19. Atomic coordinates and B_{iso} involving hydrogen atoms

atom	x	y	z	B_{iso}
H4	0.80812	0.62293	0.30843	3.705
H8	0.43899	0.37309	0.40735	2.655
H9	0.48863	0.63797	0.34763	2.702
H12A	0.31022	0.29502	0.27294	2.970
H12B	0.15160	0.26162	0.18518	2.970
H13	0.69137	0.50783	0.49110	3.203
H14	0.70226	0.51766	0.15410	3.195
H16A	0.30294	0.70625	0.43866	3.383
H16B	0.31952	0.67573	0.56209	3.383
H17	0.13856	0.26124	0.37268	3.600
H18A	0.21822	0.42897	0.60067	3.866
H18B	0.31849	0.32013	0.55619	3.866
H19A	0.58840	0.73434	0.08642	3.553
H19B	0.44709	0.70325	0.14034	3.553
H20	0.50591	0.51893	-0.01272	3.562
H22	0.24161	0.46010	-0.04725	3.380
H23A	0.01387	0.57916	0.14986	3.513
H23B	-0.00436	0.45410	0.07039	3.513
H24	0.86510	0.49892	0.66138	4.467
H25A	0.44158	0.53561	0.69657	4.090
H25B	0.48631	0.38200	0.71465	4.090
H26A	0.06406	0.62212	-0.03584	3.719
H26B	0.18218	0.69287	0.06404	3.719
H27A	0.04934	0.64554	0.53721	4.240
H27B	0.04060	0.74369	0.43862	4.240
H28A	0.48412	0.73016	-0.10010	4.947
H28B	0.34137	0.63435	-0.15594	4.947
H28C	0.31252	0.75032	-0.07921	4.947
H29A	0.71896	0.43889	0.83349	6.463
H29B	0.90861	0.46065	0.84222	6.463
H30	-0.10986	0.35312	0.30298	12.653

Table S20. Anisotropic displacement parameters

atom	U_{11}	U_{22}	U_{33}	U_{12}	U_{13}	U_{23}
O1	0.0270(17)	0.0240(17)	0.0345(17)	-0.0014(14)	0.0032(13)	-0.0032(15)
O2	0.0342(19)	0.0277(18)	0.0333(18)	0.0019(15)	0.0116(15)	0.0016(15)
O3	0.0337(19)	0.0281(19)	0.044(2)	0.0021(16)	0.0093(16)	0.0024(16)
O4	0.034(2)	0.036(2)	0.044(2)	-0.0042(17)	0.0042(16)	-0.0004(18)
O5	0.031(2)	0.068(3)	0.080(3)	-0.011(2)	0.017(2)	-0.032(3)
N6	0.033(2)	0.038(3)	0.049(3)	0.002(2)	0.014(2)	-0.007(2)
C7	0.030(3)	0.029(3)	0.037(3)	0.004(2)	0.012(2)	-0.002(2)
C8	0.028(2)	0.027(3)	0.028(2)	0.001(2)	0.005(2)	0.002(2)
C9	0.025(2)	0.028(3)	0.029(2)	0.001(2)	0.0025(19)	0.002(2)
C10	0.030(3)	0.028(3)	0.032(3)	-0.003(2)	0.004(2)	0.001(2)
C11	0.028(3)	0.025(3)	0.037(3)	-0.003(2)	0.007(2)	-0.002(2)
C12	0.031(3)	0.030(3)	0.034(3)	-0.006(2)	0.009(2)	-0.004(2)
C13	0.033(3)	0.028(3)	0.038(3)	0.003(2)	0.005(2)	0.000(2)
C14	0.029(3)	0.034(3)	0.037(3)	-0.006(2)	0.006(2)	0.002(2)
C15	0.030(3)	0.034(3)	0.038(3)	-0.001(2)	0.011(2)	-0.004(2)
C16	0.042(3)	0.028(3)	0.040(3)	0.001(2)	0.015(2)	-0.004(2)
C17	0.031(3)	0.041(3)	0.042(3)	-0.008(2)	0.011(2)	-0.005(3)
C18	0.049(3)	0.038(3)	0.038(3)	-0.002(3)	0.016(3)	0.002(2)
C19	0.044(3)	0.030(3)	0.038(3)	-0.003(2)	0.009(2)	0.002(2)
C20	0.045(3)	0.039(3)	0.030(3)	0.002(3)	0.012(2)	0.005(2)
C21	0.047(3)	0.025(3)	0.034(3)	-0.002(2)	-0.002(2)	0.000(2)
C22	0.039(3)	0.032(3)	0.031(3)	-0.003(2)	0.001(2)	0.006(2)
C23	0.029(3)	0.039(3)	0.038(3)	0.002(2)	-0.001(2)	-0.001(2)
C24	0.055(4)	0.031(3)	0.046(3)	-0.002(3)	-0.012(3)	0.001(3)
C25	0.059(4)	0.037(3)	0.032(3)	-0.002(3)	0.009(3)	-0.005(2)
C26	0.041(3)	0.035(3)	0.035(3)	0.003(2)	-0.003(2)	0.002(2)
C27	0.043(3)	0.036(3)	0.059(4)	0.009(3)	0.018(3)	-0.016(3)
C28	0.069(4)	0.044(4)	0.043(3)	-0.006(3)	0.013(3)	0.012(3)
C29	0.088(5)	0.054(4)	0.043(3)	-0.016(4)	-0.020(3)	0.010(3)

The general temperature factor expression: $\exp(-2\pi^2(a^{*2}U_{11}h^2 + b^{*2}U_{22}k^2 + c^{*2}U_{33}l^2 + 2a^*b^*U_{12}hk + 2a^*c^*U_{13}hl + 2b^*c^*U_{23}kl))$

Table S21. Bond lengths (Å)

atom	atom	distance	atom	atom	distance
O1	C9	1.441(5)	O1	C11	1.446(5)
O2	C11	1.423(7)	O2	C22	1.471(6)
O3	C10	1.219(6)	O4	C14	1.441(6)
O5	C17	1.410(6)	N6	C15	1.276(7)
N6	C27	1.494(7)	C7	C8	1.542(7)
C7	C15	1.527(6)	C7	C16	1.534(7)
C7	C18	1.535(7)	C8	C9	1.531(7)
C8	C13	1.498(6)	C9	C10	1.546(7)
C10	C14	1.523(7)	C11	C12	1.507(7)
C11	C23	1.516(6)	C12	C17	1.527(8)
C13	C21	1.334(7)	C14	C19	1.515(7)
C15	C17	1.495(7)	C16	C27	1.537(7)
C18	C25	1.528(7)	C19	C20	1.538(7)
C20	C22	1.513(8)	C20	C28	1.525(8)
C21	C24	1.463(7)	C21	C25	1.510(8)
C22	C26	1.535(8)	C23	C26	1.535(8)
C24	C29	1.327(8)			

Table S22. Bond lengths involving hydrogens (Å)

atom	atom	distance	atom	atom	distance
O4	H4	0.820	O5	H30	0.804
C8	H8	0.980	C9	H9	0.980
C12	H12A	0.970	C12	H12B	0.970
C13	H13	0.930	C14	H14	0.980
C16	H16A	0.970	C16	H16B	0.970
C17	H17	0.980	C18	H18A	0.970
C18	H18B	0.970	C19	H19A	0.970
C19	H19B	0.970	C20	H20	0.980
C22	H22	0.980	C23	H23A	0.970
C23	H23B	0.970	C24	H24	1.033
C25	H25A	0.970	C25	H25B	0.970
C26	H26A	0.970	C26	H26B	0.970
C27	H27A	0.970	C27	H27B	0.970
C28	H28A	0.960	C28	H28B	0.960
C28	H28C	0.960	C29	H29A	0.930
C29	H29B	0.930			

Table S23. Bond angles (^{o})

atom	atom	atom	angle	atom	atom	atom	angle
C9	O1	C11	118.2(3)	C11	O2	C22	110.2(3)
C15	N6	C27	109.0(4)	C8	C7	C15	114.5(4)
C8	C7	C16	115.2(4)	C8	C7	C18	107.0(4)
C15	C7	C16	99.8(4)	C15	C7	C18	108.9(4)
C16	C7	C18	111.3(4)	C7	C8	C9	113.8(4)
C7	C8	C13	111.5(4)	C9	C8	C13	112.0(4)
O1	C9	C8	113.5(4)	O1	C9	C10	112.6(4)
C8	C9	C10	112.0(4)	O3	C10	C9	119.3(4)
O3	C10	C14	119.9(5)	C9	C10	C14	119.6(4)
O1	C11	O2	108.9(4)	O1	C11	C12	113.5(4)
O1	C11	C23	103.4(4)	O2	C11	C12	106.2(4)
O2	C11	C23	105.5(4)	C12	C11	C23	118.9(4)
C11	C12	C17	115.3(4)	C8	C13	C21	125.0(5)
O4	C14	C10	103.8(4)	O4	C14	C19	106.1(4)
C10	C14	C19	119.7(4)	N6	C15	C7	114.8(4)
N6	C15	C17	121.1(4)	C7	C15	C17	123.8(4)
C7	C16	C27	103.2(4)	O5	C17	C12	105.6(4)
O5	C17	C15	114.5(5)	C12	C17	C15	115.6(5)
C7	C18	C25	112.8(5)	C14	C19	C20	115.5(4)
C19	C20	C22	113.4(4)	C19	C20	C28	111.4(4)
C22	C20	C28	108.8(4)	C13	C21	C24	120.5(5)
C13	C21	C25	120.8(4)	C24	C21	C25	118.6(5)
O2	C22	C20	109.2(4)	O2	C22	C26	104.8(4)
C20	C22	C26	117.5(4)	C11	C23	C26	101.4(4)
C21	C24	C29	127.2(7)	C18	C25	C21	113.4(4)
C22	C26	C23	103.2(4)	N6	C27	C16	104.6(4)

Table S24. Bond angles involving hydrogens (^{o})

atom	atom	atom	angle	atom	atom	atom	angle
C14	O4	H4	109.5	C17	O5	H30	105.9
C7	C8	H8	106.3	C9	C8	H8	106.3
C13	C8	H8	106.3	O1	C9	H9	106.0
C8	C9	H9	106.0	C10	C9	H9	106.0
C11	C12	H12A	108.4	C11	C12	H12B	108.4
C17	C12	H12A	108.4	C17	C12	H12B	108.4
H12A	C12	H12B	107.5	C8	C13	H13	117.5
C21	C13	H13	117.5	O4	C14	H14	108.9
C10	C14	H14	108.9	C19	C14	H14	108.9
C7	C16	H16A	111.1	C7	C16	H16B	111.1
C27	C16	H16A	111.1	C27	C16	H16B	111.1
H16A	C16	H16B	109.1	O5	C17	H17	106.9
C12	C17	H17	106.9	C15	C17	H17	106.9
C7	C18	H18A	109.0	C7	C18	H18B	109.0
C25	C18	H18A	109.0	C25	C18	H18B	109.0
H18A	C18	H18B	107.8	C14	C19	H19A	108.4
C14	C19	H19B	108.4	C20	C19	H19A	108.4
C20	C19	H19B	108.4	H19A	C19	H19B	107.5
C19	C20	H20	107.7	C22	C20	H20	107.7
C28	C20	H20	107.7	O2	C22	H22	108.3
C20	C22	H22	108.3	C26	C22	H22	108.3
C11	C23	H23A	111.5	C11	C23	H23B	111.5
C26	C23	H23A	111.5	C26	C23	H23B	111.5
H23A	C23	H23B	109.3	C21	C24	H24	114.6
C29	C24	H24	118.3	C18	C25	H25A	108.9
C18	C25	H25B	108.9	C21	C25	H25A	108.9
C21	C25	H25B	108.9	H25A	C25	H25B	107.7
C22	C26	H26A	111.1	C22	C26	H26B	111.1
C23	C26	H26A	111.1	C23	C26	H26B	111.1
H26A	C26	H26B	109.1	N6	C27	H27A	110.8
N6	C27	H27B	110.8	C16	C27	H27A	110.8
C16	C27	H27B	110.8	H27A	C27	H27B	108.9
C20	C28	H28A	109.5	C20	C28	H28B	109.5
C20	C28	H28C	109.5	H28A	C28	H28B	109.5
H28A	C28	H28C	109.5	H28B	C28	H28C	109.5
C24	C29	H29A	120.0	C24	C29	H29B	120.0
H29A	C29	H29B	120.0				

Table S25. Torsion Angles(^{o})

(Those having bond angles > 160 or < 20 degrees are excluded.)

atom1	atom2	atom3	atom4	angle	atom1	atom2	atom3	atom4	angle
C9	O1	C11	O2	-59.9(5)	C9	O1	C11	C12	58.2(5)
C9	O1	C11	C23	-171.7(3)	C11	O1	C9	C8	-82.4(5)
C11	O1	C9	C10	46.2(5)	C11	O2	C22	C20	127.2(3)
C11	O2	C22	C26	0.4(4)	C22	O2	C11	O1	-87.1(4)
C22	O2	C11	C12	150.3(3)	C22	O2	C11	C23	23.3(4)
C15	N6	C27	C16	15.0(6)	C27	N6	C15	C7	3.9(6)
C27	N6	C15	C17	178.6(4)	C8	C7	C15	N6	-144.3(4)
C8	C7	C15	C17	41.1(6)	C15	C7	C8	C9	60.4(5)
C15	C7	C8	C13	-171.7(4)	C8	C7	C16	C27	150.5(3)
C16	C7	C8	C9	-54.5(5)	C16	C7	C8	C13	73.4(5)
C8	C7	C18	C25	61.1(5)	C18	C7	C8	C9	-178.8(3)
C18	C7	C8	C13	-50.9(5)	C15	C7	C16	C27	27.4(5)
C16	C7	C15	N6	-20.8(6)	C16	C7	C15	C17	164.7(4)
C15	C7	C18	C25	-174.6(4)	C18	C7	C15	N6	95.9(5)
C18	C7	C15	C17	-78.7(6)	C16	C7	C18	C25	-65.5(5)
C18	C7	C16	C27	-87.4(4)	C7	C8	C9	O1	-34.3(5)
C7	C8	C9	C10	-163.2(3)	C7	C8	C13	C21	25.3(6)
C9	C8	C13	C21	154.2(4)	C13	C8	C9	O1	-162.0(4)
C13	C8	C9	C10	69.1(5)	O1	C9	C10	O3	-118.8(4)
O1	C9	C10	C14	74.3(5)	C8	C9	C10	O3	10.5(5)
C8	C9	C10	C14	-156.4(3)	O3	C10	C14	O4	-96.5(5)
O3	C10	C14	C19	145.6(4)	C9	C10	C14	O4	70.4(4)
C9	C10	C14	C19	-47.6(5)	O1	C11	C12	C17	56.1(5)
O1	C11	C23	C26	77.4(4)	O2	C11	C12	C17	175.7(3)
O2	C11	C23	C26	-36.9(4)	C12	C11	C23	C26	-155.8(4)
C23	C11	C12	C17	-65.8(5)	C11	C12	C17	O5	88.0(4)
C11	C12	C17	C15	-39.6(5)	C8	C13	C21	C24	171.9(4)
C8	C13	C21	C25	-4.8(8)	O4	C14	C19	C20	163.5(4)
C10	C14	C19	C20	-79.8(6)	N6	C15	C17	O5	-2.2(7)
N6	C15	C17	C12	120.8(5)	C7	C15	C17	O5	172.1(4)
C7	C15	C17	C12	-64.9(6)	C7	C16	C27	N6	-26.9(5)
C7	C18	C25	C21	-41.8(6)	C14	C19	C20	C22	83.1(5)
C14	C19	C20	C28	-153.8(4)	C19	C20	C22	O2	-52.4(5)
C19	C20	C22	C26	66.8(5)	C28	C20	C22	O2	-176.9(4)
C28	C20	C22	C26	-57.7(5)	C13	C21	C24	C29	-174.2(5)
C13	C21	C25	C18	12.5(7)	C24	C21	C25	C18	-164.3(4)
C25	C21	C24	C29	2.6(8)	O2	C22	C26	C23	-23.4(4)

Table S25. Torsion angles (^{o}) (continued)

atom1	atom2	atom3	atom4	angle	atom1	atom2	atom3	atom4	angle
C20	C22	C26	C23	-144.9(4)	C11	C23	C26	C22	36.3(4)

Table S26. Possible hydrogen bonds

Donor	H	Acceptor	D...A	D-H	H...A	D-H...A	
O4	H4	$N6^{1}$	2.795(5)	0.82	2.01	160.28	
O5	H30	$O3^{2}$	2.850(6)	0.80	2.21	136.49	
O5	H30	N6	2.745(6)	0.80	2.34	112.46	intramol.

Symmetry Operators:

(1) X+1,Y,Z (2) X-1,Y,Z

Table S27. Intramolecular contacts less than 3.60 Å

atom	atom	distance	atom	atom	distance
O1	O3	3.396(4)	O1	N6	3.284(6)
O1	C7	2.845(6)	O1	C14	3.271(6)
O1	C15	2.673(6)	O1	C16	3.183(6)
O1	C17	3.018(7)	O1	C19	3.212(7)
O1	C22	3.057(6)	O1	C26	2.819(6)
O2	O3	3.090(4)	O2	C9	2.899(5)
O2	C10	2.554(5)	O2	C14	2.991(5)
O2	C19	2.886(6)	O3	O4	3.086(5)
O3	C8	2.748(6)	O3	C11	3.537(5)
O3	C12	3.568(6)	O3	C13	3.079(6)
O4	C9	3.067(6)	O5	N6	2.745(6)
O5	C11	3.174(7)	O5	C23	3.244(7)
N6	C12	3.543(7)	N6	C18	3.217(6)
C7	C12	3.325(7)	C7	C21	2.901(6)
C8	C11	3.254(6)	C8	C12	3.087(6)
C8	C17	3.143(6)	C8	C25	2.968(7)
C9	C12	3.018(7)	C9	C15	3.127(7)
C9	C16	3.088(8)	C9	C17	3.574(7)
C9	C19	3.220(7)	C10	C11	2.921(6)
C10	C12	3.432(7)	C10	C13	3.115(7)
C10	C20	3.409(7)	C10	C22	3.517(6)
C11	C15	2.964(8)	C11	C20	3.490(8)
C13	C16	3.208(7)	C13	C18	2.824(8)
C14	C22	3.336(6)	C15	C23	3.568(7)
C16	C21	3.457(7)	C16	C25	3.094(7)
C17	C18	3.331(7)	C17	C23	3.245(8)
C18	C27	3.173(8)	C19	C26	3.217(7)
C25	C29	3.000(8)	C26	C28	3.033(9)

Table S28. Intramolecular contacts less than 3.60 Å involving hydrogens

atom	atom	distance	atom	atom	distance
O1	H8	2.821	O1	H12A	2.705
O1	H12B	3.280	O1	H16A	2.720
O1	H19B	2.581	O1	H23A	2.398
O1	H23B	3.177	O1	H26B	2.668
O2	H12A	2.474	O2	H12B	2.538
O2	H14	3.271	O2	H19B	2.893
O2	H20	2.653	O2	H23A	3.165
O2	H23B	2.722	O2	H26A	3.162
O2	H26B	2.866	O3	H4	2.855
O3	H8	2.434	O3	H9	3.001
O3	H12A	2.792	O3	H13	2.765
O3	H14	2.474	O4	H9	2.686
O4	H13	3.448	O4	H19A	2.406
O4	H19B	2.649	O5	H12A	3.123
O5	H12B	2.347	O5	H23A	3.135
O5	H23B	3.001	N6	H16A	2.852
N6	H16B	3.195	N6	H17	3.009
N6	H18A	2.994	N6	H23A	3.135
N6	H30	2.335	C7	H9	2.891
C7	H12A	3.252	C7	H13	3.330
C7	H17	2.829	C7	H25A	2.884
C7	H25B	3.358	C7	H27A	2.856
C7	H27B	3.208	C8	H12A	2.651
C8	H16A	2.656	C8	H16B	3.038
C8	H17	3.262	C8	H18A	3.319
C8	H18B	2.641	C8	H25A	3.523
C9	H4	3.225	C9	H12A	2.762
C9	H13	2.616	C9	H14	3.453
C9	H16A	2.655	C9	H19B	2.809
C10	H4	2.398	C10	H8	2.603
C10	H12A	2.924	C10	H13	2.824
C10	H19A	3.390	C10	H19B	2.724
C10	H20	3.483	C11	H8	3.148
C11	H9	3.243	C11	H17	3.338
C11	H19B	3.224	C11	H22	2.976
C11	H26A	3.200	C11	H26B	2.731
C12	H8	2.600	C12	H23A	3.019

Table S28. Intramolecular contacts less than 3.60 Å involving hydrogens (continued)

atom	atom	distance	atom	atom	distance
C12	H23B	2.722	C12	H30	2.998
C13	H9	2.565	C13	H16A	3.278
C13	H16B	3.206	C13	H18B	3.067
C13	H24	2.581	C13	H25A	3.020
C13	H25B	3.146	C14	H9	2.713
C14	H20	2.620	C15	H8	2.714
C15	H12A	2.912	C15	H12B	3.342
C15	H16A	2.786	C15	H16B	3.154
C15	H18A	2.616	C15	H18B	2.697
C15	H23A	3.216	C15	H27A	2.813
C15	H27B	2.983	C15	H30	2.372
C16	H8	3.390	C16	H9	2.942
C16	H18A	2.686	C16	H18B	3.360
C16	H25A	2.929	C17	H8	2.799
C17	H18A	3.550	C17	H18B	3.080
C17	H23A	3.218	C17	H23B	3.345
C18	H8	2.643	C18	H16A	3.338
C18	H16B	2.574	C18	H17	3.037
C18	H27A	3.151	C19	H4	3.085
C19	H9	3.183	C19	H22	3.370
C19	H26B	2.863	C19	H28A	2.673
C19	H28B	3.352	C19	H28C	2.755
C20	H14	2.690	C20	H26A	3.089
C20	H26B	2.665	C21	H8	2.920
C21	H16B	3.119	C21	H18A	3.341
C21	H18B	2.875	C21	H29A	2.699
C21	H29B	3.323	C22	H14	3.582
C22	H19A	3.327	C22	H19B	2.600
C22	H23A	3.243	C22	H23B	2.777
C22	H28A	3.302	C22	H28B	2.649
C22	H28C	2.649	C23	H12A	3.392
C23	H12B	2.779	C23	H22	2.830
C24	H13	2.549	C24	H25A	2.868
C24	H25B	2.672	C25	H8	3.300
C25	H13	3.306	C25	H16B	2.674
C25	H24	3.459	C25	H29A	2.692
C26	H19B	2.793	C26	H20	3.406

Table S28. Intramolecular contacts less than 3.60 Å involving hydrogens (continued)

atom	atom	distance	atom	atom	distance
C26	H28B	3.287	C26	H28C	2.682
C27	H18A	2.875	C28	H19A	2.531
C28	H19B	2.892	C28	H22	2.679
C28	H26A	3.096	C28	H26B	2.976
C29	H25A	3.136	C29	H25B	2.752
H4	H9	2.901	H4	H13	2.987
H4	H14	2.216	H4	H19A	3.186
H4	H19B	3.385	H8	H9	2.786
H8	H12A	1.954	H8	H12B	3.441
H8	H13	2.533	H8	H16A	3.544
H8	H17	2.727	H8	H18A	3.511
H8	H18B	2.439	H9	H13	2.537
H9	H16A	2.293	H9	H16B	3.428
H9	H19B	2.667	H12A	H17	2.198
H12A	H23B	3.596	H12B	H17	2.431
H12B	H23A	3.339	H12B	H23B	2.563
H12B	H30	3.125	H13	H24	2.312
H14	H19A	2.419	H14	H19B	2.820
H14	H20	2.364	H16A	H27A	2.831
H16A	H27B	2.272	H16B	H18A	2.672
H16B	H18B	3.511	H16B	H25A	2.251
H16B	H25B	3.587	H16B	H27A	2.270
H16B	H27B	2.597	H17	H18A	3.276
H17	H18B	2.533	H17	H30	2.276
H18A	H25A	2.256	H18A	H25B	2.433
H18A	H27A	2.594	H18B	H25A	2.814
H18B	H25B	2.256	H19A	H20	2.487
H19A	H26B	3.436	H19A	H28A	2.331
H19A	H28B	3.433	H19A	H28C	2.753
H19B	H20	2.810	H19B	H22	3.536
H19B	H26A	3.571	H19B	H26B	2.232
H19B	H28A	3.184	H19B	H28C	2.797
H20	H22	2.266	H20	H26B	3.595
H20	H28A	2.352	H20	H28B	2.313
H20	H28C	2.823	H22	H23B	2.876
H22	H26A	2.234	H22	H26B	2.816
H22	H28A	3.544	H22	H28B	2.493

Table S28. Intramolecular contacts less than 3.60 Å involving hydrogens (continued)

atom	atom	distance	atom	atom	distance
H22	H28C	2.978	H23A	H26A	2.555
H23A	H26B	2.308	H23A	H30	3.313
H23B	H26A	2.311	H23B	H26B	2.858
H23B	H30	3.467	H24	H29A	2.854
H24	H29B	2.283	H25A	H29A	2.743
H25B	H29A	2.252	H26A	H28B	3.133
H26A	H28C	2.647	H26B	H28B	3.469
H26B	H28C	2.439			

Table S29. Intermolecular contacts less than 3.60 Å

atom	atom	distance	atom	atom	distance
O2	C28^{1}	3.509(7)	O3	O5^{2}	2.850(6)
O3	C16^{3}	3.503(6)	O3	C28^{1}	3.184(7)
O4	N6^{2}	2.795(5)	O4	C18^{4}	3.352(7)
O4	C27^{2}	3.399(6)	O5	O3^{5}	2.850(6)
O5	C28^{6}	3.413(7)	N6	O4^{5}	2.795(5)
C12	C24^{3}	3.555(7)	C13	C16^{3}	3.538(7)
C16	O3^{4}	3.503(6)	C16	C13^{4}	3.538(7)
C18	O4^{3}	3.352(7)	C24	C12^{4}	3.555(7)
C27	O4^{5}	3.399(6)	C28	O2^{7}	3.509(7)
C28	O3^{7}	3.184(7)	C28	O5^{8}	3.413(7)

Symmetry Operators:

(1) -X+1,Y+1/2-1,-Z
(2) X+1,Y,Z
(3) -X+1,Y+1/2-1,-Z+1
(4) -X+1,Y+1/2,-Z+1
(5) X-1,Y,Z
(6) -X,Y+1/2-1,-Z
(7) -X+1,Y+1/2,-Z
(8) -X,Y+1/2,-Z

Table S30. Intermolecular contacts less than 3.60 Å involving hydrogens

atom	atom	distance	atom	atom	distance
O2	H19A[1]	3.424	O2	H28A[1]	2.598
O3	H16B[2]	2.661	O3	H25A[2]	3.424
O3	H28A[1]	2.830	O3	H28B[1]	3.021
O3	H28C[1]	3.172	O3	H30[3]	2.213
O4	H18A[4]	3.102	O4	H18B[4]	2.934
O4	H23A[3]	3.131	O4	H25B[4]	2.963
O4	H27B[3]	3.042	O4	H30[3]	3.327
O5	H4[5]	3.328	O5	H14[5]	2.971
O5	H24[2]	3.545	O5	H26A[6]	3.523
O5	H27A[7]	3.000	O5	H28B[6]	3.078
O5	H28C[6]	2.900	N6	H4[5]	2.010
N6	H13[5]	3.347	N6	H14[5]	3.553
C8	H16A[2]	3.536	C8	H16B[2]	3.512
C9	H18B[4]	3.473	C9	H25B[4]	3.408
C10	H28A[1]	3.254	C10	H30[3]	2.972
C12	H24[2]	3.595	C13	H16A[2]	2.921
C13	H16B[2]	3.349	C13	H17[4]	3.588
C13	H18B[4]	3.522	C14	H23A[3]	3.382
C14	H25B[4]	3.532	C14	H28C[1]	3.569
C14	H30[3]	3.154	C15	H4[5]	3.144
C16	H8[4]	3.369	C16	H13[4]	3.563
C17	H24[2]	3.360	C17	H27A[7]	3.121
C18	H4[2]	3.535	C18	H9[2]	3.247
C18	H27B[7]	3.436	C19	H25B[4]	3.172
C21	H9[2]	3.498	C21	H12A[4]	3.440
C21	H16A[2]	2.904	C21	H17[4]	3.521
C22	H19A[1]	3.388	C22	H28A[1]	3.479
C22	H29B[8]	3.452	C23	H14[5]	3.331
C23	H29B[8]	3.403	C24	H12A[4]	3.253
C24	H12B[4]	3.182	C24	H16A[2]	3.160
C24	H17[4]	3.088	C24	H27B[2]	3.505
C25	H9[2]	3.148	C25	H16A[2]	3.584
C25	H19B[2]	3.491	C25	H28B[9]	3.315
C26	H12B[10]	3.594	C26	H29B[8]	3.091
C27	H4[5]	2.724	C27	H17[11]	3.243
C27	H30[11]	3.448	C28	H14[12]	3.426
C28	H20[12]	3.589	C28	H25A[13]	3.215

Table S30. Intermolecular contacts less than 3.60 Å involving hydrogens (continued)

atom	atom	distance	atom	atom	distance
C28	H30^{10}	3.542	C29	H12A^{4}	3.535
C29	H12B^{4}	3.032	C29	H19B^{2}	3.492
C29	H23B^{14}	3.434	C29	H26A^{14}	3.113
C29	H26B^{2}	3.114	H4	O5^{3}	3.328
H4	N6^{3}	2.010	H4	C15^{3}	3.144
H4	C18^{4}	3.535	H4	C27^{3}	2.724
H4	H18A^{4}	3.266	H4	H18B^{4}	2.984
H4	H23A^{3}	3.031	H4	H25B^{4}	3.548
H4	H27A^{3}	3.140	H4	H27B^{3}	2.548
H4	H30^{3}	2.759	H8	C16^{2}	3.369
H8	H16A^{2}	3.048	H8	H16B^{2}	2.794
H9	C18^{4}	3.247	H9	C21^{4}	3.498
H9	C25^{4}	3.148	H9	H18B^{4}	2.540
H9	H25B^{4}	2.563	H12A	C21^{2}	3.440
H12A	C24^{2}	3.253	H12A	C29^{2}	3.535
H12A	H16B^{2}	3.538	H12A	H24^{2}	3.482
H12A	H25A^{2}	3.286	H12A	H28A^{1}	3.216
H12B	C24^{2}	3.182	H12B	C26^{6}	3.594
H12B	C29^{2}	3.032	H12B	H24^{2}	3.278
H12B	H26A^{6}	2.681	H12B	H28A^{1}	3.559
H12B	H29A^{2}	3.400	H12B	H29B^{2}	3.021
H13	N6^{3}	3.347	H13	C16^{2}	3.563
H13	H16A^{2}	3.107	H13	H16B^{2}	3.345
H13	H17^{4}	3.185	H13	H18B^{4}	3.139
H13	H27A^{3}	3.264	H13	H27B^{2}	3.439
H14	O5^{3}	2.971	H14	N6^{3}	3.553
H14	C23^{3}	3.331	H14	C28^{1}	3.426
H14	H23A^{3}	2.745	H14	H23B^{3}	3.029
H14	H28A^{1}	3.243	H14	H28C^{1}	2.800
H14	H30^{3}	2.706	H16A	C8^{4}	3.536
H16A	C13^{4}	2.921	H16A	C21^{4}	2.904
H16A	C24^{4}	3.160	H16A	C25^{4}	3.584
H16A	H8^{4}	3.048	H16A	H13^{4}	3.107
H16A	H18B^{4}	3.410	H16A	H24^{4}	3.336
H16A	H25B^{4}	3.446	H16B	O3^{4}	2.661
H16B	C8^{4}	3.512	H16B	C13^{4}	3.349
H16B	H8^{4}	2.794	H16B	H12A^{4}	3.538

Table S30. Intermolecular contacts less than 3.60 Å involving hydrogens (continued)

atom	atom	distance	atom	atom	distance
H16B	H13^{4}	3.345	H16B	H28B^{9}	3.592
H16B	H30^{11}	3.288	H17	C13^{2}	3.588
H17	C21^{2}	3.521	H17	C24^{2}	3.088
H17	C27^{7}	3.243	H17	H13^{2}	3.185
H17	H24^{2}	2.625	H17	H27A^{7}	2.478
H17	H27B^{7}	3.172	H18A	O4^{2}	3.102
H18A	H4^{2}	3.266	H18A	H24^{5}	3.370
H18A	H27B^{7}	2.817	H18B	O4^{2}	2.934
H18B	C9^{2}	3.473	H18B	C13^{2}	3.522
H18B	H4^{2}	2.984	H18B	H9^{2}	2.540
H18B	H13^{2}	3.139	H18B	H16A^{2}	3.410
H18B	H27A^{7}	3.522	H18B	H27B^{7}	3.177
H19A	O2^{12}	3.424	H19A	C22^{12}	3.388
H19A	H20^{12}	3.009	H19A	H22^{12}	2.774
H19A	H25B^{4}	3.136	H19B	C25^{4}	3.491
H19B	C29^{4}	3.492	H19B	H20^{12}	3.588
H19B	H25B^{4}	2.521	H19B	H29A^{4}	2.788
H20	C28^{1}	3.589	H20	H19A^{1}	3.009
H20	H19B^{1}	3.588	H20	H28A^{1}	3.187
H20	H28C^{1}	3.148	H20	H29A^{13}	3.100
H22	H19A^{1}	2.774	H22	H28A^{1}	3.463
H22	H29B^{8}	2.844	H23A	O4^{5}	3.131
H23A	C14^{5}	3.382	H23A	H4^{5}	3.031
H23A	H14^{5}	2.745	H23B	C29^{8}	3.434
H23B	H14^{5}	3.029	H23B	H26A^{6}	3.329
H23B	H26B^{6}	3.258	H23B	H28C^{6}	3.338
H23B	H29A^{8}	3.351	H23B	H29B^{8}	2.830
H24	O5^{4}	3.545	H24	C12^{4}	3.595
H24	C17^{4}	3.360	H24	H12A^{4}	3.482
H24	H12B^{4}	3.278	H24	H16A^{2}	3.336
H24	H17^{4}	2.625	H24	H18A^{3}	3.370
H24	H27A^{3}	2.890	H24	H27B^{2}	3.025
H25A	O3^{4}	3.424	H25A	C28^{9}	3.215
H25A	H12A^{4}	3.286	H25A	H28A^{9}	3.182
H25A	H28B^{9}	2.461	H25B	O4^{2}	2.963
H25B	C9^{2}	3.408	H25B	C14^{2}	3.532
H25B	C19^{2}	3.172	H25B	H4^{2}	3.548

Table S30. Intermolecular contacts less than 3.60 Å involving hydrogens (continued)

atom	atom	distance	atom	atom	distance
H25B	H9^{2}	2.563	H25B	H16A^{2}	3.446
H25B	H19A^{2}	3.136	H25B	H19B^{2}	2.521
H25B	H28B^{9}	3.393	H26A	O5^{10}	3.523
H26A	C29^{8}	3.113	H26A	H12B^{10}	2.681
H26A	H23B^{10}	3.329	H26A	H29A^{8}	3.507
H26A	H29B^{8}	2.389	H26B	C29^{4}	3.114
H26B	H23B^{10}	3.258	H26B	H29A^{4}	2.789
H26B	H29B^{4}	3.082	H27A	O5^{11}	3.000
H27A	C17^{11}	3.121	H27A	H4^{5}	3.140
H27A	H13^{5}	3.264	H27A	H17^{11}	2.478
H27A	H18B^{11}	3.522	H27A	H24^{5}	2.890
H27A	H30^{11}	2.850	H27B	O4^{5}	3.042
H27B	C18^{11}	3.436	H27B	C24^{4}	3.505
H27B	H4^{5}	2.548	H27B	H13^{4}	3.439
H27B	H17^{11}	3.172	H27B	H18A^{11}	2.817
H27B	H18B^{11}	3.177	H27B	H24^{4}	3.025
H27B	H30^{11}	3.389	H28A	O2^{12}	2.598
H28A	O3^{12}	2.830	H28A	C10^{12}	3.254
H28A	C22^{12}	3.479	H28A	H12A^{12}	3.216
H28A	H12B^{12}	3.559	H28A	H14^{12}	3.243
H28A	H20^{12}	3.187	H28A	H22^{12}	3.463
H28A	H25A^{13}	3.182	H28B	O3^{12}	3.021
H28B	O5^{10}	3.078	H28B	C25^{13}	3.315
H28B	H16B^{13}	3.592	H28B	H25A^{13}	2.461
H28B	H25B^{13}	3.393	H28B	H30^{10}	3.203
H28C	O3^{12}	3.172	H28C	O5^{10}	2.900
H28C	C14^{12}	3.569	H28C	H14^{12}	2.800
H28C	H20^{12}	3.148	H28C	H23B^{10}	3.338
H28C	H30^{10}	3.124	H29A	H12B^{4}	3.400
H29A	H19B^{2}	2.788	H29A	H20^{9}	3.100
H29A	H23B^{14}	3.351	H29A	H26A^{14}	3.507
H29A	H26B^{2}	2.789	H29B	C22^{14}	3.452
H29B	C23^{14}	3.403	H29B	C26^{14}	3.091
H29B	H12B^{4}	3.021	H29B	H22^{14}	2.844
H29B	H23B^{14}	2.830	H29B	H26A^{14}	2.389
H29B	H26B^{2}	3.082	H30	O3^{5}	2.213
H30	O4^{5}	3.327	H30	C10^{5}	2.972

Table S30. Intermolecular contacts less than 3.60 Å involving hydrogens (continued)

atom	atom	distance	atom	atom	distance
H30	$C14^{5}$	3.154	H30	$C27^{7}$	3.448
H30	$C28^{6}$	3.542	H30	$H4^{5}$	2.759
H30	$H14^{5}$	2.706	H30	$H16B^{7}$	3.288
H30	$H27A^{7}$	2.850	H30	$H27B^{7}$	3.389
H30	$H28B^{6}$	3.203	H30	$H28C^{6}$	3.124

Symmetry Operators:

(1) -X+1,Y+1/2-1,-Z
(2) -X+1,Y+1/2-1,-Z+1
(3) X+1,Y,Z
(4) -X+1,Y+1/2,-Z+1
(5) X-1,Y,Z
(6) -X,Y+1/2-1,-Z
(7) -X,Y+1/2-1,-Z+1
(8) X-1,Y,Z-1
(9) X,Y,Z+1
(10) -X,Y+1/2,-Z
(11) -X,Y+1/2,-Z+1
(12) -X+1,Y+1/2,-Z
(13) X,Y,Z-1
(14) X+1,Y,Z+1
